# Taming Heavier Group 14 Imine Analogues: Accessing Tin Nitrogen [Sn=N] Double Bonds and their Cycloaddition/Metathesis Chemistry

**DOI:** 10.1002/anie.202211616

**Published:** 2022-10-25

**Authors:** Malte Fischer, Matthew M. D. Roy, Lewis. L. Wales, Mathias A. Ellwanger, Caitilin McManus, Aisling F. Roper, Andreas Heilmann, Simon Aldridge

**Affiliations:** ^1^ Inorganic Chemistry Laboratory Department of Chemistry University of Oxford South Parks Road Oxford OX1 3QR UK; ^2^ Department of Chemistry Catalysis Research Center and Institute for Silicon Chemistry Technische Universität München 85748 Garching bei München Germany

**Keywords:** Cycloaddition, Main Group Chemistry, Metathesis, Stannaimine, Tin

## Abstract

A systematic study to access stable stannaimines is reported, by combining different heteroleptic stannylenes with a range of organic azides. The reactions of terphenyl‐/hypersilyl‐substituted stannylenes yield the putative tin nitrogen double bond, but is directly followed by 1,2‐silyl migration to give Sn^II^ systems featuring bulky silylamido ligands. By contrast, the transition from a two σ donor ligand set to a mixed σ‐donor/π‐donor scaffold allows access to three new stannaimines which can be handled at room temperature. The reactivity profile of these Sn=N bonded species is crucially dependent on the substituent at the nitrogen atom. As such, the Sn=NMes (Mes=2,4,6‐Me_3_C_6_H_2_) system is capable of activating a broad range of substrates under ambient conditions via 1,2‐addition reactions, [2+2] and [4+2] cycloaddition reactions. Most interestingly, very rare examples of main group multiple bond metathesis reactions are also found to be viable.

## Graphical abstract

A systematic study on taming stannaimines (R_2_Sn=NR) is reported by identifying a suitable supporting ligand framework for stannylenes in combination with organic azides. The nitrogen substituent of these compounds was crucial for allowing a broad range of subsequent reactivity ranging from cycloadditions to multiple bond metathesis reactions, thus highlighting the necessary balance of rendering challenging systems isolable but still reactive.

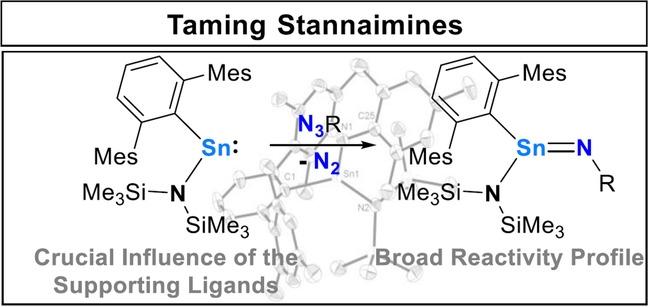

## Introduction

**Scheme 1:**
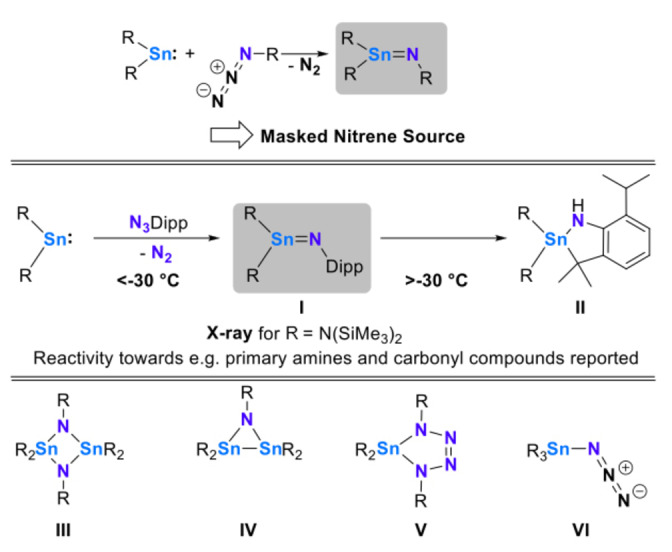
Top: synthetic strategy to access stannaimines; middle: example of the only structurally characterized stannaimine **I** which undergoes intramolecular C−H activation above −30 °C to give **II**; Bottom: Other observed products of reactions of stannylenes with azides **III**–**VI**.

Recent years have seen rapid development in the chemistry of heavier main group compounds containing multiple bonds, with investigations continuously uncovering new reactivity patterns, and even entry into catalytic applications.^[1]^ Despite these developments, there are still significant gaps when it comes to isolable compounds, with new strategies being required to generate ′bottleable′ species, or at least to allow for clean/facile generation for investigation of subsequent chemistry. We have lately become interested in tin pnictogen multiple bonds and have reported on phosphinidene transfer to Sn^II^, which allows for the isolation and characterization of a crystalline stannaphosphene.^[2]^ Lighter homologues which possess multiple bonds between tin and nitrogen, often referred to as either stannaimines or tin‐imido complexes (drawing comparisons to tin as a metalloid), are also rare. To date, there is one crystallographically characterized example (**I**), and even this compound is stable only below −30 °C, undergoing facile intramolecular C−H activation at higher temperatures to give stannacycle **II** (Scheme 1, middle).^[3]^

Potential routes to stannaimines involve the combination of low‐valent stannylenes and organoazides, with the latter acting as masked nitrene sources via extrusion of dinitrogen (Scheme 1, top). To date however, all literature examples following this synthetic protocol lead either to C−H activation, formal dimerization (cf. distannadiazane **III**), azadistanniridine formation (cf. **IV**), reactions with a second equivalent of the organoazide to yield a tetraazastannole (cf. **V**) or rearrangement to give a tin azide (cf. **VI**; Scheme 1, bottom).[^3^, ^4^]

These observations point to the high reactivity of putative tin nitrogen double bonds, evidenced by these “self‐activation” processes or reactions with further azide. As a consequence, only in the case of compound **I** has any subsequent reactivity been reported, focussing on primary amines and carbonyl compounds.

Reflecting the balancing act between obtaining tractable species and systems which might still be expected to show interesting patterns of chemical reactivity, we set out to investigate Sn=N double bonds derived from the reactions between two electronically distinct stannylenes and five organoazides. This approach allows for systematic variation of the σ‐ and π‐donor capabilities of the stannylene supporting framework, and of the steric profile of the putative imine function. As such, it has enabled us to characterize stannaimines which can be handled at room temperature, thereby allowing for studies of reactivity focussing on 1,2‐addition chemistry and cycloaddition processes leading to Sn=N/Sn=E metathesis.

## Results and Discussion

### Reactions of Organoazides with a Silyl‐Stannylene

**Scheme 2:**
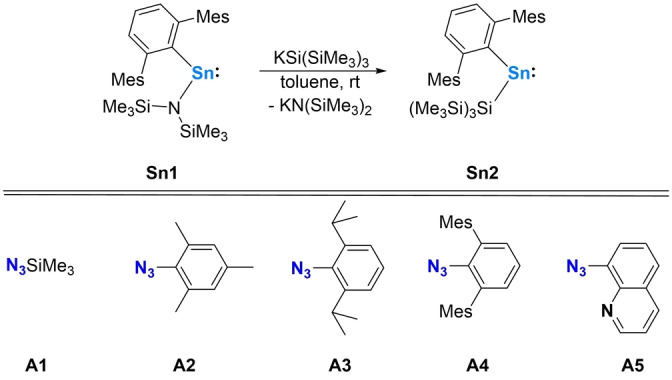
Top: Synthesis of the mixed terphenyl‐/hypersilyl‐substituted stannylene **Sn2** from the mixed terphenyl‐/amido‐substituted stannylene **Sn1**; bottom: set of azides **A1**–**A5** used in this study.

Initial investigations focussed on the heteroleptic terphenyl‐/hypersilyl‐substituted stannylene ^Mes^TerSn(Si(SiMe_3_)_3_) (**Sn2**) which can be accessed by reacting the previously reported amido/terphenyl analogue ^Mes^TerSn(N(SiMe_3_)_2_)^[2]^ (**Sn1**) with (thf)_2_K(Si(SiMe_3_)_3_) (^Mes^Ter=−C_6_H_3_‐2,6(C_6_H_2_‐2,4,6‐Me_3_)_2_; Scheme 2, top).

**Figure 1:**
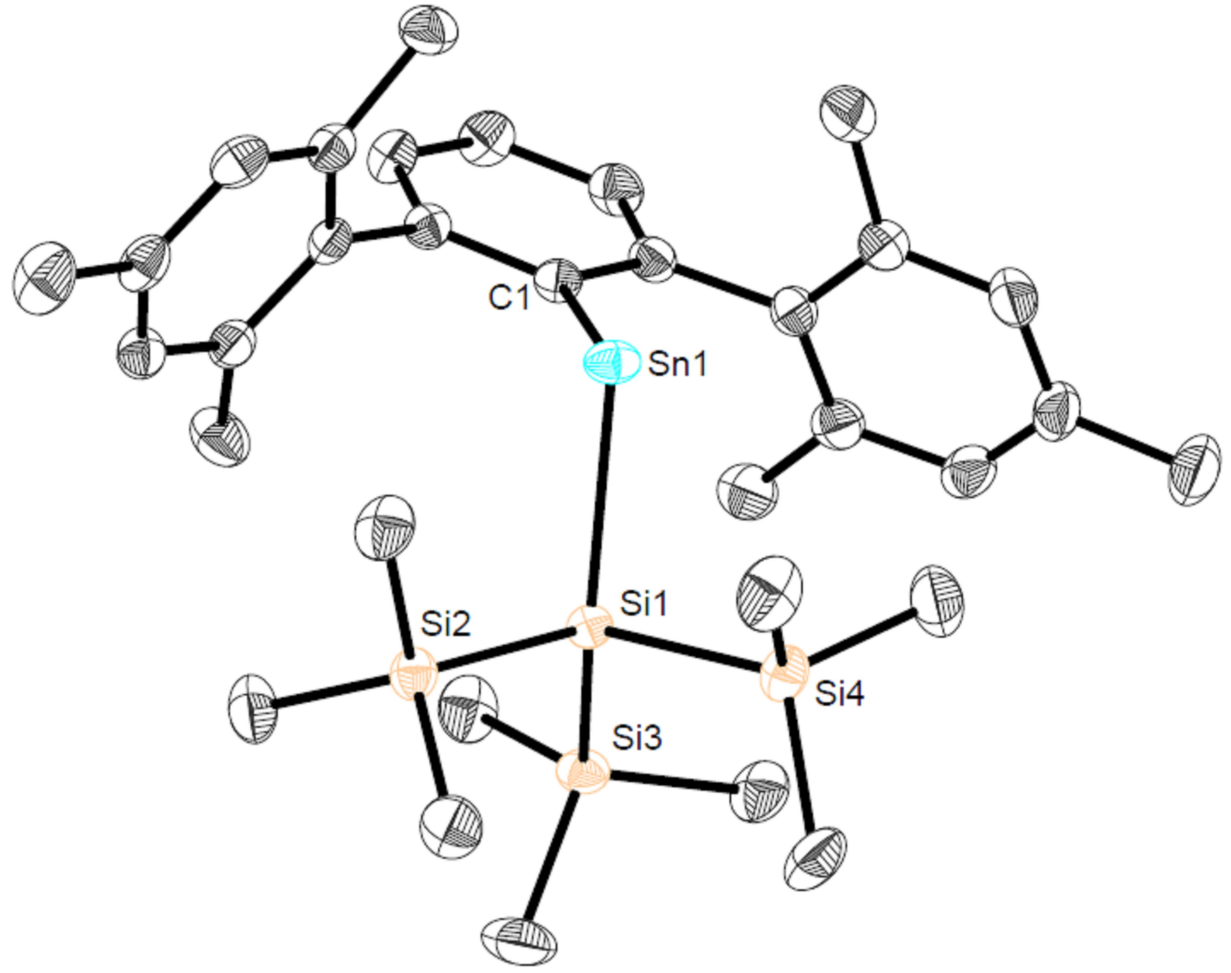
Molecular structure of ^Mes^TerSnSi(SiMe_3_)_3_ (**Sn2**) in the crystal. Thermal ellipsoids are drawn at the 50 % probability level (hydrogen atoms have been omitted for clarity). Selected bond lengths [Å] and angles [deg]: Sn1−Si1 2.6407(7), Sn1−C1 2.189(3), C1−Sn1−Si1 109.75(6).

We hypothesized that the kinetically stabilizing features associated with the steric bulk of the terphenyl ligand class^[5]^ allied to the presence of a second strong σ‐donor substituent −(Si(SiMe_3_)_3_)^[6]^ should give access to a stannylene with a wide C−Sn−Si angle and a small HOMO–LUMO gap. It was thought that this would likely render **Sn2** highly reactive towards organoazides while also offering sufficient shielding towards dimerization of the product stannaimine. **Sn2** was obtained as a blue crystalline solid in 87 % isolated yield and characterized by multinuclear NMR, UV/Vis spectroscopy, elemental microanalysis and single crystal X‐ray diffraction. The intense blue colour of **Sn2** is consistent with the measured absorption at *λ*_max_=638 nm (Figure S3^[7]^). The molecular structure of **Sn2** shows a wide C1−Sn1−Si1 bond angle of 109.75(6)° which is similar to that of the recently reported system ^Mes^TerSn(Si(^*t*^Bu)_3_) (113.50(14)°),^[8]^ and characteristic of both significant p‐character in the HOMO and a small HOMO–LUMO gap (Figure 1).

**Scheme 3:**
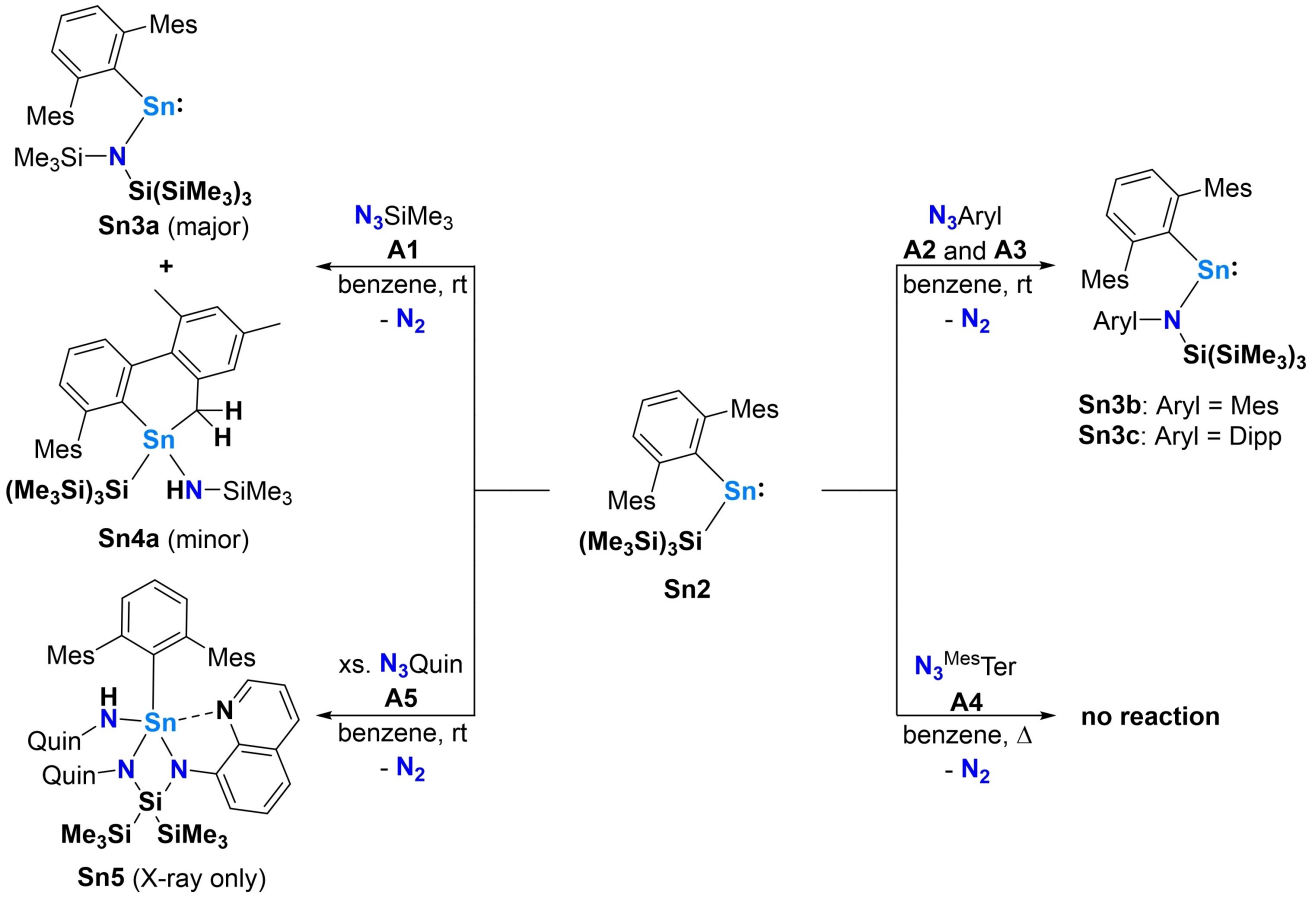
Reactions of **Sn2** with **A1**–**A5**.

A range of organoazides was identified for formal nitrene transfer chemistry including commercially available N_3_SiMe_3_ (**A1**), aryl‐substituted azides of different steric bulk (N_3_Mes (**A2**), N_3_Dipp (**A3**), and N_3_^Mes^Ter (**A4**)) and N_3_Quin (**A5**) which features an additional nitrogen donor functionality designed to stabilize the putative stannaimine through dative Sn−N bond formation (Scheme 2, bottom). Accordingly, the reactivity of **Sn2** with each of **A1**–**A5** was scoped (Scheme 3).

**Figure 2:**
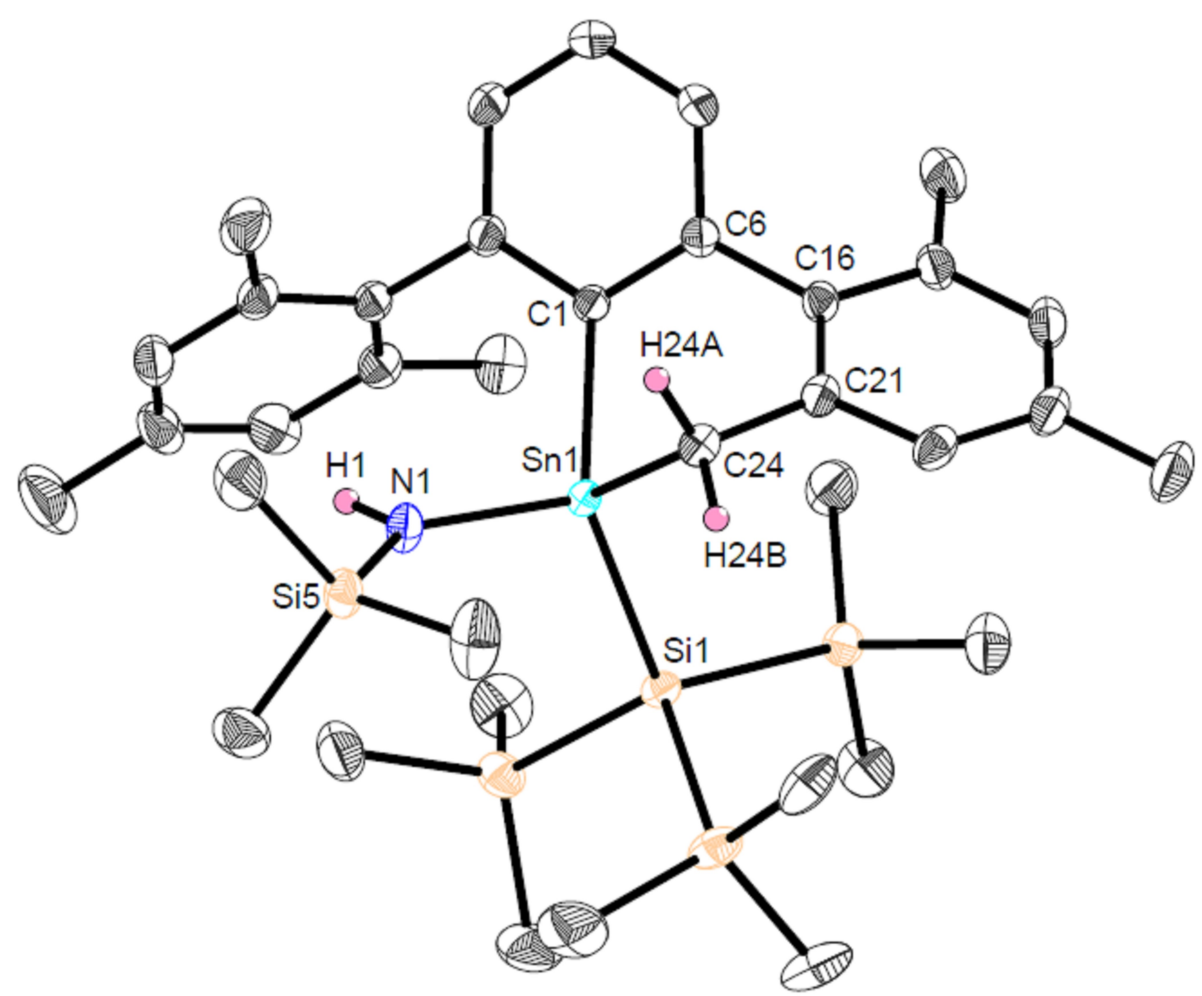
Molecular structure of the C(sp^3^)−H activation product **Sn4a** in the crystal. Thermal ellipsoids are drawn at the 50 % probability level (hydrogen atoms except for H1, H24A and H24B have been omitted for clarity). Selected bond lengths [Å] and angles [deg]: Sn1−N1 2.0445(14), Sn1−C1 2.1754(16), Sn1−C24 2.1715(16), Sn1−Si1 2.5915(4), C21−C24 1.500(2), C1−Sn1−Si1 125.04(4), C1−Sn1−N1 106.37(6).

The reaction of **Sn2** with N_3_SiMe_3_ (**A1**) is accompanied by immediate N_2_ evolution and the formation of two products according to ^1^H NMR spectroscopy, even when the reaction is initiated at low temperatures (Figure S5).^[7]^ Colourless crystals of the minor product suitable for single crystal X‐ray diffraction could be isolated, which revealed the formation of a product derived from intramolecular C(sp^3^)−H activation (**Sn4a**) in which one of the flanking mesityl methyl groups has been activated, resulting in the formation of N−H and Sn−CH_2_ functionalities (Figure 2).

The generation of **Sn4a** can be hypothesized as proceeding via the formation of the putative stannaimine ^Mes^TerSn(Si(SiMe_3_)_3_)=NSiMe_3_ followed by a C−H activation step comparable to the previously reported formation of **II** starting from **I** (Scheme 1).^[3]^

Structurally, the central tin atom in **Sn4a** is four‐coordinate, and in a distorted tetrahedral coordination environment. Both, the Sn1−N1 and Sn1−C24 bond lengths (2.0445(14) Å and 2.1715(16) Å) are diagnostic of the respective single bonds (Sn−C 2.15 Å, Sn−N 2.11 Å),^[9]^ and are in good accordance with the only comparable structurally characterized C−H activation product, derived from the reaction of (Mes*)_2_Sn with N_3_Dipp (cf. 2.084(3) Å and 2.137(4) Å).^[4b]^ In C_6_D_6_ solution the ^1^H NMR spectrum shows the expected diastereotopic doublet signals located at *δ*=2.59 and 2.88 ppm (^2^ *J*_H,H_=11.2 Hz) associated with the methylene group. The NH hydrogen atom gives rise to a resonance at *δ*=−0.99 ppm. The nuclei close to tin show the expected characteristic tin satellites, with these being seen for both the hydrogen atoms of both the methylene group and the NH moiety (e.g. ^2^ *J* ${}_{{}^{119}\mathrm{Sn},H}$ =93.8 Hz, ^2^ *J* ${}_{{}^{117}\mathrm{Sn},H}$ =71.7 Hz for one of the methylene group signals; Figure S9).^[7]^

**Figure 3:**
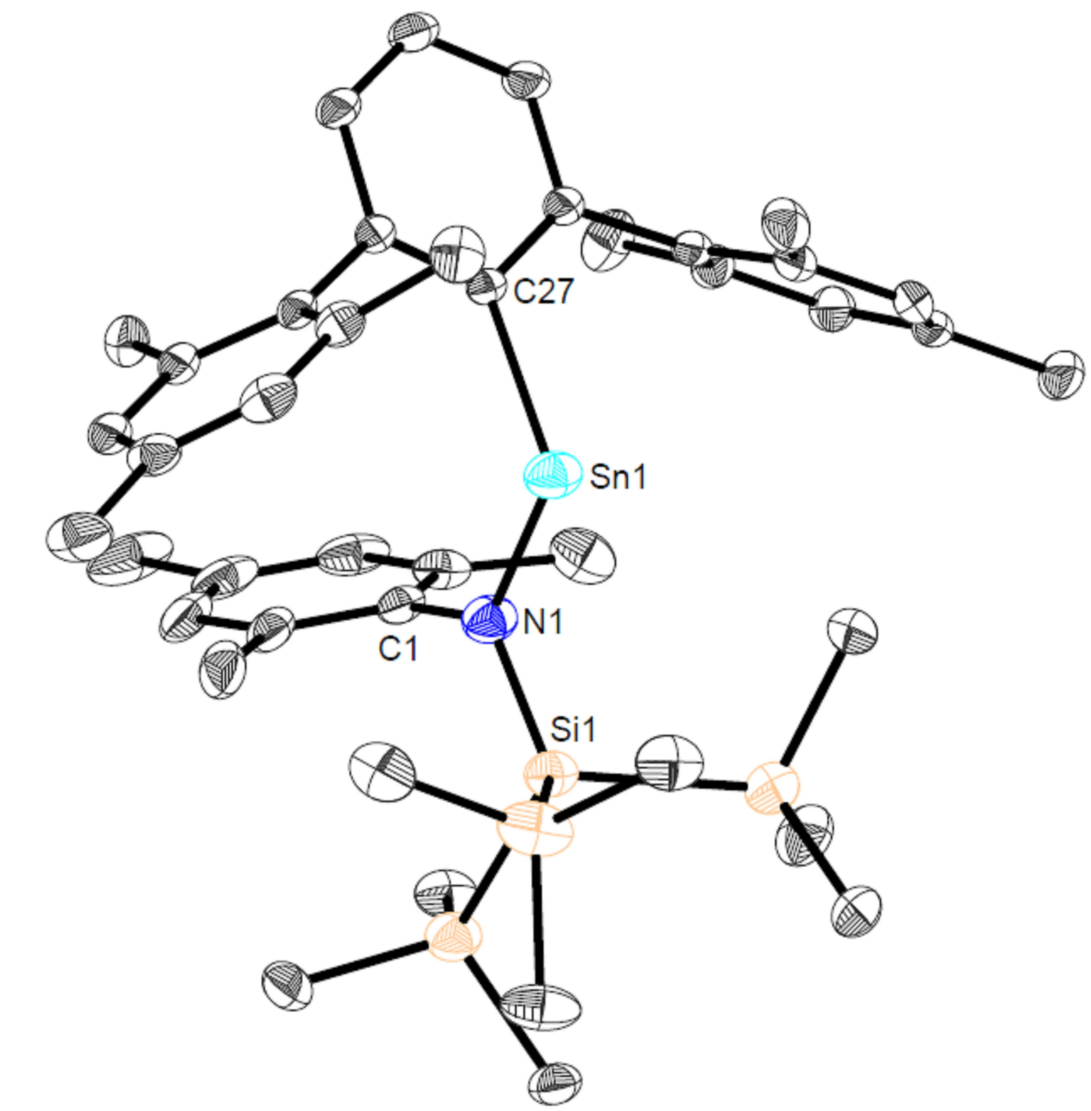
Molecular structure of ^Mes^TerSnN(Mes)Si(SiMe_3_)_3_ (**Sn3b**) in the crystal. Thermal ellipsoids are drawn at the 50 % probability level (hydrogen atoms have been omitted for clarity). Selected bond lengths [Å] and angles [deg]: Sn1−N1 2.079(2), Sn1−C27 2.234(3), C27−Sn1−N1 109.49(9), C1−N1−Sn1 126.02(17), C1−N1−Si1 118.34(17), Si1−N1−Sn1 115.64(11).

Although we were not able to isolate the major product of the reaction in a pure bulk state due to omnipresent co‐crystallization of **Sn4a**, colorless needles of this compound were identified (and separated by crystal picking) which were suitable for single crystal X‐ray diffraction. These were shown to contain the amido/terphenyl stannylene ^Mes^TerSn{N(SiMe_3_)Si(SiMe_3_)_3_} (**Sn3a**) formed by insertion of the N(SiMe_3_) function into the Sn−Si bond of **Sn2** (Scheme 3 and Figure S11). In this context, the reactions of **Sn2** with the aryl‐substituted azides N_3_Mes (**A2**) and N_3_Dipp (**A3**) also result in the clean formation of the formal nitrene insertion products ^Mes^TerSnN(Aryl)Si(SiMe_3_)_3_ (Aryl=Mes (**Sn3b**), Dipp (**Sn3c**)) (Scheme 3). Both **Sn3b** and **Sn3c** could be characterized by single crystal X‐ray diffraction (Figure 3 and Figure S18). Structurally, the Sn−N bond lengths of (on average) 2.09 Å for **Sn3a**–**c** are typical of single bonds and the transition from Sn−Si bonding in the starting material **Sn2** to Sn−N bonding in **Sn3a**–**c** is accompanied by a widening of the C^MesTer^−Sn−N bond angle from 109.75(6)° to 112.03(8)° (**Sn3c**). Since substitution of a strong σ‐donor substituent by a weaker one is expected to lead to more bent carbenoid systems, steric effects are considered to be the primary reason for this observation.

**Figure 4:**
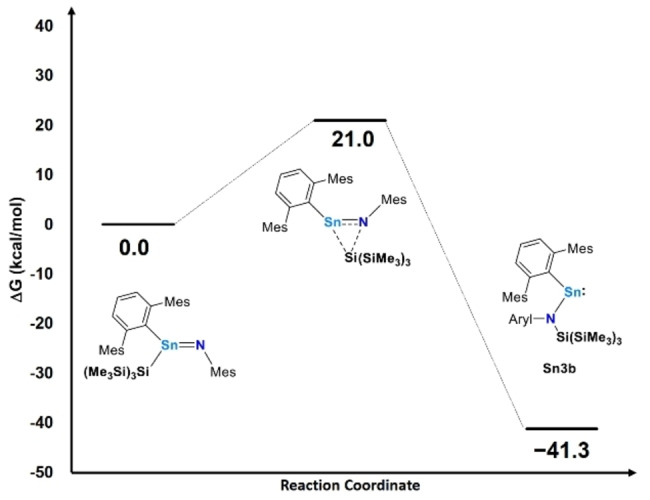
Reaction coordinate for the formation of **Sn3b** from the presumed stannaimine (all energies, G, in kcal mol^−1^).

Of note mechanistically is the fact that each of **Sn3a**–**c** features an aryl(silyl)amido ligand, formed via silyl group migration to the putative Sn=N functionality ‐ highlighting a novel reaction pathway between stannylenes and azides. The formation of **Sn3a**–**c** is hypothesized to involve initial formation of the target stannaimine followed by facile silyl migration. Computationally, we find that this pathway is both kinetically and thermodynamically viable (Figure 4), with an activation barrier of 21 kcal mol^−1^ (for **Sn3b**) and an overall free energy change (Δ*G*=−41.3 kcal mol^−1^) that is highly exergonic.

**Figure 5:**
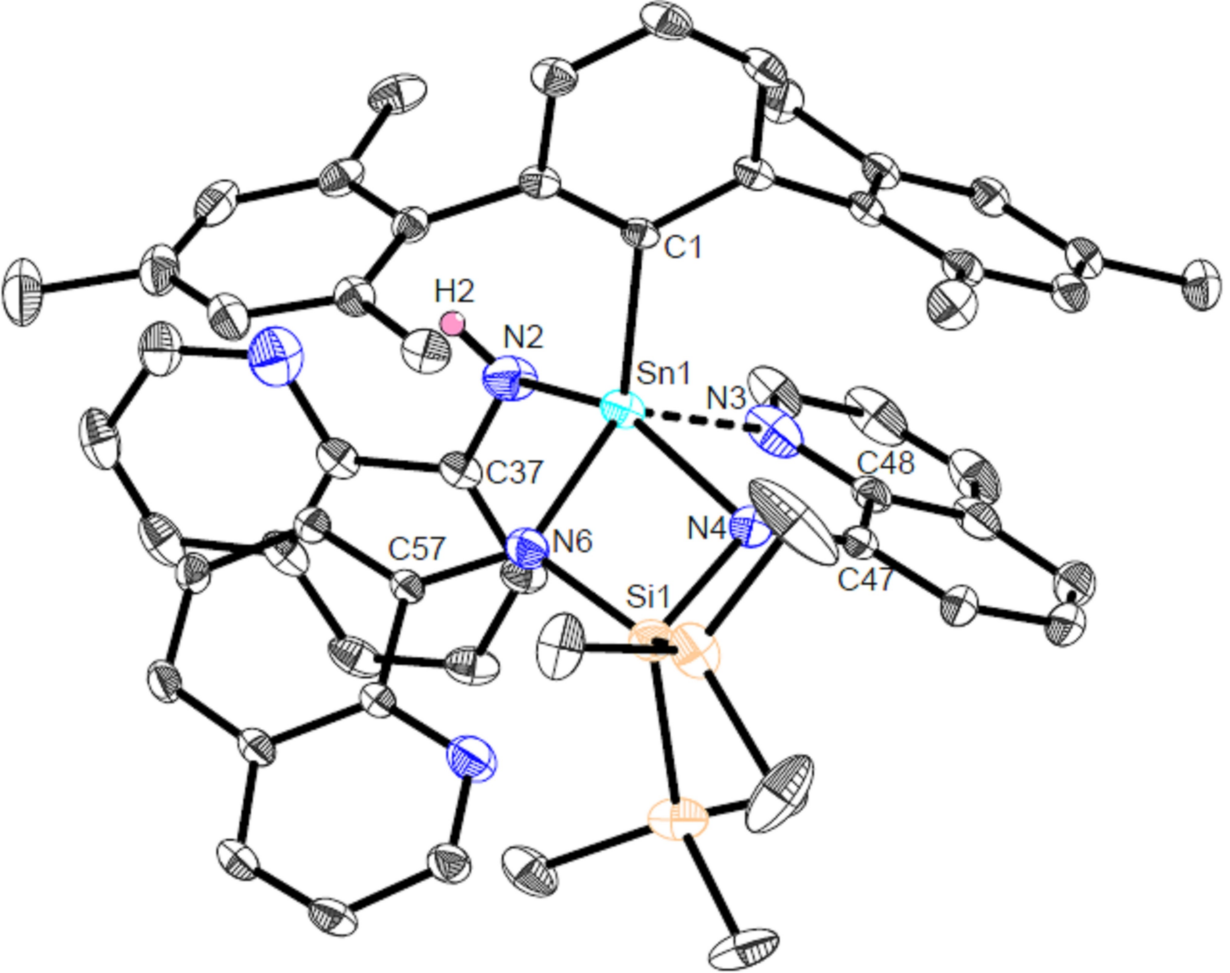
Molecular structure of ^Mes^TerSn(NHQuin)(QuinNSi(SiMe_3_)_2_NQuin) (**Sn5**) in the crystal. Thermal ellipsoids are drawn at the 50 % probability level (hydrogen atoms have been omitted for clarity). Selected bond lengths [Å] and angles [deg]: Sn1−N2 2.0719(14), Sn1−N3 2.3256(15), Sn1−N4 2.0808(13), Sn1−N6 2.1162(12), Sn1−C1 2.1765(15), N4−Si1 1.7965(14), N6−Si1 1.7685(14), N2−Sn1−N6 101.39(5), N6−Sn1−N4 72.06(5), N4−Sn1−N3 74.31(6), C1−Sn1−N2 105.44(6), C1−Sn1−N6 117.90(5), C1−Sn1−N4 108.10(6), C1−Sn1−N3 110.20(5).

We also examined the use of sterically more demanding organoazide reagents and systems featuring additional pendant functionality. However, with **Sn2** and N_3_^Mes^Ter (**A4**), for example, no reaction could be observed even after prolonged heating of the reaction mixture to 80 °C (Figure S19). On the other hand, the reaction of **Sn2** with N_3_Quin(**A5**) in a 1 : 1 stoichiometry resulted in significant amounts of unreacted **Sn2**. The reaction was therefore repeated with an excess of **A5**, resulting in further gas evolution and the formation of a clear orange solution. After work‐up, a small quantity of orange crystals suitable for single crystal X‐ray diffraction were obtained from a saturated *n*‐hexane solution at −30 °C. Crystallographic analysis reveals that a total of three quinoline moieties are incorporated in the product ^Mes^TerSn(NHQuin)(QuinNSi(SiMe_3_)_2_NQuin) (**Sn5**; Figure 5). As can be seen from Figure 5, the reaction between **Sn2** and **A5** results in deconstruction of the hypersilyl functionality in **Sn2** to generate a Si(SiMe_3_)_2_ moiety which bridges two azide‐derived N atoms to form a chelating tridentate *N*,*N*,*N*‐ligand in the product **Sn5**. The third coordination site is the result of one quinoline nitrogen atom additionally coordinating to tin (2.3256(15) Å). The other two tin nitrogen linkages have separations typical of single bonds (2.0808(13) Å and 2.1162(12) Å). The third equivalent of quinoline azide is coordinated as the corresponding amido ligand, so that formally a Me_2_Si=CH_2_ unit is the leaving group in this reaction. Together with the remaining terphenyl ligand, the tin center is in a distorted tetragonal pyramidal coordination environment.

### Synthesis of Stannaimines from an Amido‐Stannylene

None of the above reactions using (silyl)stannylene **Sn2** allowed for the isolation of a stannaimine, with formal nitrene insertion into the Sn−Si bond being commonly encountered. In order to circumvent this problem, we therefore considered the use of stannylene precursor bearing a pendant amido (rather than silyl) substituent. Although likely to be less reactive kinetically, we perceived (from a thermodynamic perspective) that the π donor characteristics of the amido group would complement the strongly σ‐donating terphenyl moiety to stabilize the electron‐deficient stannaimine Sn centre through *both* inductive and resonance effects. The combination of strong σ and strong π donor substituents has recently been employed to great effect in the stabilization of silanone compounds, R_2_Si=O.^[10]^

**Scheme 4:**
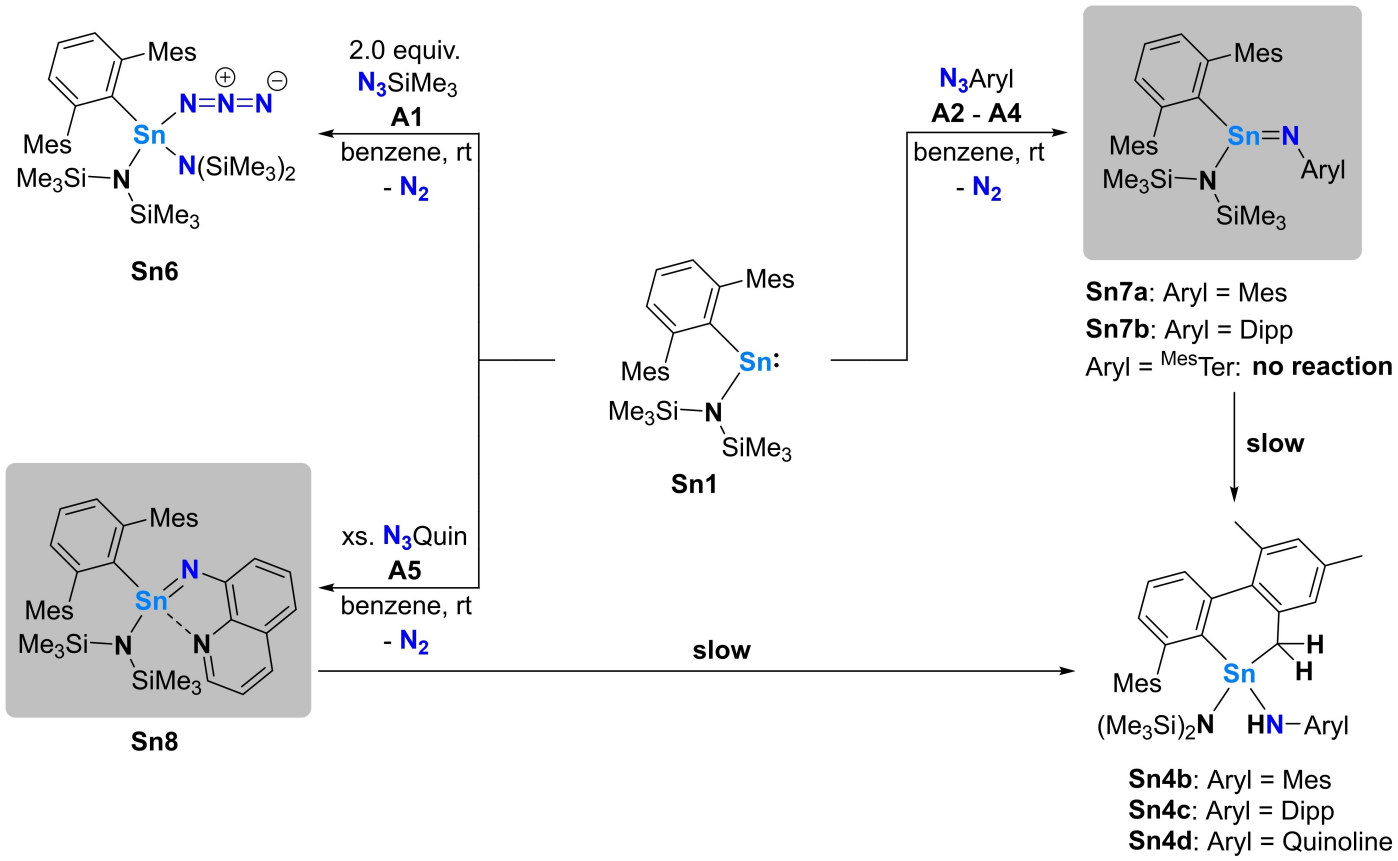
Reactions of **Sn1** with **A1**–**A5**.

Systems of type **Sn3**—bearing sterically very demanding amido substituents—are unreactive towards a second equivalent of the azide substrate, so we targeted sterically less demanding analogues. The stoichiometric reaction of **Sn1** with N_3_SiMe_3_ (**A1**) results in the retention of half an equivalent of unreacted **Sn1**. Addition of a second equivalent of **A1** gives clean conversion to a single product which was unambiguously identified as the azido/bis(amido) Sn^IV^ compound ^Mes^TerSn{N(SiMe_3_)_2_}_2_N_3_ (**Sn6**) by single crystal X‐ray diffraction (Scheme 4 and Figure S25). N−Si bond cleavage of a second molecule of N_3_SiMe_3_ at a putative stannaimine is assumed to be the reaction path, in accordance with the previously reported reaction of Sn{N(SiMe_3_)_2_}_2_ with N_3_SiMe_3_ which yields Sn{N(SiMe_3_)_2_}_3_N_3_.^[4c]^ The structural parameters for **Sn6** are in good agreement with the latter system, and a detailed discussion is not warranted at this point.

**Figure 6:**
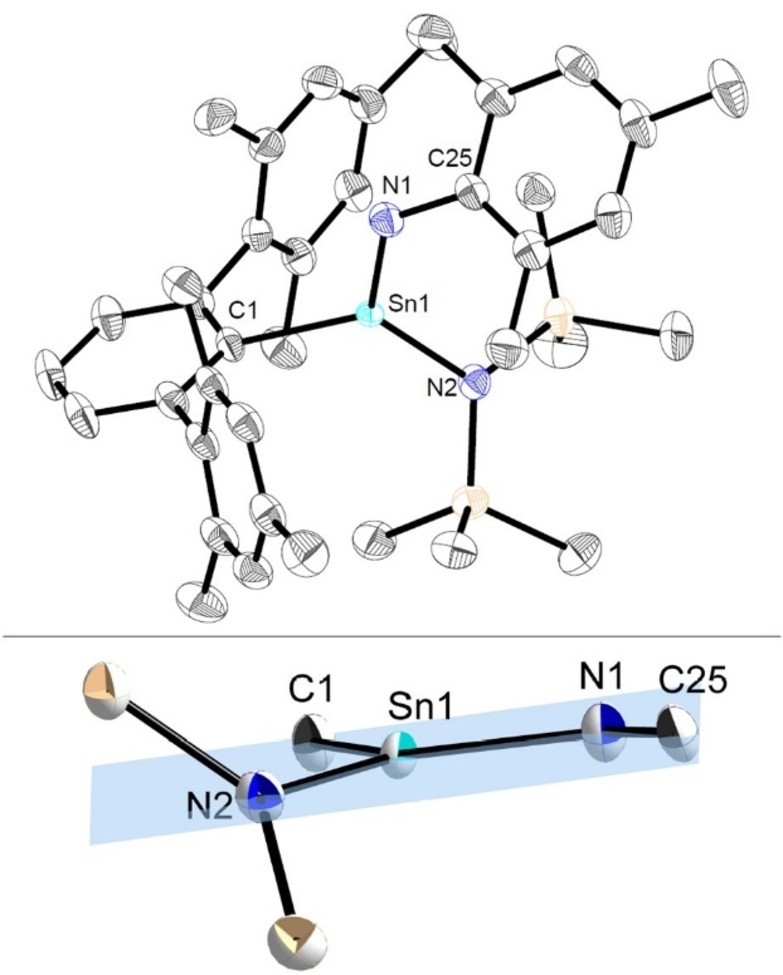
Top: Molecular structure of ^Mes^TerSn(N(SiMe_3_)_2_)=NMes (**Sn7a**) in the crystal. Thermal ellipsoids are drawn at the 50 % probability level (hydrogen atoms have been omitted for clarity). Selected bond lengths [Å] and angles [deg]: Sn1−N1 1.9354(16), Sn1−N2 2.0455(16), Sn1−C1 2.1481(18), N1−C25 1.401(2), C1−Sn1−N1 116.46(7), N1−Sn1−N2 125.17(7), C1−Sn1−N2 118.34(7); bottom: Excerpt of the molecular structure of **Sn7a** in the crystal showing the planarity of the central unit.

The reactions of **Sn1** with the aryl‐substituted azides N_3_Mes (**A2**) or N_3_Dipp (**A3**) occur readily at room temperature, with immediate gas evolution being observed, accompanied by a color change from bright orange/red to dark red. NMR analysis in each case reveals clean formation of a single product. However, on standing at room temperature overnight, significant numbers of additional signals were observed to grow in, accompanied by decolorization of the solution. The initially formed products could, however, be obtained as dark red crystalline materials by crystallization from aliphatic hydrocarbons at −30 °C. In case of the reaction of **Sn1** with **A2**, these crystals were suitable for single crystal X‐ray diffraction, and crystallographic studies reveal the formation of the target stannaimine **Sn7a**, clearly evident by the short Sn1−N1 bond of 1.9354(16) Å and the sum of covalent radii around the tin center of 360° (Figure 6).

**Figure 7:**
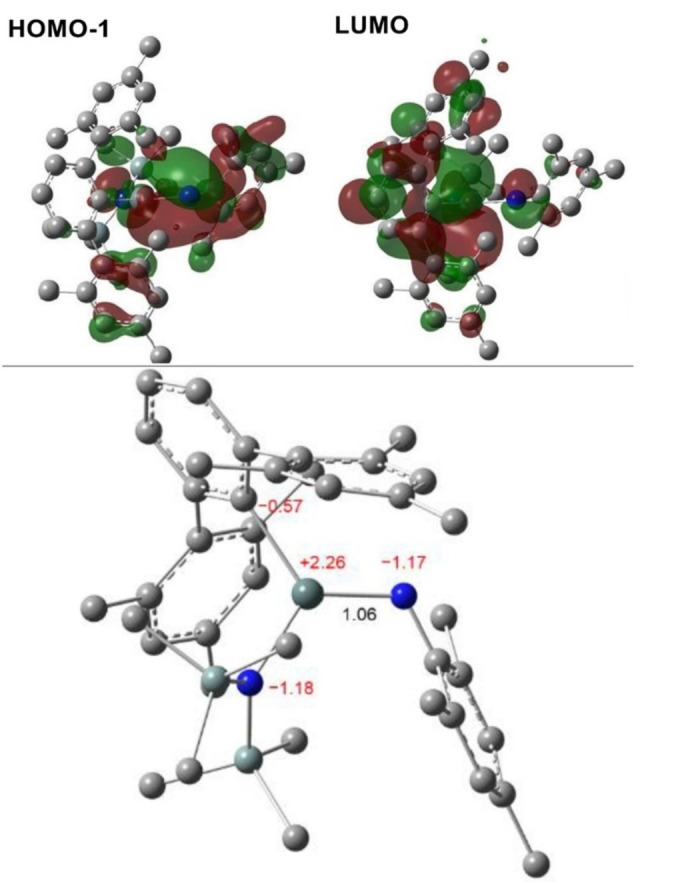
Top: Selected molecular orbitals of the optimized structure of **Sn7a**; bottom: Optimized structure of **Sn7a** with the tin‐nitrogen Wiberg bond index (shown in black and selected natural atomic charges (shown in red).

To gain further insight into the bonding situation in stannaimine systems, we carried out quantum chemical calculations on the less computationally demanding derivative **Sn7a**. The HOMO‐1 and LUMO are best described as Sn=N π‐bonding and π*‐antibonding orbitals, respectively and the Kohn–Sham molecular orbitals of **Sn7a** reveal that the Sn−N interaction is largely polarized towards nitrogen (Figure 7, top). This is further demonstrated by Natural Bonding Orbital (NBO) analysis. Two NBOs contribute to the Sn−N bonding in **Sn7a**, both of which are highly polarized (78 % each) towards nitrogen and account for a total of 2.06 electrons between tin and nitrogen (Table S8). This finding is consistent with the calculated Sn−N Wiberg bond index, which was found to be only slightly greater than unity (1.06) (Figure 7, bottom). Taken together these data suggest that the bonding between tin and nitrogen is highly polarized and possesses a high degree of ionic character, consistent with the significant electronegativity difference between the two atoms. This polarity can also be seen in the NBO charges calculated for **Sn7a** (Sn, +2.26; N, −1.17; Figure 7, bottom). Furthermore, the electron density at the bond critical point (BCP) located between Sn and N using Atoms in Molecules (QTAIM) analysis is relatively low [*ρ*(*r*)=0.1338 e Å^−3^] (Figure S82). This finding, along with the positive Laplacian of the electron density at this point [∇^2^ *ρ*(*r*)=0.5142 e Å^−5^] are indicative of a highly electrostatic interaction. Nonetheless, the non‐spherical nature of the BCP (*ϵ*=0.122) is consistent with a degree of π delocalization between these two atoms.

The observation of additional signals in the ^1^H NMR spectra of **Sn7a** and **Sn7b** over time is due to intramolecular C(sp^3^)−H activation across the Sn=N bond. This benzylic C−H activation (effectively deprotonation) by the basic imido group within the polar Sn=N unit results in the formation of the corresponding amido functionalities, and coordination of a methylene group to the tin center to give **Sn4b**,**c** (Scheme 4). **Sn4b**,**c** could both be characterized by single crystal X‐ray diffraction and the molecular structures are shown in Figures S32 and S38. The structural parameters of these six‐membered stannacycles are consistent with those measured for **Sn4a** (Figure 2). In contrast to the only other isolated stannaimine [Sn(N(SiMe_3_)_2_)=NDipp],^[4c]^ **Sn7a**,**b** can be handled at room temperature for prolonged periods allowing for systematic investigation of their reaction chemistry. In this context, storage of a benzene solution of **Sn7a** for four hours at room temperature yields only 10 % of **Sn4b**.

**Figure 8:**
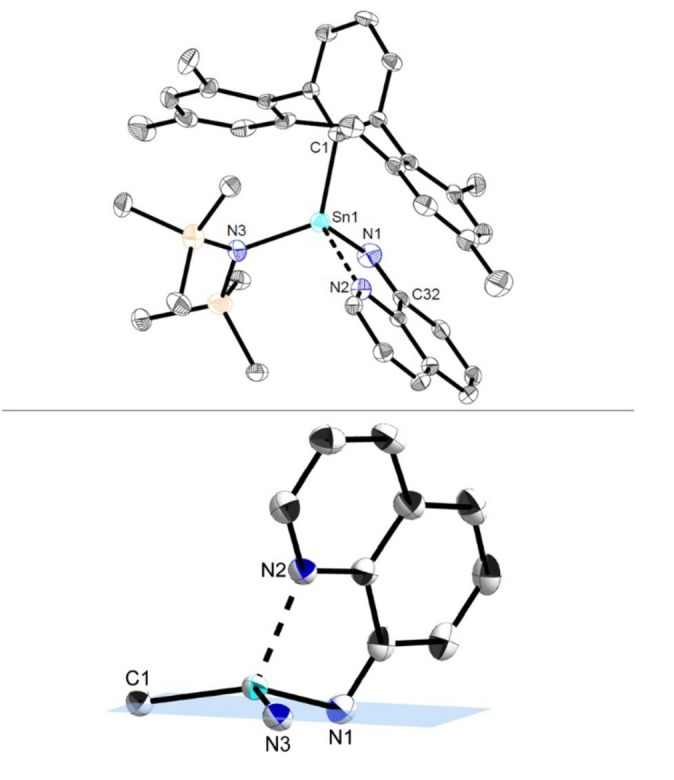
Top: Molecular structure of ^Mes^TerSn(N(SiMe_3_)_2_)=NQuin (**Sn8**) in the crystal. Thermal ellipsoids are drawn at the 50 % probability level (hydrogen atoms have been omitted for clarity). Selected bond lengths [Å] and angles [deg]: Sn1−N1 2.022(4), Sn1−N2 2.198(3), Sn1−N3 2.032(3), Sn1−C1 2.158(4), N1−Sn1−N2 93.09(13), N1−Sn1−N3 114.71(14); bottom: Excerpt of the molecular structure of **Sn8** in the crystal showing the coordination environment at the tin center.

Finally, to probe whether an internal tethered donor unit could be employed to further stabilize a stannaimine, the reaction of **Sn1** with N_3_Quin (**A5**) was investigated. On combining the two compounds in benzene, an immediate colour change to dark green was observed, with ^1^H NMR analysis confirming clean formation of a single product. This species was shown by X‐ray crystallography to be the corresponding stannaimine **Sn8**, as expected stabilized by dative coordination of the pendant quinoline nitrogen atom (Scheme 4, Figure 8). As a result of the dative coordination of the quinoline nitrogen atom, the tin center in this case is four‐coordinate and in a distorted tetrahedral coordination environment. The Sn1−N1 bond length of 2.022(4) Å is significantly elongated when compared to stannaimine **Sn7a** (1.9354(16) Å). However, despite the tin center being datively stabilized and four‐coordinate, similar intramolecular C(sp^3^)−H activation is observed at room temperature, as seen for **Sn7a,b**, yielding the corresponding complex **Sn4d** which was also characterized by multinuclear NMR spectroscopy and single crystal X‐ray diffraction.[^7^, ^11^]

### Reactivity Studies of Stannaimines

For the only previously reported example of a stannaimine (**I**), 1,2‐addition reactions with primary amines and cycloaddition reactions with carbonyl compounds were investigated, but no structural data for cycloaddition products were reported.^[3b]^ With this in mind, we perceived that the more stable stannaimines **Sn7a,b** and **Sn8** might provide a convenient basis for systematically investigating the reactivity of this substance class. In a first exploration of reactivity, **Sn7a,b** and **Sn8** were reacted with phenylacetylene in attempts to generate the corresponding 1,2‐addition products.

**Scheme 5:**
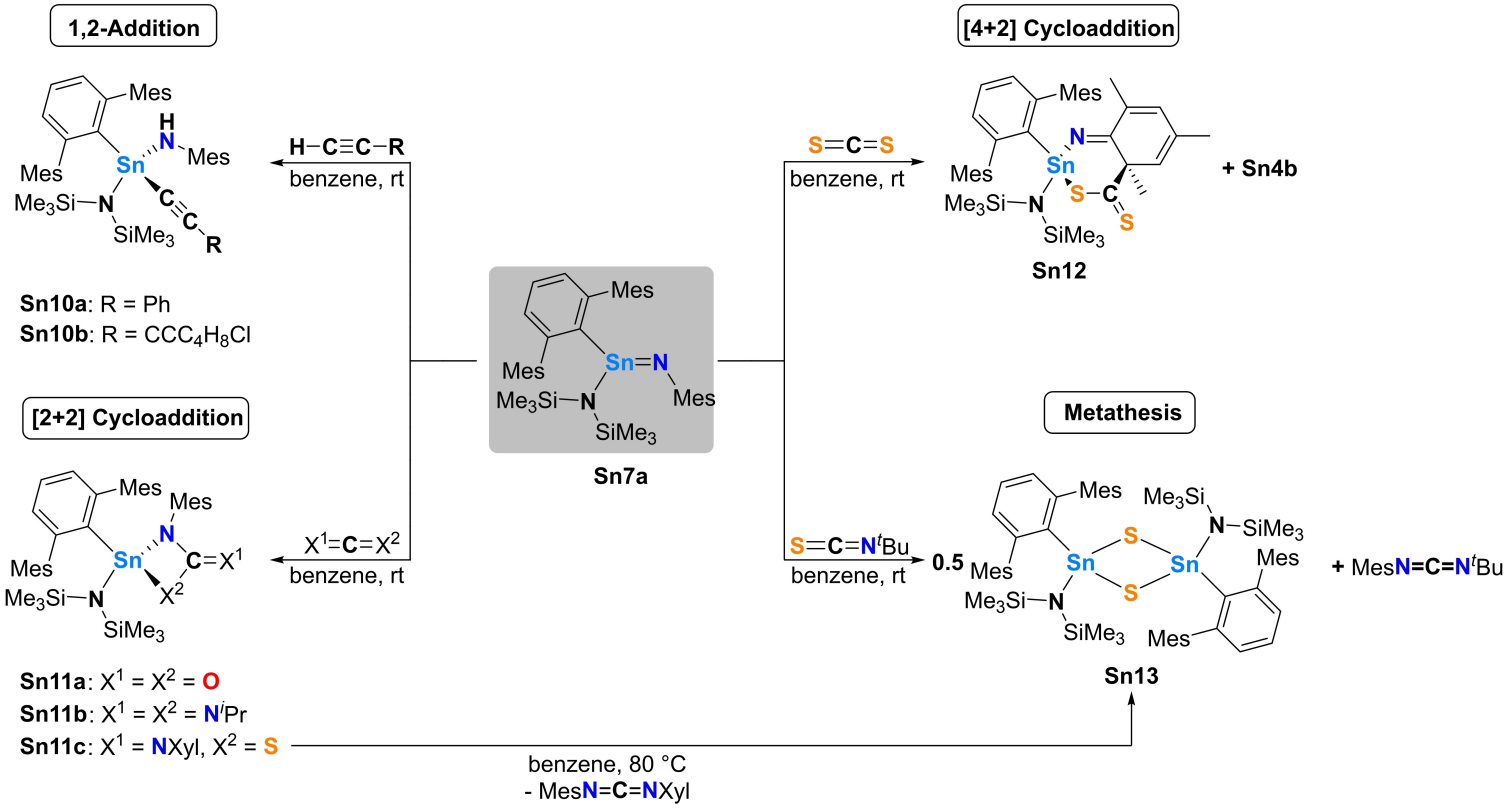
Reactivity of stannaimine **Sn7a**.

**Figure 9:**
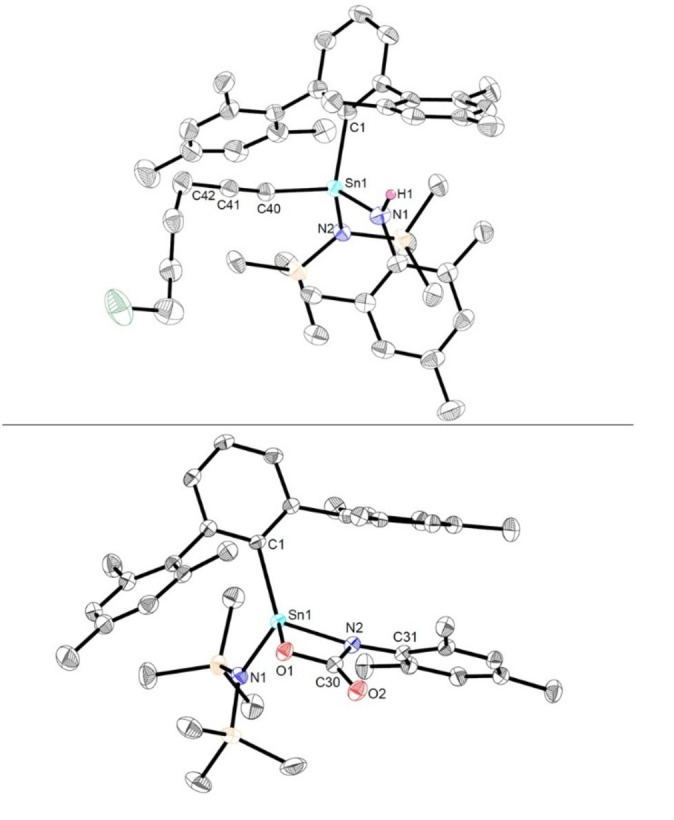
Top: Molecular structure of ^Mes^TerSn(N(SiMe_3_)_2_)(NHMes)CCC_4_H_8_Cl (**Sn10b**) in the crystal. Thermal ellipsoids are drawn at the 50 % probability level (hydrogen atoms (except H1) have been omitted for clarity). Selected bond lengths [Å] and angles [deg]: Sn1−N1 2.057(2), Sn1−N2 2.051(2), Sn1−C1 2.181(3), Sn1−C40 2.087(3), C40−C41 1.189(4), C41−C42 1.468(4), N1−Sn1−N2 106.37(9), N1−Sn1−C40 104.17(10); bottom: Molecular structure of the [2+2] cycloaddition product **Sn11a** in the crystal. Thermal ellipsoids are drawn at the 50 % probability level (hydrogen atoms have been omitted for clarity). Selected bond lengths [Å] and angles [deg]: Sn1−N1 2.021(2), Sn1−N2 2.0976(19), Sn1−O1 2.0361(16), Sn1−C1 2.140(2), O1−C30 1.355(3), N2−C30 1.390(3), O2−C30 1.214(3), N1−Sn1−N2 112.46(8), O1−Sn1−N2 65.10(7).

Interestingly, although the Dipp‐ and Quin‐substituted derivatives **Sn7b** and **Sn8** react with phenylacetylene, these reactions are slow and intramolecular C−H activation competes, so that only mixtures of products could be obtained (Figure S46). This emphasizes the fine balance needed to tune the system to render it isolable, but at the same time sufficiently reactive towards external substrates. By contrast, Mes‐substituted derivative **Sn7a** reacts rapidly and cleanly with phenylacetylene to give the corresponding 1,2‐addition product ^Mes^TerSn{N(SiMe_3_)_2_}(CCPh)(NHMes) (**Sn10a**) (Scheme 5), implying that this specific substitution pattern is an ideal compromise, allowing for both the isolation of the reactive tin nitrogen double bond *and* clean reactivity towards external substrates. The reduced steric profile of the Mes substituent (cf. Dipp) presumably allows faster kinetics in intermolecular reactivity. A similar result was obtained by employing an aliphatic terminal alkyne to give the corresponding C−H activation product ^Mes^TerSn(N(SiMe_3_)_2_)(CC_4_H_8_Cl)(NHMes) (**Sn10b**). **Sn10a** and **Sn10b** were both characterized by single crystal X‐ray diffraction (Figure 9 (top) and Figure S51). The regiochemistry of C−H addition in each case is consistent with the polarity of the Sn=N bond revealed computationally, and with (effectively) deprotonation of the terminal alkyne by the strongly basic imide nitrogen.

In the context of cycloaddition chemistry, a range of heteroallenes (CO_2_, ^*i*^PrNCN^*i*^Pr, CS_2_, SCNR (R=^*t*^Bu, Xyl)) was reacted with **Sn7a**. The addition of 1 bar of CO_2_ or one equivalent of *N*,*N*′‐diisopropylcarbodiimide results in an immediate color change of the solution to bright yellow. In each case ^1^H NMR analyses confirm the formation of a single product, namely the [2+2] cycloaddition products **Sn11a,b**, whose connectivity could be unambiguously verified by single crystal X‐ray diffraction (Figure 9 (bottom)). These complexes are the first examples of structurally characterized [2+2] cycloaddition products derived from stannaimines. Comparing their structures with those of related compounds derived from germaimines show that they are in good accordance (near planarity of the central four‐membered ring, exocyclic double bond character, and behavior as a dianionic ligand to the tetrel center).^[12]^ The general scalabilty of the synthesis of stannaimine **Sn7a** and compounds derived therefrom was demonstrated starting with 500 mg of **Sn1**. Since **Sn1**—although comparatively slowly—undergoes intramolecular C−H activation, we recommend to always synthesize it in situ, determine purity by ^1^H NMR spectroscopy of an aliquot and directly react it with the substrate. Accordingly, **Sn1** was converted to **Sn7a** and further reacted exemplarily with iPrNCNiPr to give 628 mg of **Sn11b** (which corresponds to an isolated yield of 87 %).^[7]^

**Figure 10:**
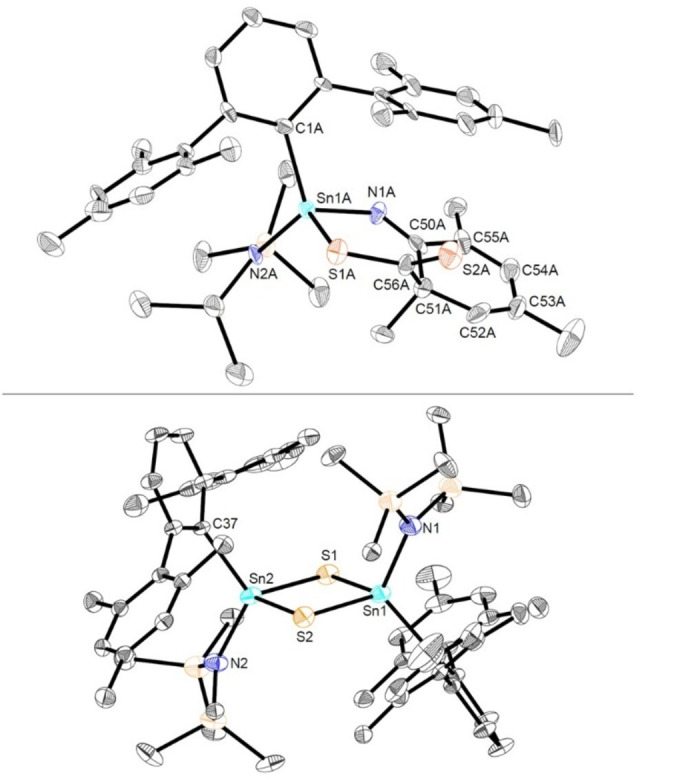
Top: Molecular structure of the [4+2] cycloaddition product **Sn12** in the crystal. Thermal ellipsoids are drawn at the 50 % probability level (hydrogen atoms have been omitted for clarity). Selected bond lengths [Å] and angles [deg]: Sn1A−N1A 2.036(11), Sn1A−N2A 2.058(9), Sn1A−C1A 2.152(11), Sn1A−S1A 2.438(3), S1A−C56A 1.775(13), S2A−C56A 1.649(14), N1A−C50A 1.269(17), C50A−C51A 1.559(19), C51A−C52A 1.502(18), C52A−C53A 1.31(2), C53A−C54A 1.48(2), C54A−C55A 1.37(2), C50A−C55A 1.483(18), C51A−C56A 1.503(19), N1A−Sn1A−S1A 98.9(3), N1A−Sn1A−C1A 110.7(4); bottom: Molecular structure of (^Mes^TerSn(N(SiMe_3_)_2_)S)_2_ (**Sn13**) in the crystal. Thermal ellipsoids are drawn at the 50 % probability level (hydrogen atoms have been omitted for clarity). Selected bond lengths [Å] and angles [deg]: Sn1−S1 2.4218(5), Sn1−S2 2.4218(5), Sn2−S1 2.4225(5), Sn2−S2 2.4204(4), Sn1−N1 2.0627(16), Sn1−C7 2.2010(18), N1−Sn1−C7 115.68(7), Sn1−S1−Sn2 87.965(15), S1−Sn1−S2 91.990(16).

Interestingly, the reaction of **Sn7a** with an excess of the heavier (but usually more reactive) CO_2_ analogue carbon disulfide is significantly slower, and therefore significant amounts of the competing C−H activation product **Sn4b** can be observed by in situ ^1^H NMR monitoring (Figure S66). Despite the formation of **Sn4b**, however, we succeeded in crystallizing the formal [4+2] cycloaddition product of **Sn7a** with CS_2_ (**Sn12**; Scheme 5). The molecular structure of **Sn12** reveals that cycloaddition occurs between one of the C=S double bonds of CS_2_ and the heterodiene motif (Sn=N−C(sp^2^)=C(sp^2^)), comprised of the tin‐nitrogen double bond and the adjacent (localized) carbon‐carbon double bond of the mesityl substituent. Comparable reactivity has been observed in reactions of a germanimine with carbonyl compounds and alkenes, respectively.^[12]^ Notably, although both the tin center and the ortho carbon atom of the dearomatized mesityl group are stereogenic centers, **Sn12** is formed diastereomerically pure as a mixture of the *R*,*S* and *S*,*R* enantiomers (as determined by single crystal X‐ray diffraction). The molecular structure of **Sn12** is shown in Figure 10 (top).

As a consequence of the dearomatization of the mesityl substituent, carbon‐carbon double bonds are localized between C52A and C53A and between C54A and C55A, respectively (1.31(2) Å and 1.37(2) Å). The exocyclic N1A−C50A bond length of 1.269(17) Å is typical of a double bond with all the other bond lengths within the stannacycle being characteristic of single bonds. The exocyclic C56A−S2A bond length of 1.649(14) Å is significantly shorter than the C56A−S1A bond length of 1.775(13) Å within the six‐membered ring system, consistent with a description of the former as a double bond.

Due to diverging reactivity of **Sn1** towards these heteroallenes—in particular towards ^*i*^PrNCN^*i*^Pr and CS_2_—the reactivity of the Sn=N double bond towards isothiocyanates (which contain both C=N and C=S functionalities) was viewed as being of significant interest. Accordingly, the reaction of the aryl‐substituted isothiocyanate SCNXyl with **Sn1** was investigated under comparable conditions. In this case, the reaction can be shown by a combination of spectroscopic and crystallographic methods to lead to the selective formation of the [2+2] cyclo‐addition product **Sn11c** (Scheme 5). The regioselectivity of this transformation was unambiguously confirmed by single crystal X‐ray diffraction, showing that cycloaddition has occurred between the Sn=N double bond of the stannaimine and the weaker C=S double bond of the isothiocyanate (Figure S74). Interestingly, the reaction of **Sn1** with the aliphatic isothiocyanate SCN^*t*^Bu at room temperature results in clean formation of the sulfur‐bridged dimer {^Mes^TerSn(N(SiMe_3_)_2_)S}_2_ (**Sn13**), as verified by single crystal X‐ray diffraction (Figure 10 (bottom)), together with the carbodiimide MesN=C=N^*t*^Bu, implying that an overall metathesis process has occurred, with accompanying dimerization of the stannathione. The formation of **Sn13** could also be achieved starting from **Sn11c** by heating a benzene solution of the *Sn*,*N*,*C*,*S‐*containing metallacycle at 80 °C for one hour, or by prolonged storage at room temperature (cf. Figure S73). In this case trapping of the intermediate **Sn11c** which links stannaimine **Sn7a** (plus isothiocyanate) with thiostannone **Sn13** (plus carbodiimide) implies that the metathesis process occurs via a similar mechanism to more well‐known d‐block systems.

## Conclusions

We have shown that stannaimines can be stabilized systematically by combining an organoazide with a heteroleptic stannylene featuring one strong σ donor (terphenyl) and one π donor substituent (N(SiMe_3_)_2_). This allows for systems which can (uniquely) be handled under ambient conditions. It was further shown that, the substituent at the stannaimine nitrogen functionality is crucial for allowing subsequent reactivity. This detailed study allowed for the realisation of 1,2‐addition reactions, [2+2] and [4+2] cycloaddition reactions and a very rare example of main group double bond metathesis chemistry at a stannaimine template. By contrast, using heteroleptic stannylenes with two strong σ donor ligands (terphenyl/hypersilyl) in reactions with organic azides seem to give the putative tin nitrogen double bond but subsequent silyl migration is favoured significantly, so that a series of novel heteroleptic stannylenes with terphenyl and bulky amido ligands are formed.^[13]^

## Conflict of interest

The authors declare no conflict of interest.

1

## Supporting information

As a service to our authors and readers, this journal provides supporting information supplied by the authors. Such materials are peer reviewed and may be re‐organized for online delivery, but are not copy‐edited or typeset. Technical support issues arising from supporting information (other than missing files) should be addressed to the authors.

Supporting Information

Supporting Information

Supporting Information

Supporting Information

Supporting Information

Supporting Information

Supporting Information

Supporting Information

Supporting Information

Supporting Information

Supporting Information

Supporting Information

Supporting Information

Supporting Information

Supporting Information

Supporting Information

Supporting Information

Supporting Information

Supporting Information

Supporting Information

Supporting Information

Supporting Information

## Data Availability

Data in support of this publication is publicly available through the Oxford Research Archive (ORA).
