# Supplementary material for: Taming Heavier Group 14 Imine Analogues: Accessing Tin Nitrogen [Sn=N] Double Bonds and their Cycloaddition/Metathesis Chemistry
Source: Angew Chem Int Ed Engl. 2022 Oct 25;61(48):e202211616. doi: 10.1002/anie.202211616 (PMC9828258; doi:10.1002/anie.202211616)
Supplement: Supplementary file 1 — Supporting Information [file ANIE-61-0-s012.pdf]

Angewandte Chemie
Eine Zeitschrift der Gesellschaft Deutscher Chemiker

Supporting Information

Taming Heavier Group 14 Imine Analogues: Accessing Tin Nitrogen [Sn=N] Double Bonds and their Cycloaddition/Metathesis Chemistry

M. Fischer, M. M. D. Roy, L. L. Wales, M. A. Ellwanger, C. McManus, A. F. Roper, A. Heilmann, S. Aldridge**

Table of Contents

**General Considerations**	**S2**
**Synthesis and Characterization of Compounds**	**S3**
**Crystallographic Details**	**S65**
**Computational Details**	**S72**
**References**	**S76**

General Considerations

All manipulations were carried out using standard Schlenk line and glove box techniques under an atmosphere of dry argon or dinitrogen. Solvents were degassed by sparging with argon and dried by passing through a column of appropriate drying agent using a commercially available Braun SPS and stored over potassium mirror. $^{Mes}TerSn(N(SiMe_3)_2$ (**Sn1**),[S1] $(thf)_2K(Si(SiMe_3)_3)$,[S2] N_3Mes (**A2**),[S3] N_3Dipp (**A3**),[S3] $N_3{}^{Mes}Ter$ (**A4**)[S4] and N_3Quin (**A5**)[S5] were synthesized according to literature procedures. N_3SiMe_3 (**A1**), phenylacetylene, 6-chlor-hex-1-yne, N,N'-diisopropylcarbodiimide, carbon disulfide, 2,6-dimethylphenyl isothiocyanate and *tert*-butyl isothiocyanate were freeze-pump-thaw degassed and stored over molecular sieves under inert atmosphere prior to use. NMR spectra were measured in benzene-d_6 (C_6D_6) which was dried over CaH_2, with the solvent being distilled under reduced pressure, degassed by three freeze-pump-thaw-cycles and stored under argon in a Teflon valve ampoule. NMR samples were prepared under argon in 5 mm Wilmad 507-PP tubes fitted with J. Young Telfon valves. NMR spectra were measured on a Bruker Avance III HD Nanobay 400 MHz NMR spectrometer equipped with a 9.4 T magnet, Bruker Avance III 500 MHz NMR spectrometer equipped with a 11.75 T magnet or a Bruker Avance III NMR 500 MHz NMR spectrometer equipped with a 11.75 T magnet and a ^{13}C detect cryoprobe. ^1H and ^{13}C NMR spectra were referenced internally to residual protio-solvent (^1H) or solvent (^{13}C) resonances and are reported relative to tetramethylsilane (δ = 0 ppm). $^{29}Si\{^1H\}$ NMR spectra are referenced externally to tetramethylsilane. $^{119}Sn\{^1H\}$ NMR spectra were referenced with respect to $SnMe_4$ in C_6D_6. Chemical shifts are quoted in δ (ppm) and coupling constants in Hz. The reported yields are the yields obtained after crystallisation and subsequent measurement of the material by single crystal-ray diffraction if not stated otherwise. UV-VIS spectra were recorded on a Horiba Duetta spectrofluorometer at 298K. Elemental analyses were carried out by London Metropolitan University.

Synthesis and Characterization of Compounds

Synthesis of ^{Mes}TerSnSi(SiMe$_3$)$_3$ (Sn2)

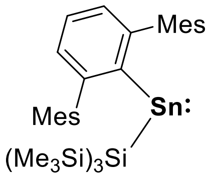

^{Mes}TerSnN(SiMe$_3$)$_2$ (**Sn1**) (0.500 g, 0.844 mmol) and KSi(SiMe$_3$)$_3$ (0.242 g, 0.844 mmol) were dissolved in ca. 30 mL of toluene. The reaction mixture was stirred for 16 h at room temperature which results in a colour change from orange-red to dark green accompanied by precipitation of a colourless solid. The solution is collected by filtration and concentrated to incipient crystallization. Storage of the solution at -30 °C yields ^{Mes}TerSnSi(SiMe$_3$)$_3$ (**Sn2**) as a dark blue crystalline solid. These crystals were suitable for single crystal X-ray diffraction. A second crop of crystals can be obtained from the concentrated mother liquor.

Yield: 0.497 g (0.731 mmol; 87%).
^{1}H NMR (400 MHz, C_6D_6, 298 K): δ = 0.21 (s, 27H, Si(CH$_3$)$_3$), 2.11 (s, 6H, CH$_3$), 2.37-2.41 (m, 12H, CH$_3$), 6.64-6.65 (m, 2H, CH$_{Aryl}$), 6.82-6.83 (m, 2H, CH$_{Aryl}$), 7.04-7.06 (m, 2H, CH$_{Aryl}$), 7.35-7.37 (m, 1H, CH$_{Aryl}$) ppm.
^{13}C{^{1}H} NMR (101 MHz, C_6D_6, 298 K): δ = 4.7 (Si(CH$_3$)$_3$), 21.2 (CH$_3$), 22.2 (CH$_3$), 128.7 (CH$_{Aryl}$), 129.5 (CH$_{Aryl}$), 129.6 (CH$_{Aryl}$), 130.2 (CH$_{Aryl}$), 136.2 (C$_{q,Aryl}$), 136.4 (C$_{q,Aryl}$), 137.8 (C$_{q,Aryl}$), 146.0 (C$_{q,Aryl}$), 179.2 (C$_{q,Aryk}$Sn) ppm.
^{29}Si{^{1}H} NMR (80 MHz, C_6D_6, 298 K): δ = -39.7, -4.5 ppm. (assigned by ^{1}H/^{29}Si HMBC)
^{119}Sn{^{1}H} NMR (149 MHz, C_6D_6, 298 K): not observed.
EA: Anal. calcd. for $C_{33}H_{52}Si_4Sn$: C, 58.30; H, 7.71; Found: C, 57.81; H, 7.82.

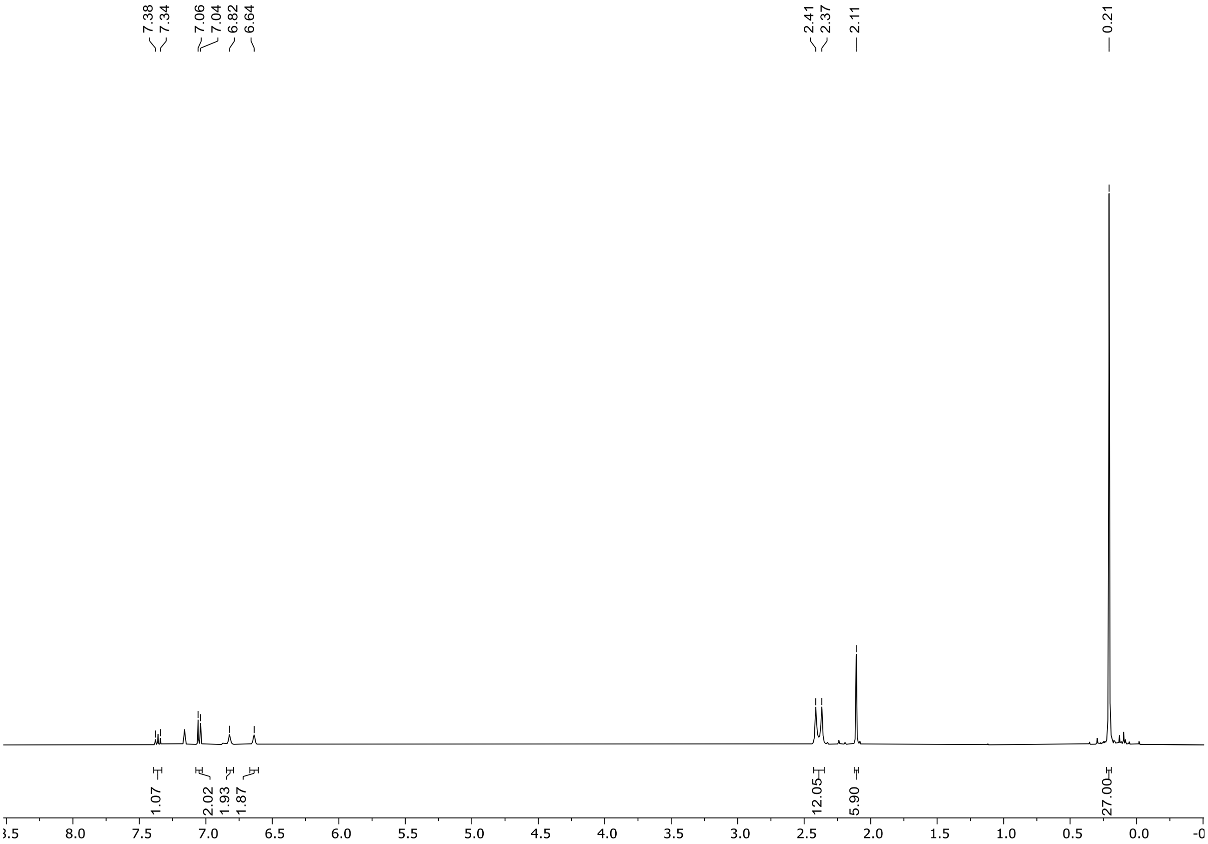

Figure S1. ^{1}H NMR spectrum of ^{Mes}TerSnSi(SiMe$_3$)$_3$ (**Sn2**) (400 MHz, C_6D_6, 298 K).

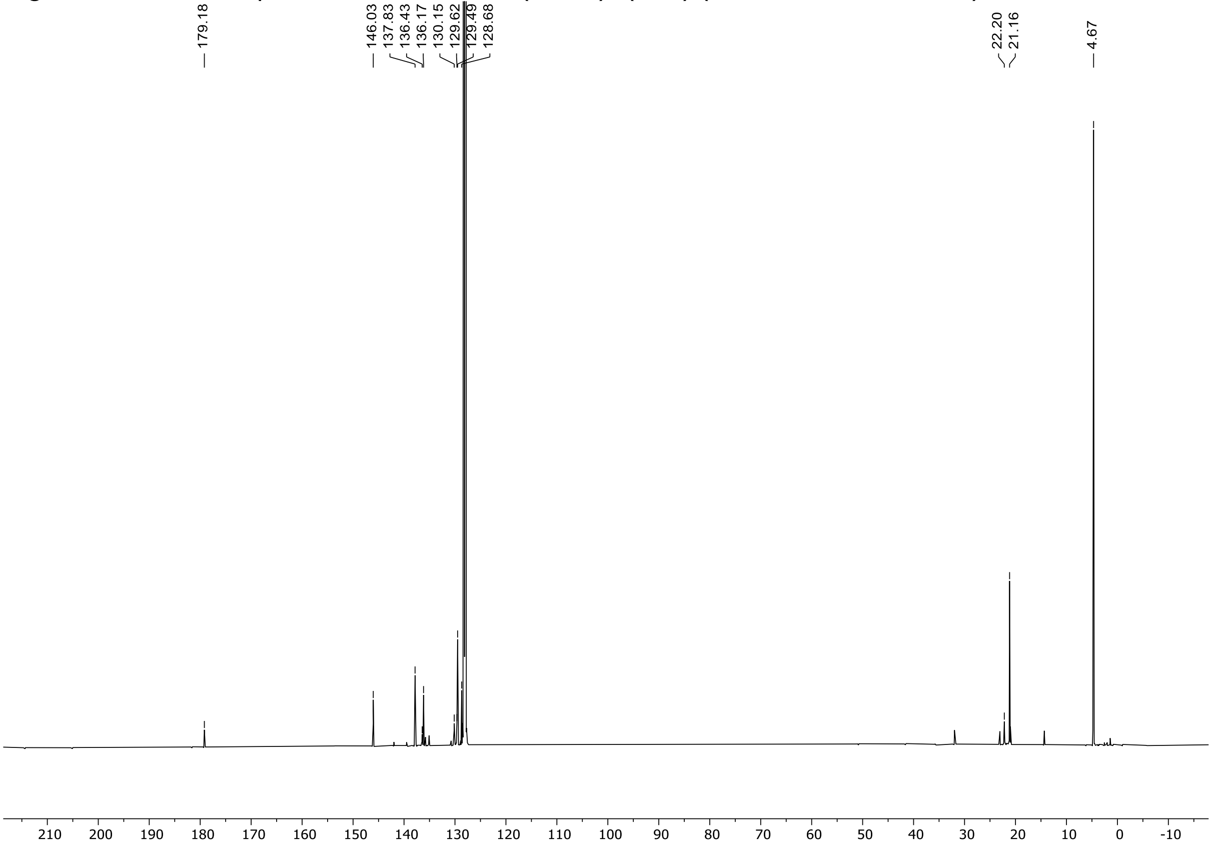

Figure S2: $^{13}C\{^{1}H\}$ NMR spectrum of ^{Mes}TerSnSi(SiMe$_3$)$_3$ (**Sn2**) (101 MHz, C_6D_6, 298 K).

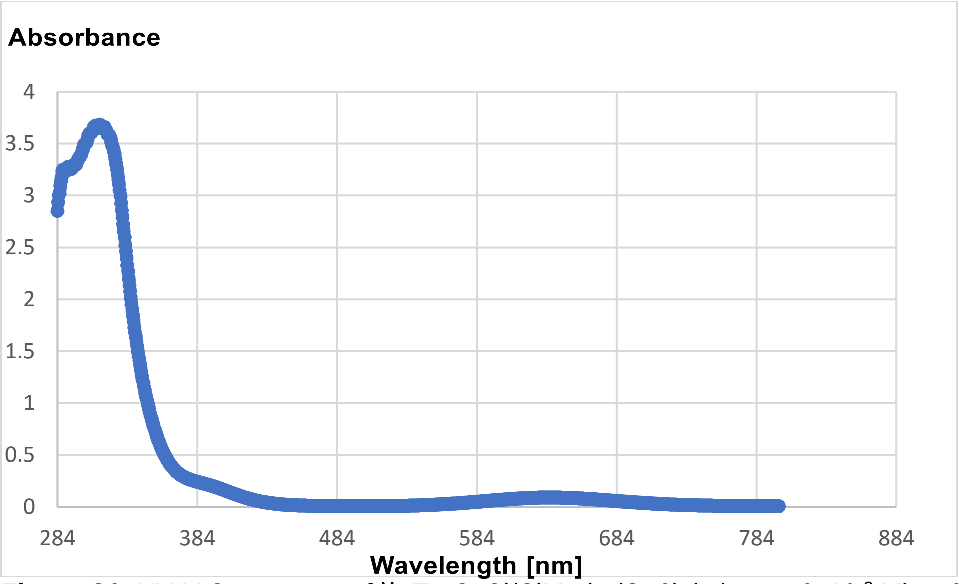

Figure S3. UV-VIS spectrum of $^{Mes}TerSnSi(SiMe_3)_3$ (**Sn2**) (toluene, $2x10^{-3}$ M) at 298 K.

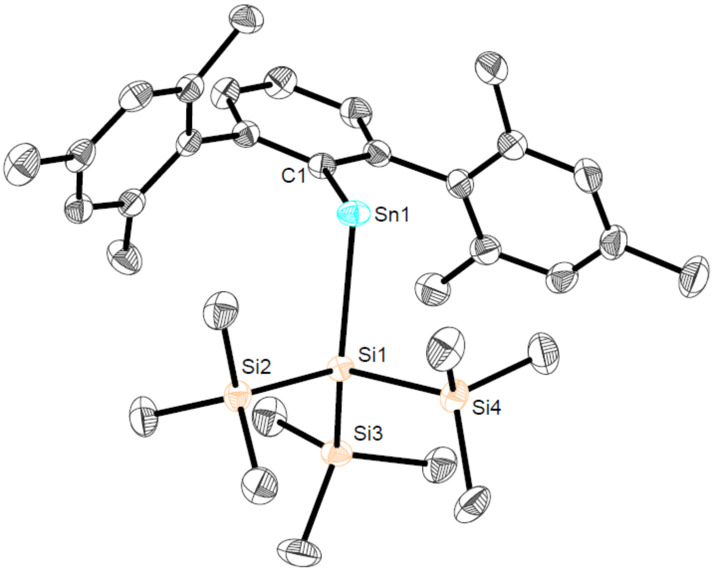

Figure S4. Molecular structure of $^{Mes}TerSnSi(SiMe_3)_3$ (**Sn2**) in the crystal. Thermal ellipsoids are drawn at the 50% probability level (hydrogen atoms have been omitted for clarity). Selected bond lengths (Å) and angles (deg): Sn1–Si1 2.6407(7), Sn1–C1 2.189(3), C1–Sn1–Si1 109.75(6).

Reaction of ^{Mes}TerSnSi(SiMe$_3$)$_3$ (Sn2) with N$_3$SiMe$_3$ (A1)

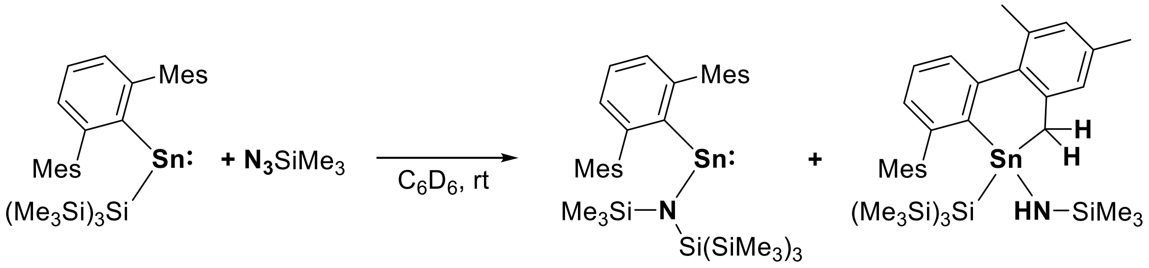

To a suspension of ^{Mes}TerSnSi(SiMe$_3$)$_3$ (**Sn2**) (0.030 g, 0.044 mmol) in 0.3 mL of C_6D_6 was added a solution of N$_3$SiMe$_3$ (**A1**) (0.005 g, 0.044 mmol) in 0.3 mL of C_6D_6 which results in immediate gas evolution and a colour change to yellow-orange. Subsequent analysis of the NMR data revealed that two products are formed in an approximate ratio of 1.5:1.0 (determined by ^{1}H NMR spectroscopy (Figure S6)). Repeating the reaction at -30 °C lead to the same outcome. Crystals of the minor component suitable for single crystal X-ray diffraction were identified to be the intramolecular C(sp^3)–H activation product **Sn4a**. These crystals were obtained from a saturated *n*-hexane solution at -30 °C. Crystals of the major component suitable for single crystal X-ray diffraction were obtained by keeping a saturated *n*-hexane solution for approximately one month at -30 °C which lead to co-crystallisation of **Sn4a** and the stannylene ^{Mes}TerSnN(SiMe$_3$)Si(SiMe$_3$)$_3$ (**Sn3a**).

Analytical data of C–H-activation product Sn4a:
Yield: 0.011 g (0.014 mmol; 33%).
^{1}H NMR (400 MHz, C_6D_6, 298 K): δ = -0.99 (s, 1H, NH), 0.16 (s, 36H, Si(CH$_3$)$_3$), 2.15 (s, 3H, CH$_3$), 2.22 (s, 3H, CH$_3$), 2.23 (s, 3H, CH$_3$), 2.27 (s, 3H, CH$_3$), 2.30 (s, 3H, CH$_3$), 2.59 (d, $^{2}J_{H,H}$ = 11.2 Hz, Sn satellites: $^{2}J_{119Sn,H}$ = 36.7 Hz, CH$_2$), 2.88 (d, $^{2}J_{H,H}$ = 11.2 Hz, Sn satellites: $^{2}J_{119Sn,H}$ = 93.8 Hz, $^{2}J_{117Sn,H}$ = 71.7 Hz, CH$_2$), 6.85-6.86 (m, 3H, CH$_{Aryl}$), 6.88-6.90 (m, 2H, CH$_{Aryl}$), 7.20-7.23 (m, 2H, CH$_{Aryl}$) ppm.
^{13}C{^{1}H} NMR (101 MHz, C_6D_6, 298 K): δ = 3.1 (Si(Si(CH$_3$)$_3$)$_3$), 4.3 (HNSi(CH$_3$)$_3$), 21.09 (CH$_3$), 21.13 (CH$_3$), 22.8 (CH$_3$), 23.1 (CH$_3$), 23.2 (CH$_3$), 27.3 (Sn satellites: $^{1}J_{119Sn,C}$ = 250.7 Hz, $^{1}J_{117Sn,C}$ = 239.5 Hz, CH$_2$), *, 128.45 (CH$_{Aryl}$), 128.54 (CH$_{Aryl}$), 129.4 (CH$_{Aryl}$), 130.1 (CH$_{Aryl}$), 130.6 (CH$_{Aryl}$), 135.6 (C$_{q,Aryl}$), 136.6 (C$_{q,Aryl}$), 136.7 (C$_{q,Aryl}$), 136.8 (C$_{q,Aryl}$), 137.4 (C$_{q,Aryl}$), 138.9 (C$_{q,Aryl}$), 141.9 (C$_{q,Aryl}$), 145.9 (C$_{q,Aryl}$), 147.4 (C$_{q,Aryl}$), 149.8 (C$_{q,Aryl}$) ppm.
* = two CH$_{Aryl}$ overlapped by C_6D_6 signal
^{29}Si{^{1}H} NMR (80 MHz, C_6D_6, 298 K): δ = 3.4, -7.4, -121.4 ppm. (assigned by ^{1}H/^{29}Si HMBC)
^{119}Sn{^{1}H} NMR (149 MHz, C_6D_6, 298 K): δ = -103.6 ppm.
EA: Anal. calcd. for $C_{36}H_{61}NSi_5Sn$: C, 56.37; H, 8.02; N, 1.83; Found: C, 55.80; H, 7.42; N, 1.61.

Characteristic NMR data of the stannylene Sn3a:
^{1}H NMR (400 MHz, C_6D_6, 298 K): δ = 0.13 (s, 9H, HNSi(CH$_3$)$_3$), 0.22 (s, 27H, Si(CH$_3$)$_3$), 2.18 (s, 6H, CH$_3$), 2.31 (s, 12H, CH$_3$), 6.79-6.80 (m, 2H, CH$_{Aryl}$), 7.075-7.07 (m, 2H, CH$_{Aryl}$), 7.23/7.27 (m, 1H, CH$_{Aryl}$) ppm.
^{13}C{^{1}H} NMR (101 MHz, C_6D_6, 298 K): δ = 3.2 (Si(Si(CH$_3$)$_3$)$_3$), 6.9 (Si(CH$_3$)$_3$), 21.1 (CH$_3$), 22.0 (CH$_3$), 130.2 (CH$_{Aryl}$), 130.26 (CH$_{Aryl}$), 130.32 (CH$_{Aryl}$), 134.8 (C$_{q,Aryl}$), 136.7 (C$_{q,Aryl}$), 138.4 (C$_{q,Aryl}$), 145.8 (C$_{q,Aryl}$), 187.1 (C$_{q,Aryl}$) ppm.
^{29}Si{^{1}H} NMR (80 MHz, C_6D_6, 298 K): δ = 5.5, -16.4, -38.3 ppm. (assigned by ^{1}H/^{29}Si HMBC)
^{119}Sn{^{1}H} NMR (149 MHz, C_6D_6, 298 K): not observed.
These data were obtained from the NMR spectra of the product mixture of **Sn4a** and **Sn3a**.

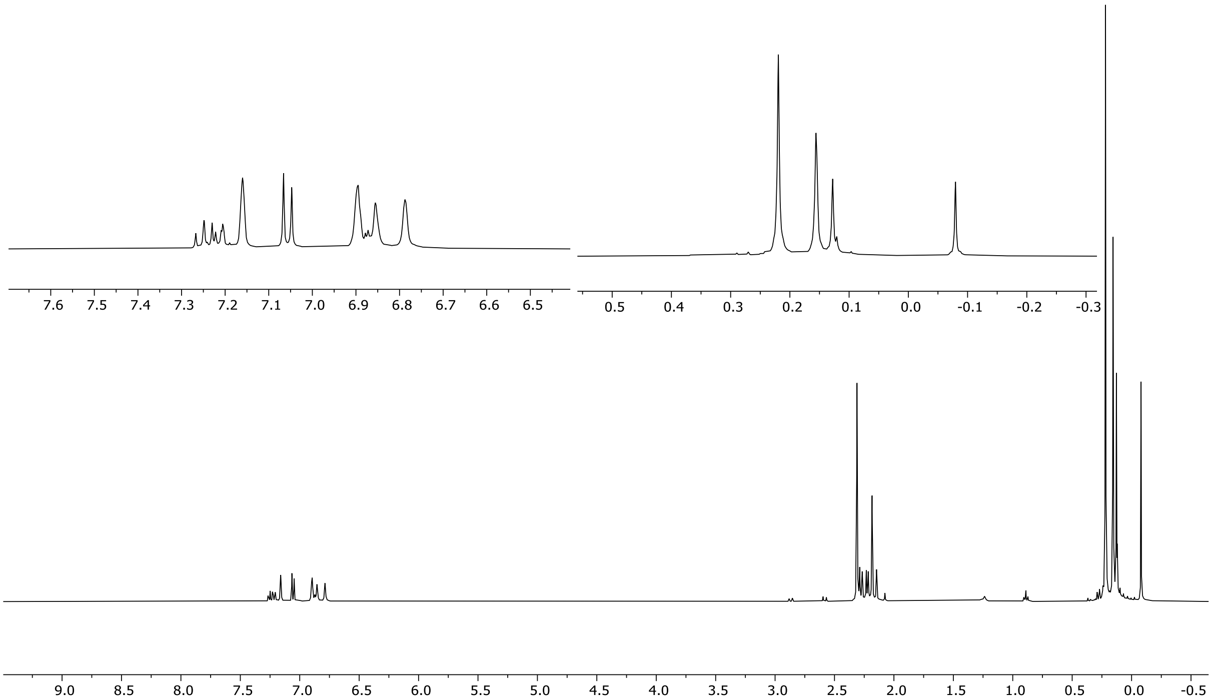

Figure S5. ^{1}H NMR spectrum after the reaction of ^{Mes}TerSnSi(SiMe$_3$)$_3$ (**Sn2**) with N$_3$SiMe$_3$ (**A1**) (400 MHz, C$_6$D$_6$, 298 K).

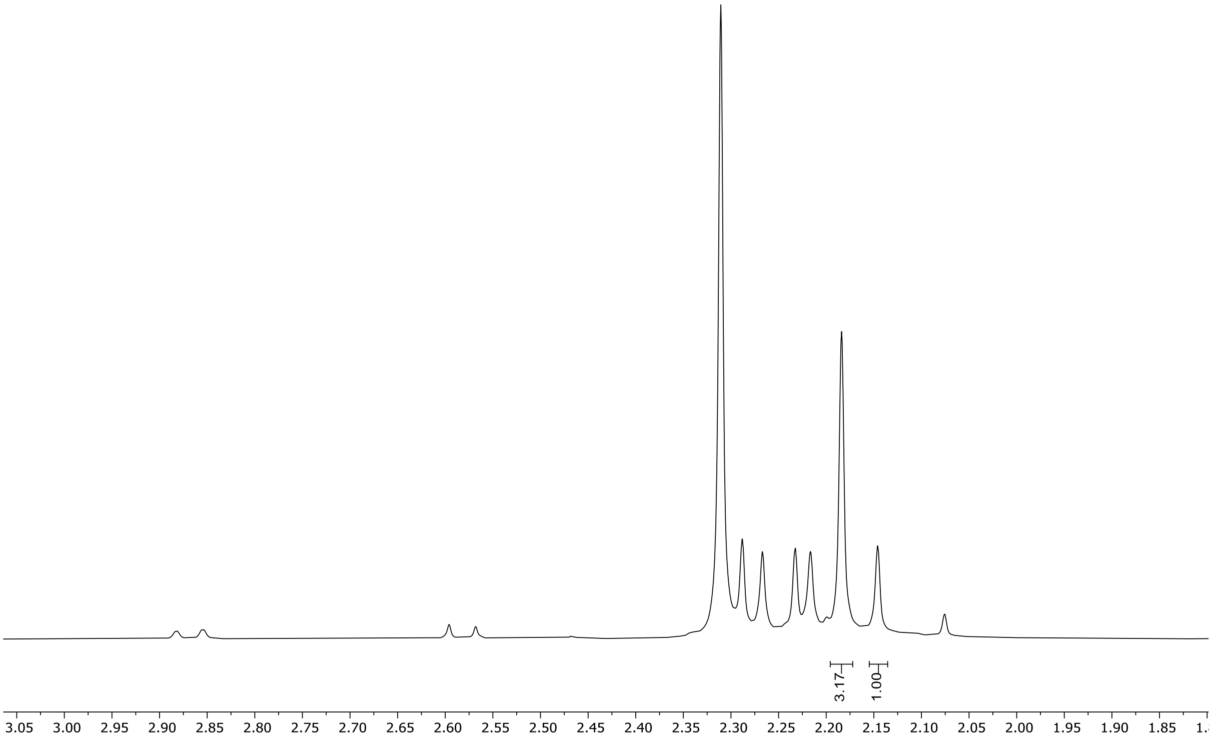

Figure S6. Excerpt of the ^{1}H NMR spectrum after the reaction of ^{Mes}TerSnSi(SiMe$_3$)$_3$ (**Sn2**) with N$_3$SiMe$_3$ (**A1**) to determine the ratio between **Sn3a** and **Sn4a** (400 MHz, C$_6$D$_6$, 298 K).

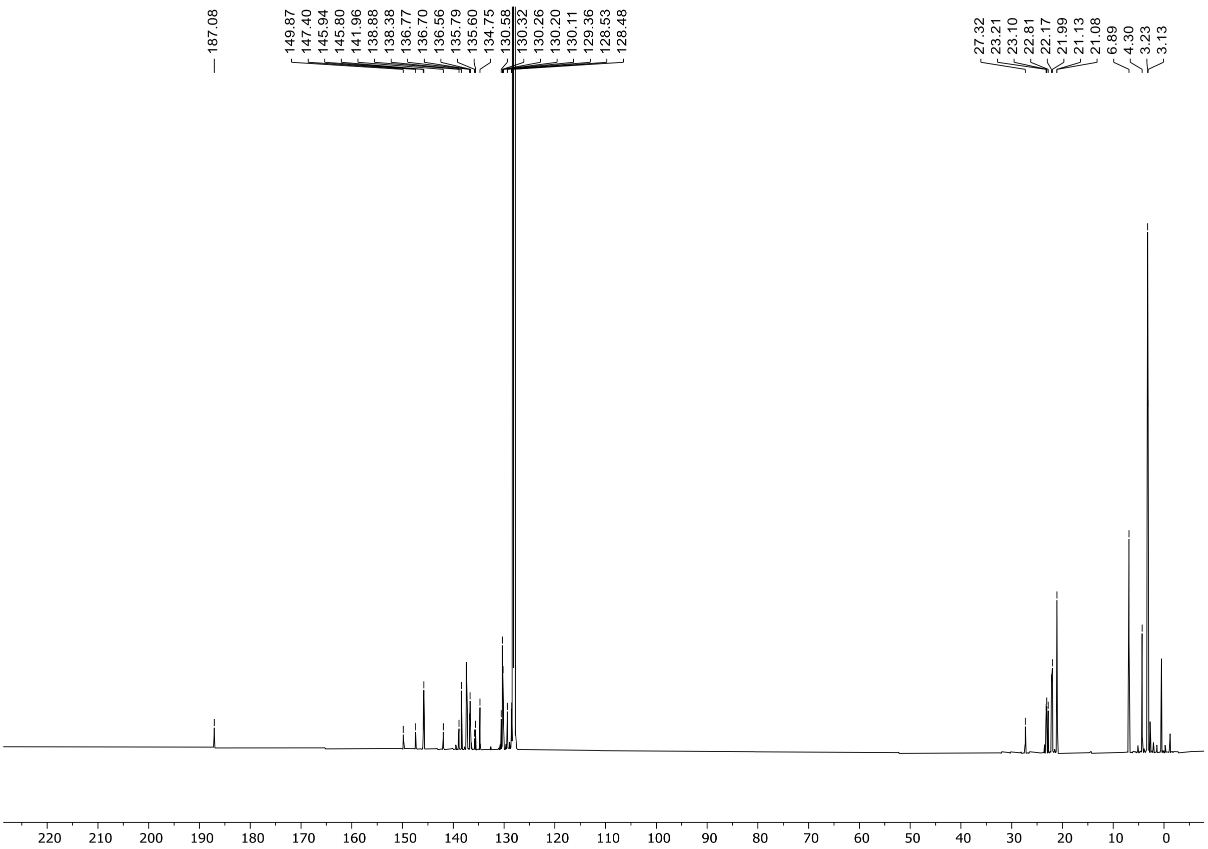

Figure S7: $^{13}C\{^{1}H\}$ NMR spectrum of the mixture of C–H-activation product **Sn4a** and stannylene **Sn3a** (101 MHz, C_6D_6, 298 K).

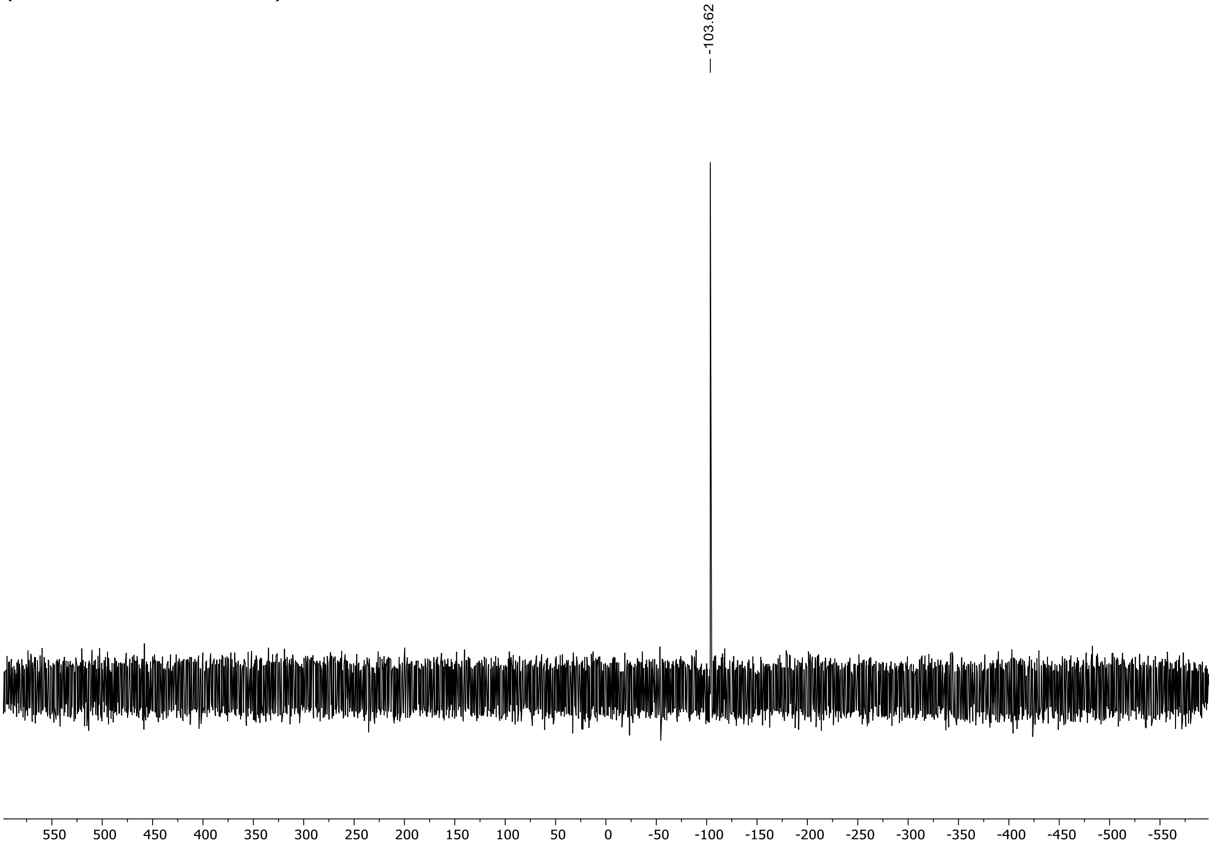

Figure S8. $^{119}Sn\{^{1}H\}$ NMR spectrum after the reaction of $^{Mes}TerSnSi(SiMe_3)_3$ (**Sn2**) with N_3SiMe_3 (**A1**) (149 MHz, C_6D_6, 298 K).

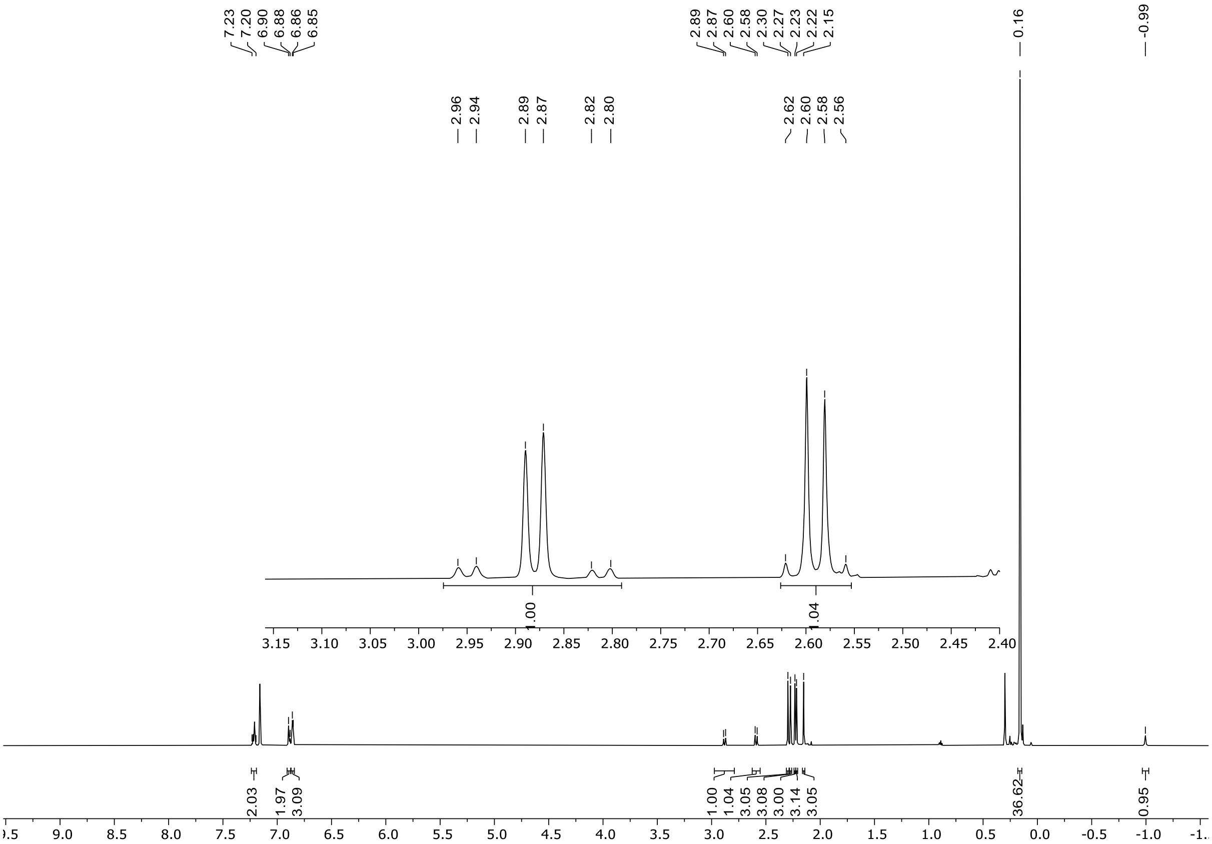

Figure S9. ^{1}H NMR spectrum of the C–H-activation product **Sn4a** (400 MHz, C_6D_6, 298 K).

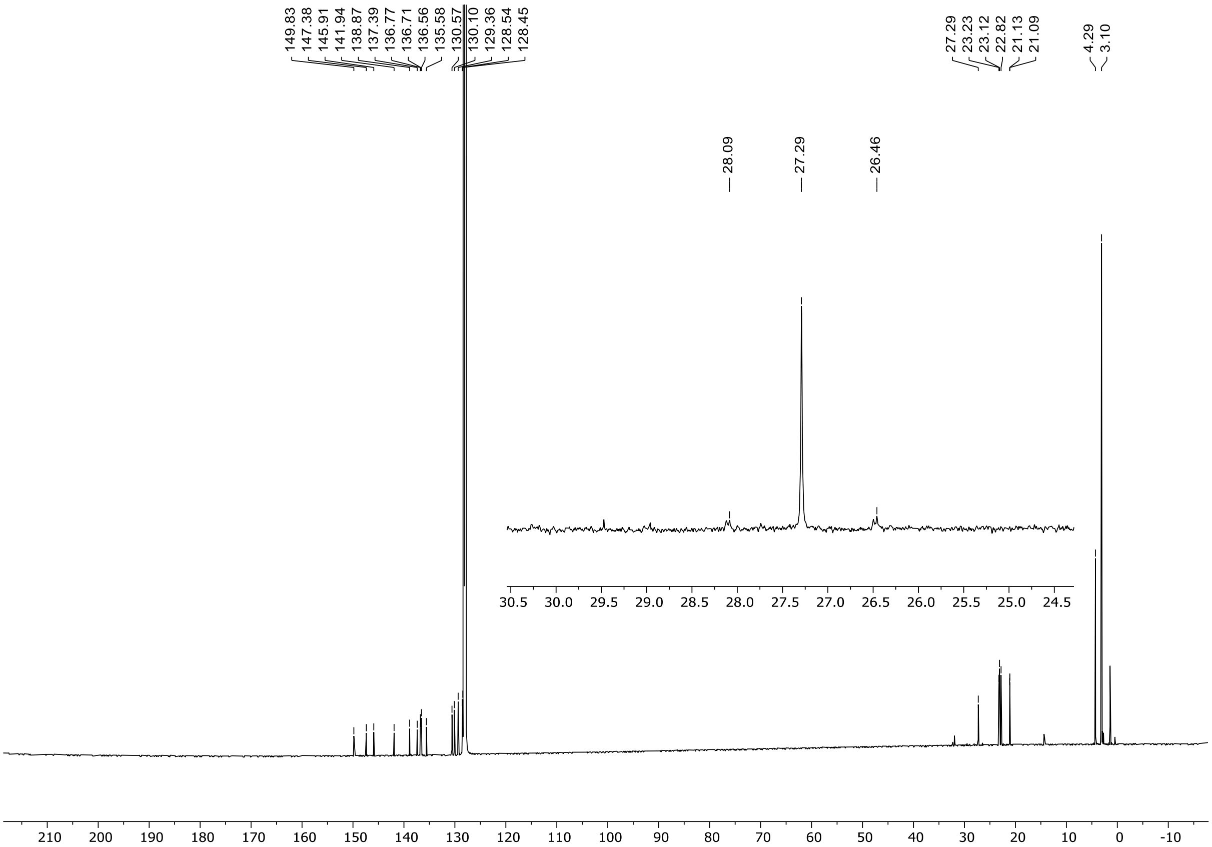

Figure S10: $^{13}C\{^{1}H\}$ NMR spectrum of C–H-activation product **Sn4a** (101 MHz, C_6D_6, 298 K).

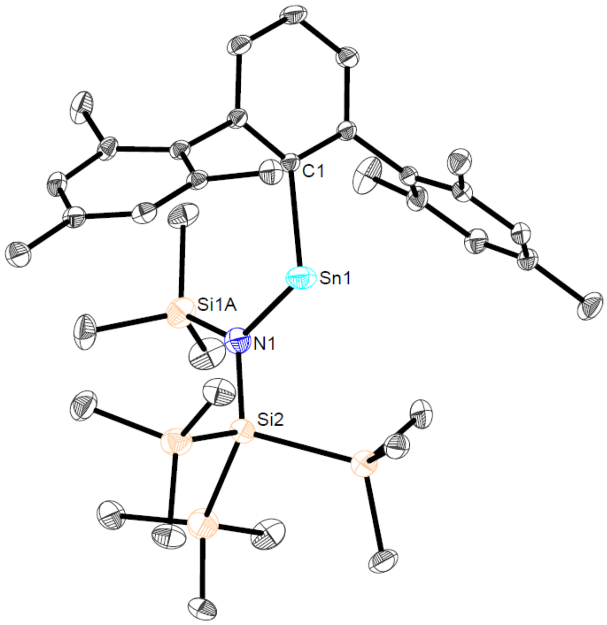

Figure S11. Molecular structure of [Mes]TerSnN(SiMe$_3$)Si(SiMe$_3$)$_3$ (**Sn3a**) in the crystal. Thermal ellipsoids are drawn at the 50% probability level (hydrogen atoms have been omitted for clarity). Selected bond lengths (Å) and angles (deg): Sn1–N1 2.0923(18), Sn1–C1 2.221(2), N1–Si1A 1.745(2), N1–Si2 1.7865(19), C1–Sn1–N1 111.23(8).

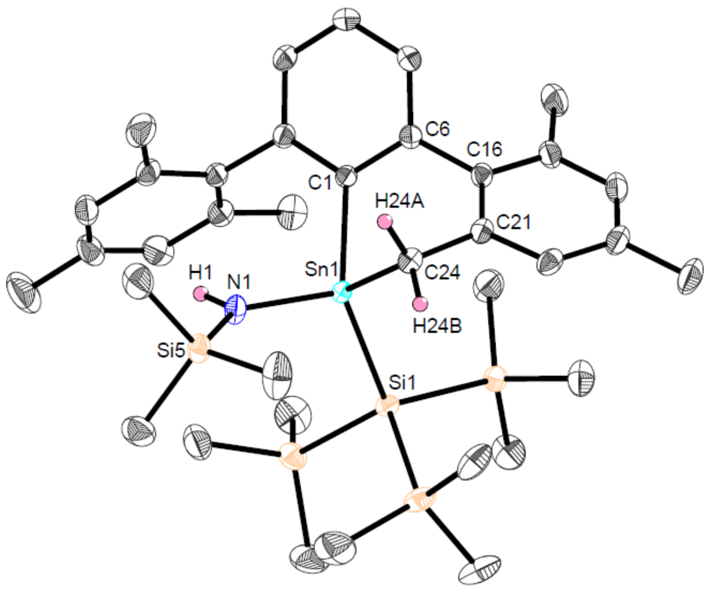

Figure S12. Molecular structure of the C–H activation product **Sn4a** in the crystal. Thermal ellipsoids are drawn at the 50% probability level (hydrogen atoms except for H1, H24A and H24B have been omitted for clarity). Selected bond lengths (Å) and angles (deg): Sn1–N1 2.0445(14), Sn1–C1 2.1754(16), Sn1–C24 2.1715(16), Sn1–Si1 2.5915(4), C21–C24 1.500(2), C1–Sn1–Si1 125.04(4), C1–Sn1–N1 106.37(6).

Synthesis of ^{Mes}TerSnN(Mes)Si(SiMe$_3$)$_3$ (Sn3b)

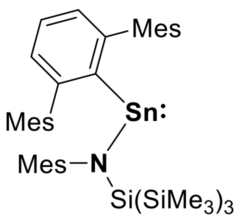

To a solution of ^{Mes}TerSnSi(SiMe$_3$)$_3$ (**Sn2**) (0.030 g, 0.044 mmol) in 0.3 mL of C_6D_6 was added a solution of N$_3$Mes (**A2**) (0.007 g, 0.044 mmol) in 0.3 mL of C_6D_6 which results in immediate gas evolution and a colour change to orange. All volatile components were removed under vacuum and the residue was dissolved in 1 mL of *n*-hexane, filtered and either stored at -30 °C to give ^{Mes}TerSnN(Mes)Si(SiMe$_3$)$_3$ (**Sn3b**) as an orange crystalline solid or followed by removal of all volatile components to give **Sn3b** as a powder. The supernatant was removed via syringe and the crystals dried under vacuum. Crystals obtained this way were suitable for single crystal X-ray diffraction.

Yield: 0.021 g (0.026 mmol; 59%).
^{1}H NMR (400 MHz, C_6D_6, 298 K): δ = 0.13 (s, 27H, Si(CH$_3$)$_3$), 1.83 (s, 6H, CH$_3$), 2.07 (s(br), 12H, CH$_3$), 2.21 (s, 3H, CH$_3$), 2.24 (s, 6H, CH$_3$), 6.73 (s, 2H, CH$_{Aryl}$), 6.84-6.86 (m, 6H, CH$_{Aryl}$), 7.09-7.13 (m, 1H, CH$_{Aryl}$) ppm.
^{13}C{^{1}H} NMR (101 MHz, C_6D_6, 298 K): δ = 2.4 (Si(CH$_3$)$_3$), 20.8 (CH$_3$), 21.2 (CH$_3$), 22.0 (CH$_3$), 22.7 (CH$_3$), 127.9 (CH$_{Aryl}$)*, 128.2 (CH$_{Aryl}$)*, 128.5 (CH$_{Aryl}$), 129.9 (CH$_{Aryl}$), 130.1 (C$_{q,Aryl}$), 131.6 (C$_{q,Aryl}$), 132.7 (C$_{q,Aryl}$), 137.5 (C$_{q,Aryl}$), 139.1 (C$_{q,Aryl}$), 146.9 (C$_{q,Aryl}$), 154.0 (C$_{q,Aryl}$), 181.2 (C$_{q,Aryl}$Sn) ppm.
* = overlap with C_6D_6 signal (assigned by ^{1}H/^{13}C HSCQC)
^{29}Si{^{1}H} NMR (80 MHz, C_6D_6, 298 K): δ = -30.5, -15.9 ppm. (assigned by ^{1}H/^{29}Si HMBC)
^{119}Sn{^{1}H} NMR (149 MHz, C_6D_6, 298 K): δ = -104.8 ppm. (assigned by ^{1}H/^{119}Sn HMBC)
EA: Anal. calcd. for $C_{42}H_{63}NSi_4Sn$: C, 62.05; H, 7.81; N, 1.72; Found: C, 62.06; H, 8.13; N, 1.93.

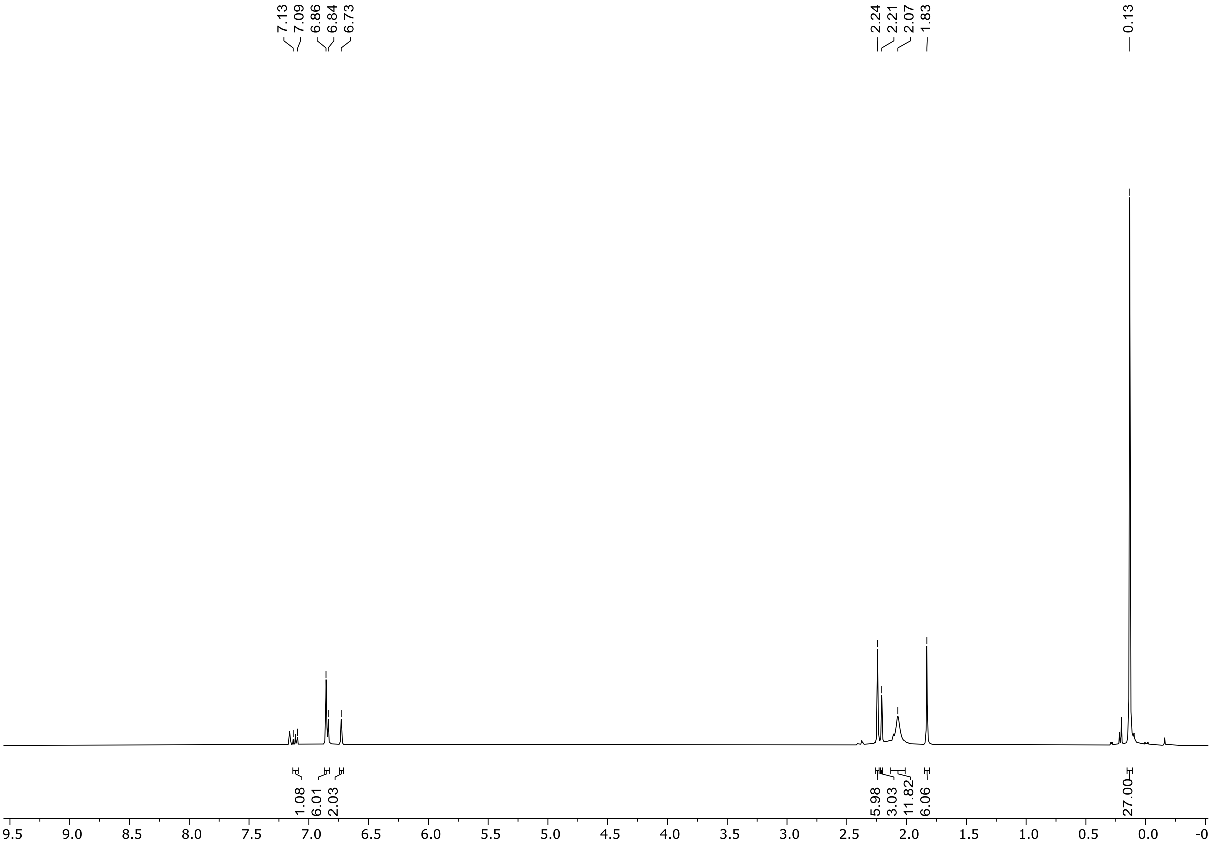

Figure S13. ^{1}H NMR spectrum of ^{Mes}TerSnN(Mes)Si(SiMe$_3$)$_3$ (**Sn3b**) (400 MHz, C_6D_6, 298 K).

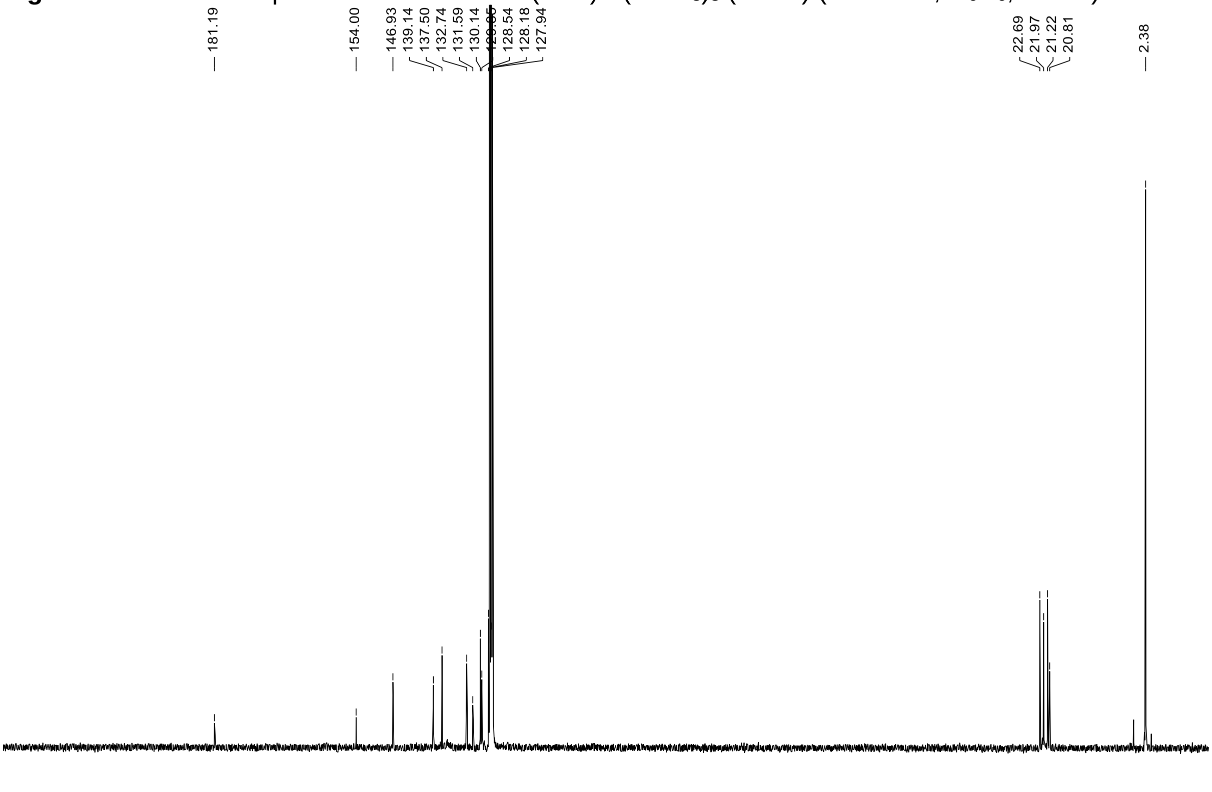

Figure S14: $^{13}C\{^{1}H\}$ NMR spectrum of ^{Mes}TerSnN(Mes)Si(SiMe$_3$)$_3$ (**Sn3b**) (101 MHz, C_6D_6, 298 K).

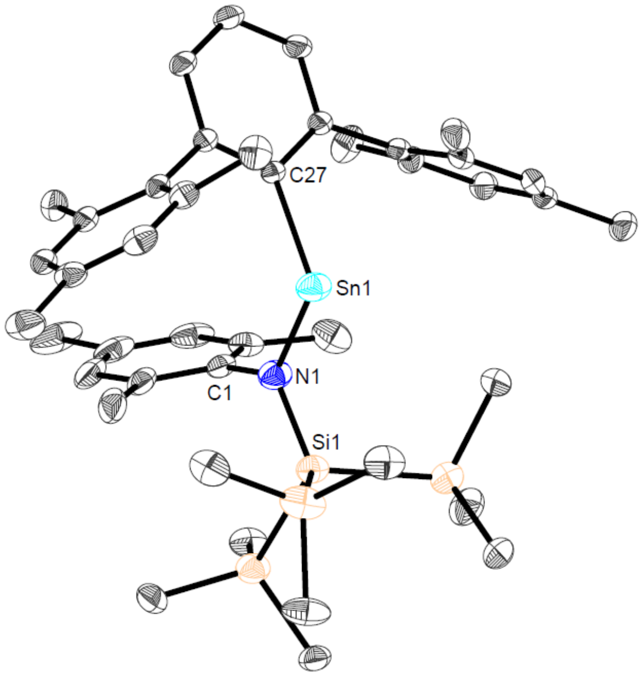

Figure S15. Molecular structure of ^{Mes}TerSnN(Mes)Si(SiMe$_3$)$_3$ (**Sn3b**) in the crystal. Thermal ellipsoids are drawn at the 50% probability level (hydrogen atoms have been omitted for clarity). Selected bond lengths (Å) and angles (deg): Sn1–N1 2.079(2), Sn1–C27 2.234(3), C27–Sn1–N1 109.49(9), C1–N1–Sn1 126.02(17), C1–N1–Si1 118.34(17), Si1–N1–Sn1 115.64(11).

Synthesis of ^{Mes}TerSnN(Dipp)Si(SiMe$_3$)$_3$ (Sn3c)

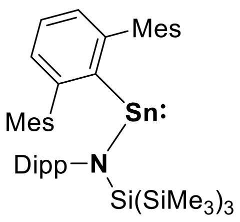

To a solution of ^{Mes}TerSnSi(SiMe$_3$)$_3$ (**Sn2**) (0.040 g, 0.059 mmol) in 0.3 mL of C_6D_6 was added a solution of N_3Dipp (**A3**) (0.012 g, 0.059 mmol) in 0.3 mL of C_6D_6 which results in immediate gas evolution and a colour change to red. All volatile components were removed under vacuum and the residue was dissolved in 1 mL of *n*-hexane, filtered and either stored at -30 °C to give ^{Mes}TerSnN(Dipp)Si(SiMe$_3$)$_3$ (**Sn3c**) as dark orange-red crystals or followed by removal of all volatile components to give **Sn3c** as a powder. The supernatant was removed via syringe and the crystals dried under vacuum. Crystals obtained this way were suitable for single crystal X-ray diffraction.

Yield: 0.032 g (0.037 mmol; 63%).
^{1}H NMR (400 MHz, C_6D_6, 298 K): δ = 0.14 (s, 27H, Si(CH$_3$)$_3$), 0.57 (d, $^3J_{H,H}$ = 6.7 Hz, 6H, CH(C*H*$_3$)$_2$), 1.26 (d, $^3J_{H,H}$ = 6.7 Hz, 6H, CH(C*H*$_3$)$_2$), 2.10 (s, 12H, CH$_3$), 2.21 (s, 6H, CH$_3$), 3.29 (hept, $^3J_{H,H}$ = 6.5 Hz, 2H, C*H*(CH$_3$)$_2$), 6.75 (s, 4H, CH$_{Aryl}$), 6.81-6.83 (m, 2H, CH$_{Aryl}$), 6.90-6.98 (m, 3H, CH$_{Aryl}$), 7.03-7.06 (m, 1H, CH$_{Aryl}$) ppm.
^{13}C{^{1}H} NMR (101 MHz, C_6D_6, 298 K): δ = 3.2 (Si(CH$_3$)$_3$), 21.1 (CH$_3$), 22.4 (CH$_3$), 27.1 (CH($\underline{C}$H$_3$)$_2$), 27.9 (CH($\underline{C}$H$_3$)$_2$), 28.5 ($\underline{C}$H(CH$_3$)$_2$), 123.7 (CH$_{Aryl}$), 124.2 (CH$_{Aryl}$), 128.0 (CH$_{Aryl}$)*,129.7 (CH$_{Aryl}$), 131.6 (CH$_{Aryl}$), 137.25 (C$_{q,Aryl}$), 137.32 (C$_{q,Aryl}$), 138.8 (C$_{q,Aryl}$), 142.3 (C$_{q,Aryl}$), 147.3 (C$_{q,Aryl}$), 152.7 (C$_{q,Aryl}$), 188.4 (C$_{q,Aryl}$Sn) ppm.
* = overlap with C_6D_6 signal (assigned by ^{1}H/^{13}C HSCQC)
^{29}Si{^{1}H} NMR (80 MHz, C_6D_6, 298 K): δ = -31.5, -15.7 ppm. (assigned by ^{1}H/^{29}Si HMBC)
^{119}Sn{^{1}H} NMR (149 MHz, C_6D_6, 298 K): δ = -99.0 ppm. (assigned by ^{1}H/^{119}Sn HMBC)

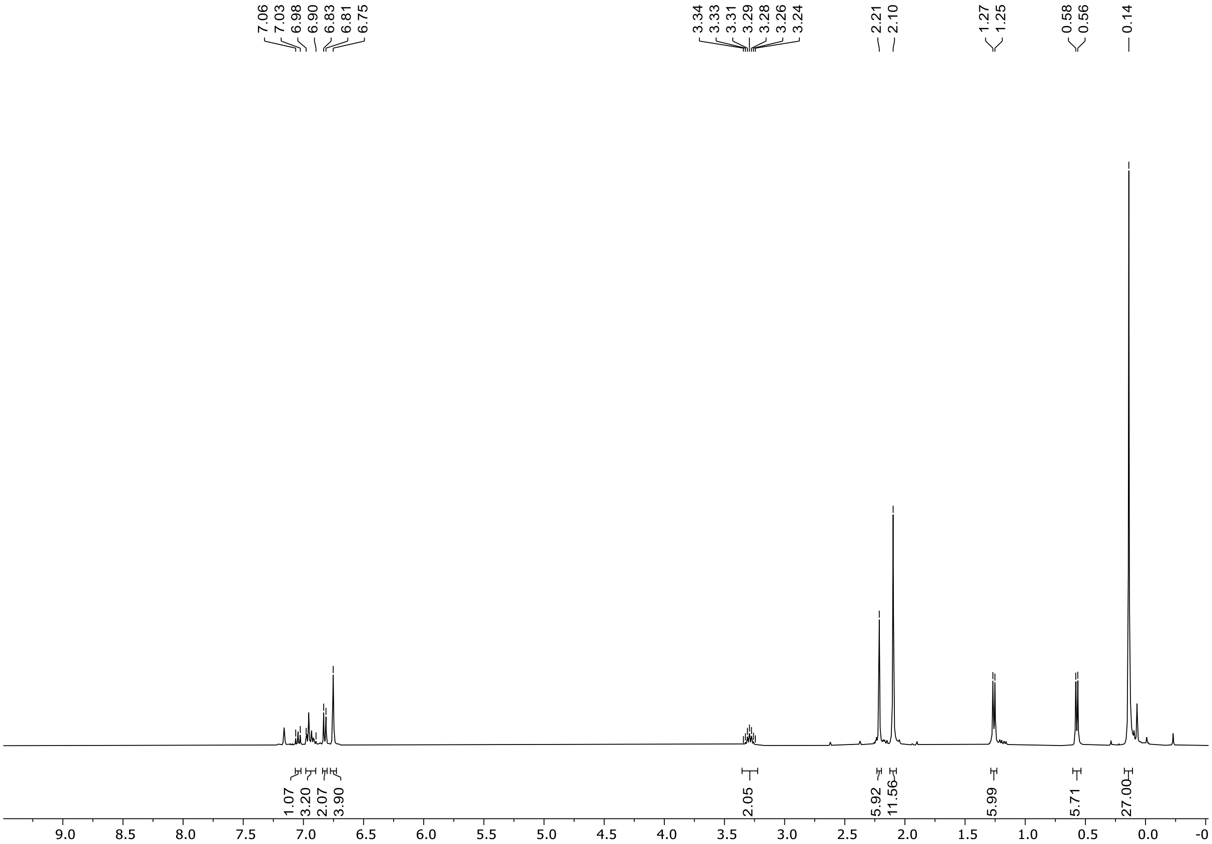

Figure S16. ^{1}H NMR spectrum of ^{Mes}TerSnN(Dipp)Si(SiMe$_3$)$_3$ (**Sn3c**) (400 MHz, C_6D_6, 298 K).

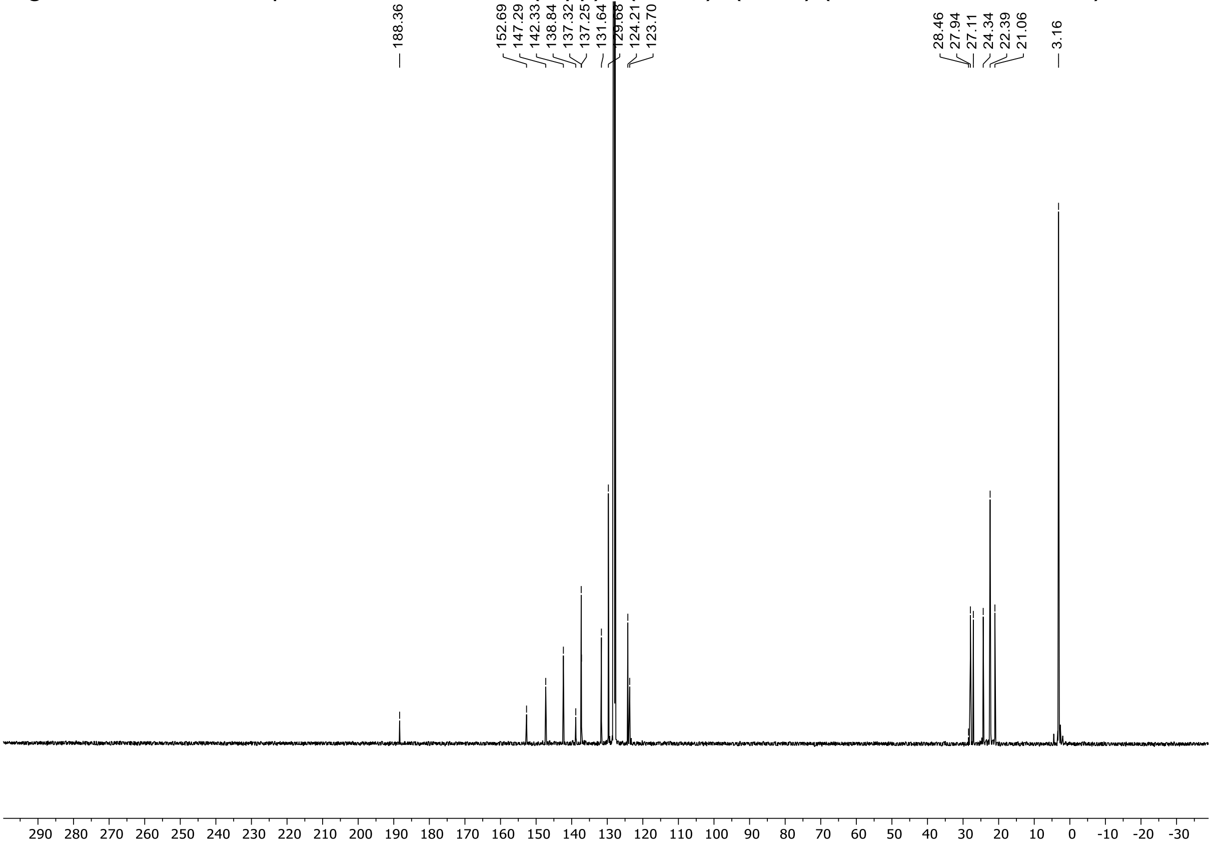

Figure S17: $^{13}C\{^{1}H\}$ NMR spectrum of ^{Mes}TerSnN(Dipp)Si(SiMe$_3$)$_3$ (**Sn3c**) (101 MHz, C_6D_6, 298 K).

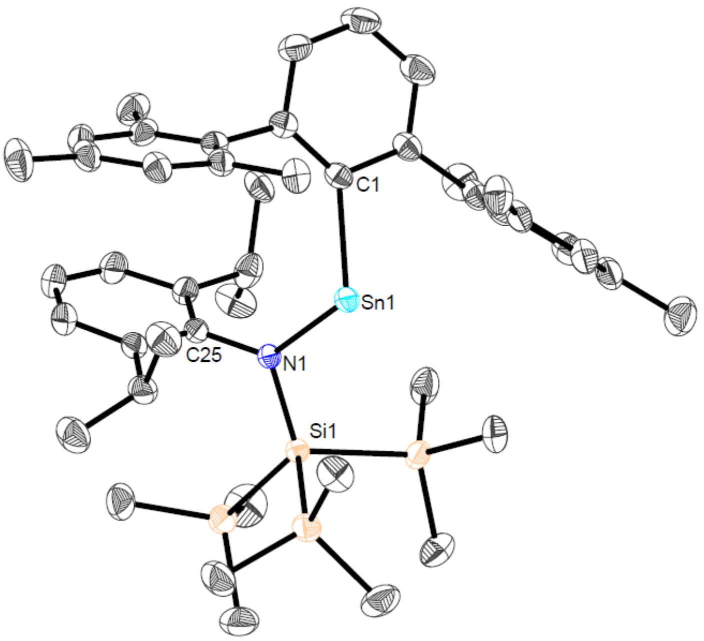

Figure S18. Molecular structure of ^{Mes}TerSnN(Dipp)Si(SiMe$_3$)$_3$ (**Sn3c**) in the crystal. Thermal ellipsoids are drawn at the 50% probability level (hydrogen atoms have been omitted for clarity). Selected bond lengths (Å) and angles (deg): Sn1–N1 2.096(2), Sn1–C1 2.244(2), C1–Sn1–N1 112.03(8), C25–N1–Sn1 126.89(14), C25–N1–Si1 120.89(16), Si1–N1–Sn1 111.62(9).

Attempted Reaction of ^{Mes}TerSnSi(SiMe$_3$)$_3$ (Sn2) with N$_3$^{Mes}Ter (A4)

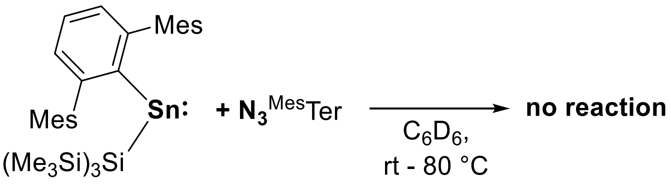

To a solution of ^{Mes}TerSnSi(SiMe$_3$)$_3$ (**Sn2**) (0.020 g, 0.029 mmol) in 0.3 mL of C_6D_6 was added a solution of N$_3$^{Mes}Ter (**A4**) (0.011 g, 0.029 mmol) in 0.3 mL of C_6D_6. Since subsequent NMR analysis revealed no reaction, the reaction mixture was heated to 80 °C for 24 h which also did not result in any reaction between both reactants. Shown below is the ^{1}H NMR spectrum of pure N$_3$^{Mes}Ter (**A4**) and the obtained ^{1}H NMR spectrum of the reaction mixture after 24 h at 80 °C.

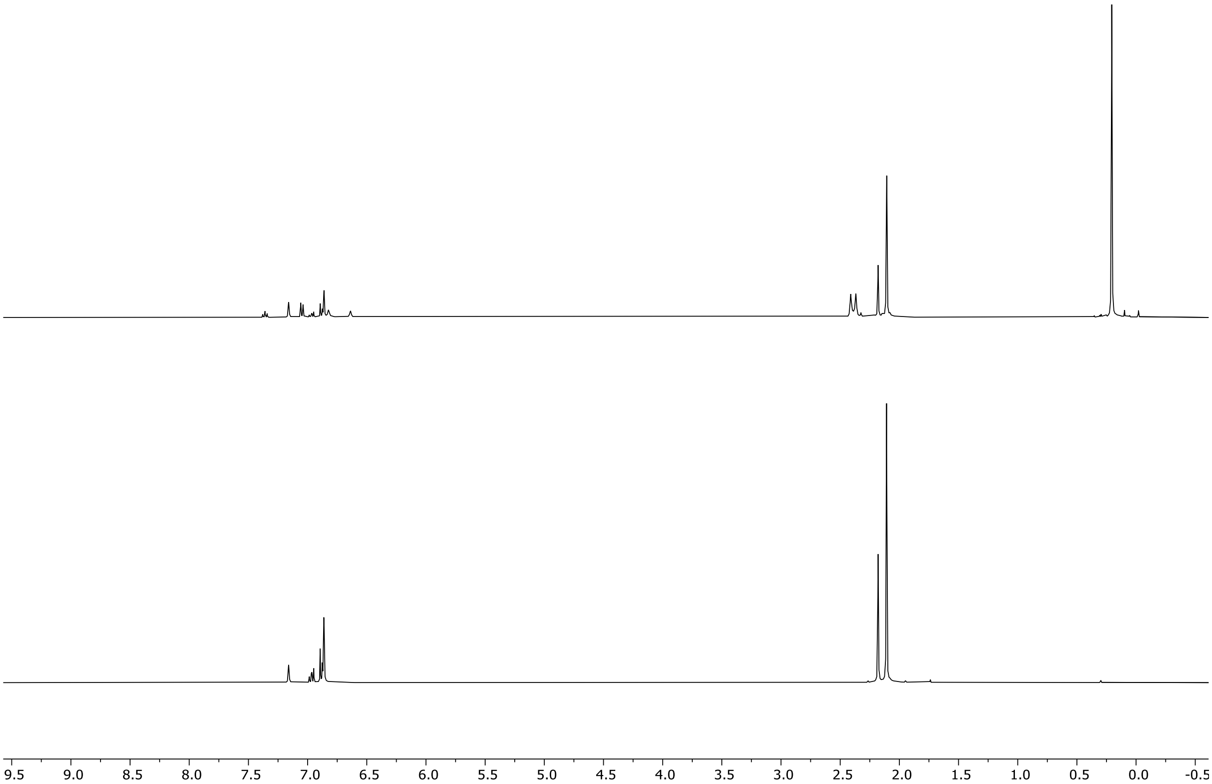

Figure S19. ^{1}H NMR spectrum of N$_3$^{Mes}Ter (**A4**) (bottom) and after the addition of ^{Mes}TerSnSi(SiMe$_3$)$_3$ (**Sn2**) after 24 h at 80 °C (400 MHz, C_6D_6, 298 K).

Synthesis of ^{Mes}TerSn(NHQuin)(QuinNSi(SiMe$_3$)$_2$NQuin) (Sn5)

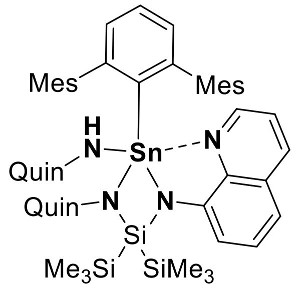

To a solution of ^{Mes}TerSnSi(SiMe$_3$)$_3$ (**Sn2**) (0.021 g, 0.031 mmol) in 0.3 mL of C_6D_6 was added a solution of N_3Quin (**A5**) (0.005 g, 0.021 mmol) in 0.3 mL of C_6D_6 which results in immediate gas evolution and is accompanied by a colour change to red. Due to the moderate solubility of **Sn2** in C_6D_6 and due to subsequent ^{1}H NMR analysis revealing that although **A5** was consumed completely significant amounts of ^{Mes}TerSnSi(SiMe$_3$)$_3$ (**Sn2**) remained unreacted, approximately 10 mg of **A5** in 0.1 mL of C_6D_6 were added to the reaction mixture leading again to gas evolution with a bright red solution remaining. All volatile components were removed under vacuum and the remaining solid was suspended in 0.6 mL of *n*-hexane, filtered and stored at -30 °C to give a small amount of bright red crystals suitable for single crystal X-ray diffraction. The product was shown to be ^{Mes}TerSn(NHQuin)(QuinNSi(SiMe$_3$)$_2$NQuin) (**Sn5**).

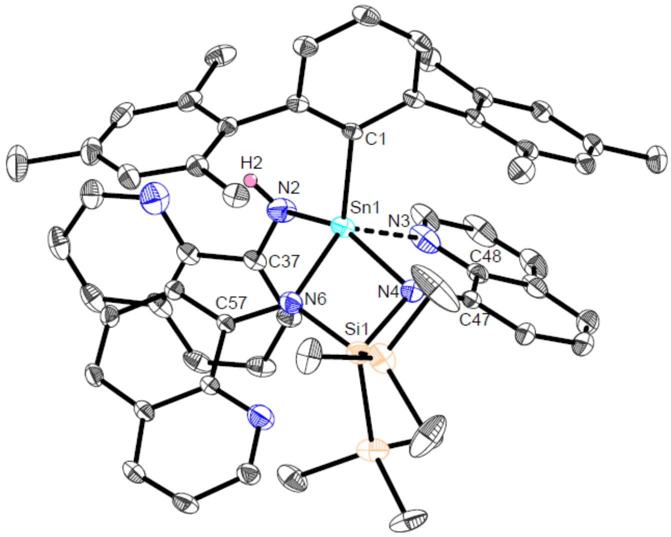

Figure S20. Molecular structure of ^{Mes}TerSn(NHQuin)(QuinNSi(SiMe$_3$)$_2$NQuin) (**Sn5**) in the crystal. Thermal ellipsoids are drawn at the 50% probability level (hydrogen atoms have been omitted for clarity). Selected bond lengths (Å) and angles (deg): Sn1–N2 2.0719(14), Sn1–N3 2.3256(15), Sn1–N4 2.0808(13), Sn1–N6 2.1162(12), Sn1–C1 2.1765(15), N4–Si1 1.7965(14), N6–Si1 1.7685(14), N2–Sn1–N6 101.39(5), N6–Sn1–N4 72.06(5), N4–Sn1–N3 74.31(6), C1–Sn1–N2 105.44(6), C1–Sn1–N6 117.90(5), C1–Sn1–N4 108.10(6), C1–Sn1–N3 110.20(5).

Synthesis of ^{Mes}TerSn(N(SiMe$_3$)$_2$)$_2$N$_3$ (Sn6)

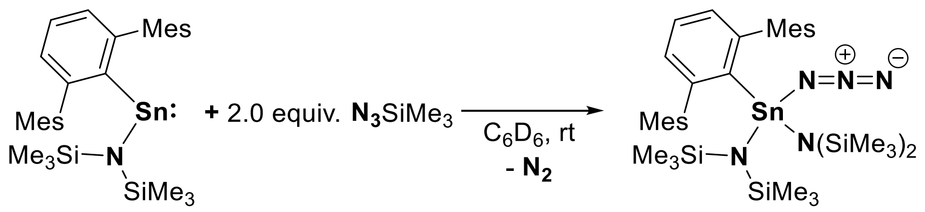

To a solution of ^{Mes}TerSnN(SiMe$_3$)$_2$ (**Sn1**) (0.050 g, 0.084 mmol) in 0.3 mL of C_6D_6 was added a solution of N_3SiMe_3 (**A1**) (0.010 g, 0.084 mmol) in 0.3 mL of C_6D_6. Subsequent NMR analysis of the reaction mixture revealed fast consumption of **A1** with approximately 0.5 equivalents of **Sn1** remaining unreacted. Therefore, additional amounts of **A1** was added leading to full consumption of **Sn1** and formation of ^{Mes}TerSn(N(SiMe$_3$)$_2$)$_2$N$_3$ (**Sn6**). All volatile components were removed under vacuum and the slightly yellow solid was dissolved in 0.7 mL of *n*-hexane, filtered and stored at -30 °C to give **Sn6** as colourless crystals. Crystals obtained this way were suitable for single crystal X-ray diffraction.

Yield: 0.017 g (0.021 mmol; 25%).
^{1}H NMR (400 MHz, C_6D_6, 298 K): δ = 0.27 (s, 36H, Si(CH$_3$)$_3$), 2.20 (s, 12H, CH$_3$), 2.24 (s, 6H, CH$_3$), 6.76-6.77 (m, 2H, CH$_{Aryl}$), 6.91 (s, 4H, CH$_{Aryl}$), 6.99-7.02 (m, 1H, CH$_{Aryl}$) ppm.
^{13}C{^{1}H} NMR (101 MHz, C_6D_6, 298 K): δ = 7.0 (Si(CH$_3$)$_3$), 21.1 (CH$_3$), 23.5 (CH$_3$), 129.6 (CH$_{Aryl}$), 131.0 (CH$_{Aryl}$), 132.6 (CH$_{Aryl}$), 136.6 (C$_{q,Aryl}$), 137.8 (C$_{q,Aryl}$), 140.2 (C$_{q,Aryl}$), 143.2 (C$_{q,Aryl}$), 150.2 (C$_{q,Aryl}$) ppm.
^{29}Si{^{1}H} NMR (80 MHz, C_6D_6, 298 K): δ = 6.1 ppm. (assigned by ^{1}H/^{29}Si HMBC)
^{119}Sn{^{1}H} NMR (149 MHz, C_6D_6, 298 K): δ = -213.1 ppm.
EA: Anal. calcd. for $C_{36}H_{61}N_5Si_4Sn$: C, 54.39; H, 7.73; N, 8.81; Found: C, 55.11; H, 7.81; N, 8.21.

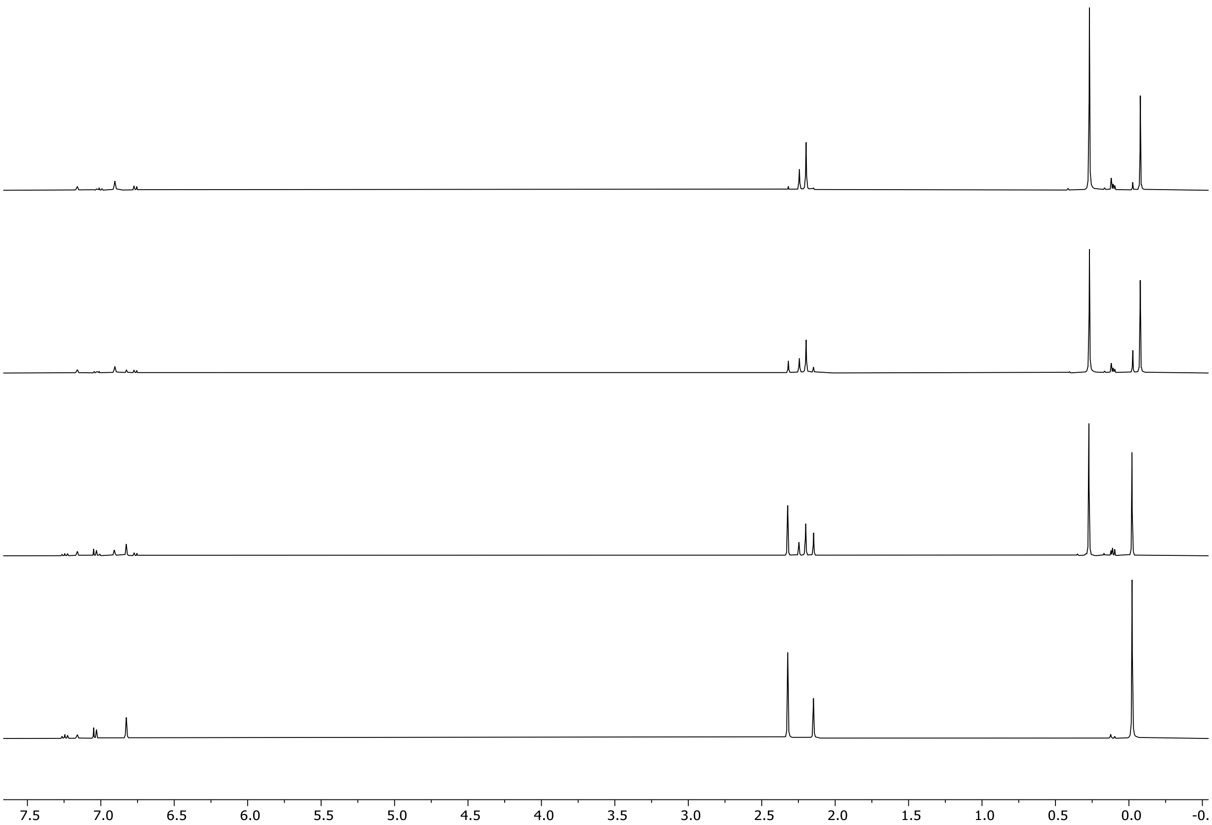

Figure S21. Monitoring of the reaction of ^{Mes}TerSnN(SiMe$_3$)$_2$ (**Sn1**) with N_3SiMe_3 (**A1**) via ^{1}H NMR spectroscopy (400 MHz, C_6D_6, 298 K): Bottom: Pure **Sn1**, second from bottom: after addition of 1.0 equivalents of N_3SiMe_3 (**A1**), second from top: after addition of additional N_3SiMe_3 (**A1**), top: after additional 16 h at room temperature; -0.08 ppm: N_3SiMe_3.

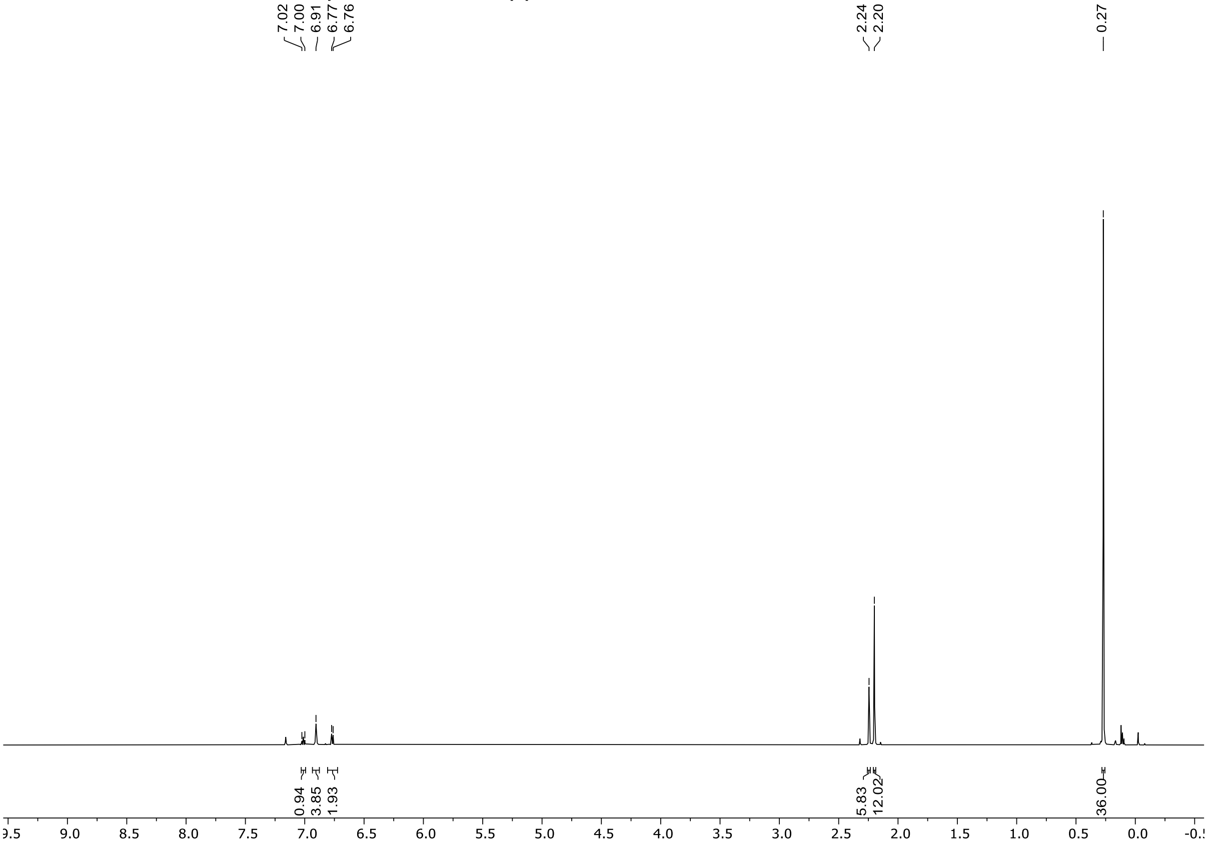

Figure S22. ^{1}H NMR spectrum of ^{Mes}TerSn(N(SiMe$_3$)$_2$)$_2$N$_3$ (**Sn6**) (400 MHz, C_6D_6, 298 K).

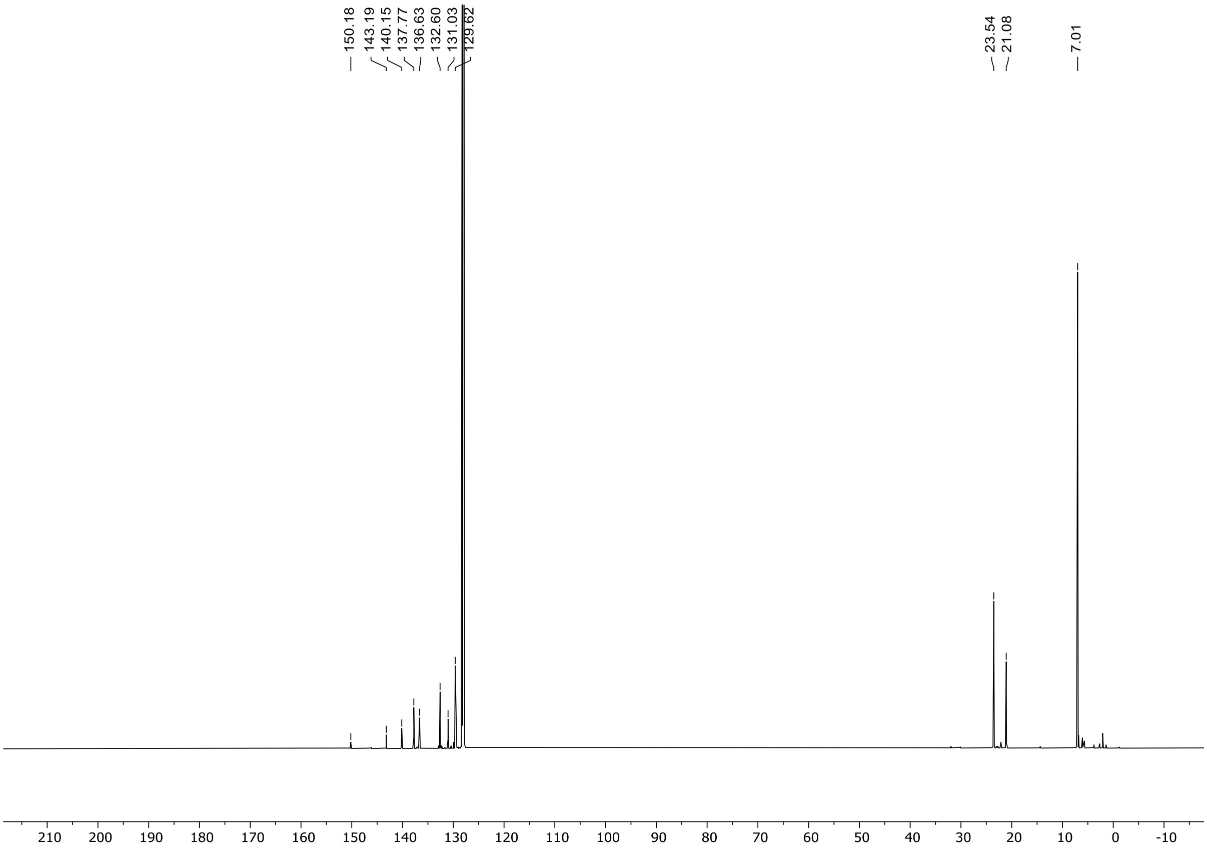

Figure S23. $^{13}C\{^1H\}$ NMR spectrum of $^{Mes}TerSn(N(SiMe_3)_2)_2N_3$ (**Sn6**) (101 MHz, C_6D_6, 298 K).

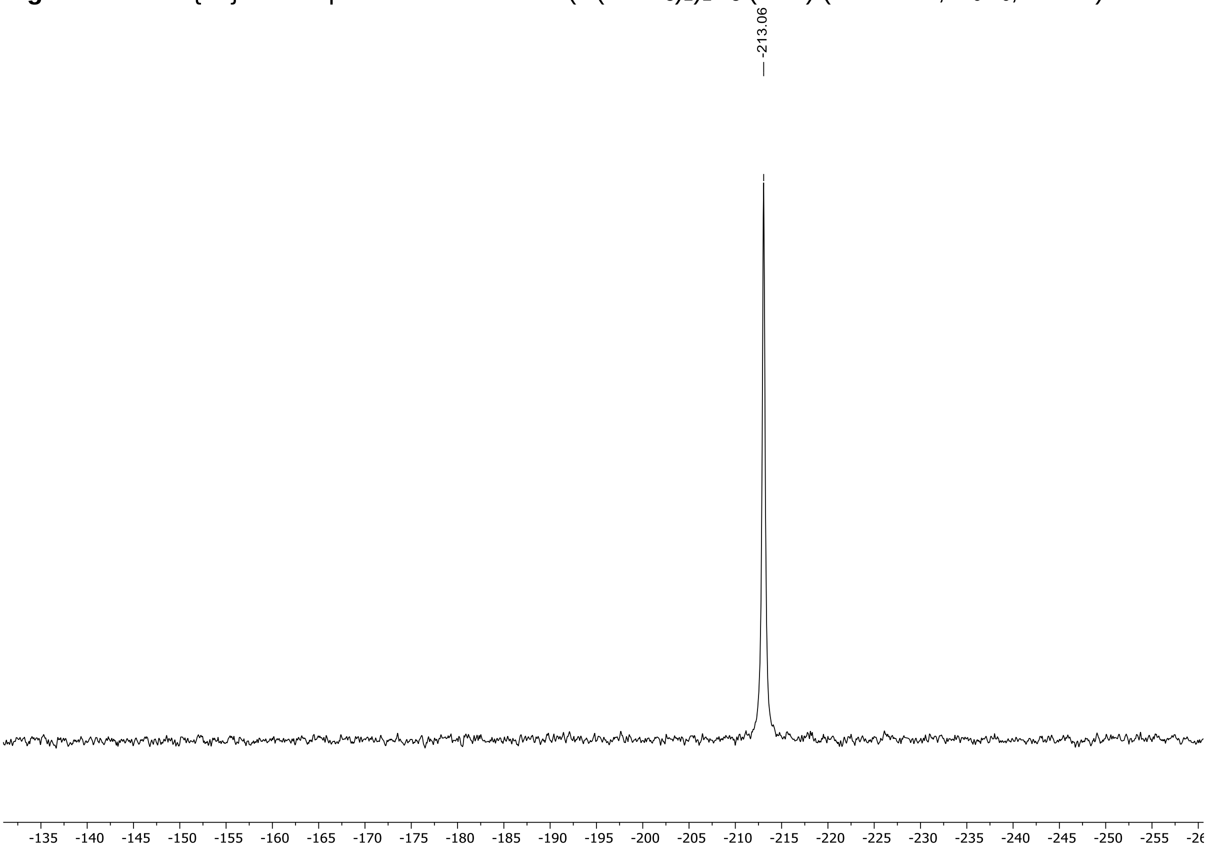

Figure S24. $^{119}Sn\{^1H\}$ NMR spectrum of $^{Mes}TerSn(N(SiMe_3)_2)_2N_3$ (**Sn6**) (149 MHz, C_6D_6, 298 K).

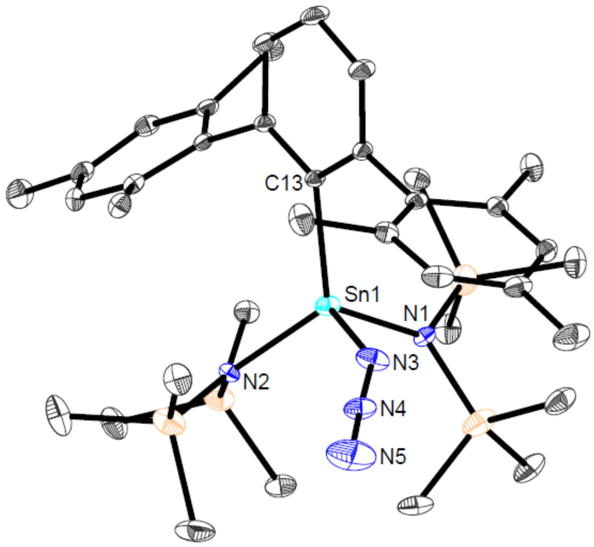

Figure S25. Molecular structure of $^{Mes}TerSn(N(SiMe_3)_2)_2N_3$ (**Sn6**) in the crystal. Thermal ellipsoids are drawn at the 50% probability level (hydrogen atoms have been omitted for clarity). Selected bond lengths (Å) and angles (deg): Sn1–N1 2.052(3), Sn1–N2 2.063(3), Sn1–N3 2.059(3), Sn1–C13 2.217(3), N3–N4 1.167(5), N4–N5 1.143(6), N2–Sn1–C13 120.94(11), N2–Sn1–N3 105.29(13).

Synthesis of ^{Mes}TerSn(N(SiMe$_3$)$_2$)=NMes (Sn7a) and of the C–H-Activation Product Sn4b

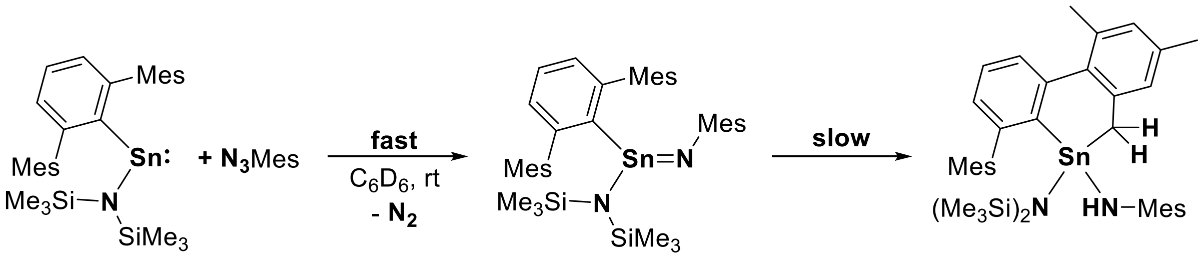

To a solution of ^{Mes}TerSnN(SiMe$_3$)$_2$ (**Sn1**) (0.040 g, 0.068 mmol) in 0.3 mL of C_6D_6 was added a solution of N$_3$Mes (**A2**) (0.011 g, 0.068 mmol) in 0.3 mL of C_6D_6 leading to immediate gas evolution and a colour change to orange-red. Subsequent NMR analysis revealed clean formation of ^{Mes}TerSn(N(SiMe$_3$)$_2$)=NMes (**Sn7a**). Removal of all volatile components yielded **Sn7a** as an orange-red solid.
Dark orange-red crystals of **Sn7a** suitable for single crystal X-ray diffraction were obtained from a saturated *n*-hexane solution of **Sn7a** at -30 °C.
In solution **Sn7a** undergoes intramolecular C–H-activation to give **Sn4b**. Therefore, only the ^{1}H NMR data of ^{Mes}TerSn(N(SiMe$_3$)$_2$)=NMes (**Sn7a**) is given below. To obtain analytically pure **Sn4b**, the above-mentioned reaction mixture was heated to 70 °C for 16 h resulting in a colour change to pale yellow. All volatile components were removed under vacuum. The residue was suspended in 0.7 mL of *n*-hexane, filtered and stored at 4 °C to give **Sn4b** as colourless crystals. Crystals obtained this way were suitable for single crystal X-ray diffraction.

Note: ^{Mes}TerSn(N(SiMe$_3$)$_2$)=NMes (**Sn7a**) even in the solid state slowly undergoes intramolecular C–H-activation to give **Sn4b**. Due to this behaviour, **Sn7a** was synthesized *in situ* and directly reacted further for the reactivity studies. Although **Sn7a** can be obtained repeatedly as a single crystalline material following the above procedure, we recommend to follow the *in situ* protocol.

Characteristic NMR data of ^{Mes}TerSn(N(SiMe$_3$)$_2$)=NMes (Sn7a):
^{1}H NMR (400 MHz, C_6D_6, 298 K): δ = -0.16 (s, 18H, Si(CH$_3$)$_3$), 2.14 (s, 6H, CH$_3$), 2.27-2.28 (s, 9H, CH$_3$), 2.38 (s, 12H, CH$_3$), 6.84-6.85 (m, 4H, CH$_{Aryl}$), 6.95-6.97 (m, 2H, CH$_{Aryl}$), 7.00-7.01 (m, 2H, CH$_{Aryl}$), 7.15-7.19 (m, 1H, CH$_{Aryl}$)* ppm.
* = overlap with C_6D_5H signal
^{13}C{^{1}H} NMR (101 MHz, C_6D_6, 298 K): δ = 4.7 (Si(CH$_3$)$_3$), 20.9 (CH$_3$), 21.0 (CH$_3$), 21.1 (CH$_3$), 22.2 (CH$_3$), 127.3 (C$_{q,Aryl}$), 128.3 (C$_{q,Aryl}$), 128.8 (CH$_{Aryl}$), 129.4 (CH$_{Aryl}$), 129.6 (CH$_{Aryl}$), 131.0 (CH$_{Aryl}$), 138.1 (C$_{q,Aryl}$), 138.9 (C$_{q,Aryl}$), 139.9 (C$_{q,Aryl}$), 148.2 (C$_{q,Aryl}$), 152.9 (C$_{q,Aryl}$), 155.0 (C$_{q,Aryl}$) ppm.
^{119}Sn{^{1}H} NMR (149 MHz, C_6D_6, 298 K): δ = 54.3 ppm. (assigned by ^{1}H/^{119}Sn HMBC)

Analytical data of C–H-activation product Sn4b:
Yield: 0.026 g (0.036 mmol; 53%).
^{1}H NMR (400 MHz, C_6D_6, 298 K): δ = 0.01 (s, 18H, Si(CH$_3$)$_3$), 2.10-2.11 (m, 9H, CH$_3$), 2.14 (s, 3H, CH$_3$), 2.19 (s, 9H, CH$_3$), 2.21 (s, 1H, NH), 2.26 (s, 3H, CH$_3$), 2.50 (d, $^{2}J_{H,H}$ = 11.2 Hz, Sn satellites: $^{2}J_{119Sn,H}$ = 75.5 Hz, $^{2}J_{117Sn,H}$ = 53.3 Hz, 1H, CH$_2$), 2.91 (d, $^{2}J_{H,H}$ = 11.0 Hz, Sn satellites: $^{2}J_{119Sn,H}$ = 91.2 Hz, $^{2}J_{117Sn,H}$ = 68.4 Hz, 1H, CH$_2$), 6.58-6.59 (m, 1H, CH$_{Aryl}$), 6.78-6.81 (m, 3H, CH$_{Aryl}$), 6.86-6.89 (m, 3H, CH$_{Aryl}$), 7.20-7.26 (m, 2H, CH$_{Aryl}$) ppm.
^{13}C{^{1}H} NMR (101 MHz, C_6D_6, 298 K): δ = 5.3 (Si(CH$_3$)$_3$), 19.4 (CH$_3$), 20.8 (CH$_3$), 21.0 (CH$_3$), 21.1 (CH$_3$), 22.2 (CH$_3$), 22.3 (CH$_3$), 22.6 (CH$_3$), 33.6 (Sn satellites: $^{1}J_{119Sn,C}$ = 475.0 Hz, $^{1}J_{117Sn,C}$ = 453.2 Hz, CH$_2$), 128.3 (CH$_{Aryl}$),* 128.5 (CH$_{Aryl}$), 128.6 (CH$_{Aryl}$), 128.8 (CH$_{Aryl}$), 128.9 (CH$_{Aryl}$), 129.4 (CH$_{Aryl}$), 129.7 (C$_{q,Aryl}$), 130.5 (CH$_{Aryl}$), 131.4 (CH$_{Aryl}$), 134.9 (C$_{q,Aryl}$), 136.3 (C$_{q,Aryl}$), 136.6 (C$_{q,Aryl}$), 137.0 (C$_{q,Aryl}$), 137.2 (C$_{q,Aryl}$), 137.3 (C$_{q,Aryl}$), 138.2 (C$_{q,Aryl}$), 140.9 (C$_{q,Aryl}$), 144.3 (C$_{q,Aryl}$), 147.2 (C$_{q,Aryl}$), 147.5 (C$_{q,Aryl}$), 147.7 (C$_{q,Aryl}$) ppm.

* = overlap with C_6D_6 signal (assigned by $^1H/^{13}C$ HSCQC)

$^{29}Si\{^1H\}$ NMR (80 MHz, C_6D_6, 298 K): δ = 4.1 ppm. (assigned by $^1H/^{29}Si$ HMBC)

$^{119}Sn\{^1H\}$ NMR (149 MHz, C_6D_6, 298 K): δ = -135.5 ppm.

EA: Anal. calcd. for $C_{39}H_{51}N_2Si_2Sn$: C, 64.54; H, 7.50; N, 3.86; Found: C, 65.01; H, 7.72; N, 3.73.

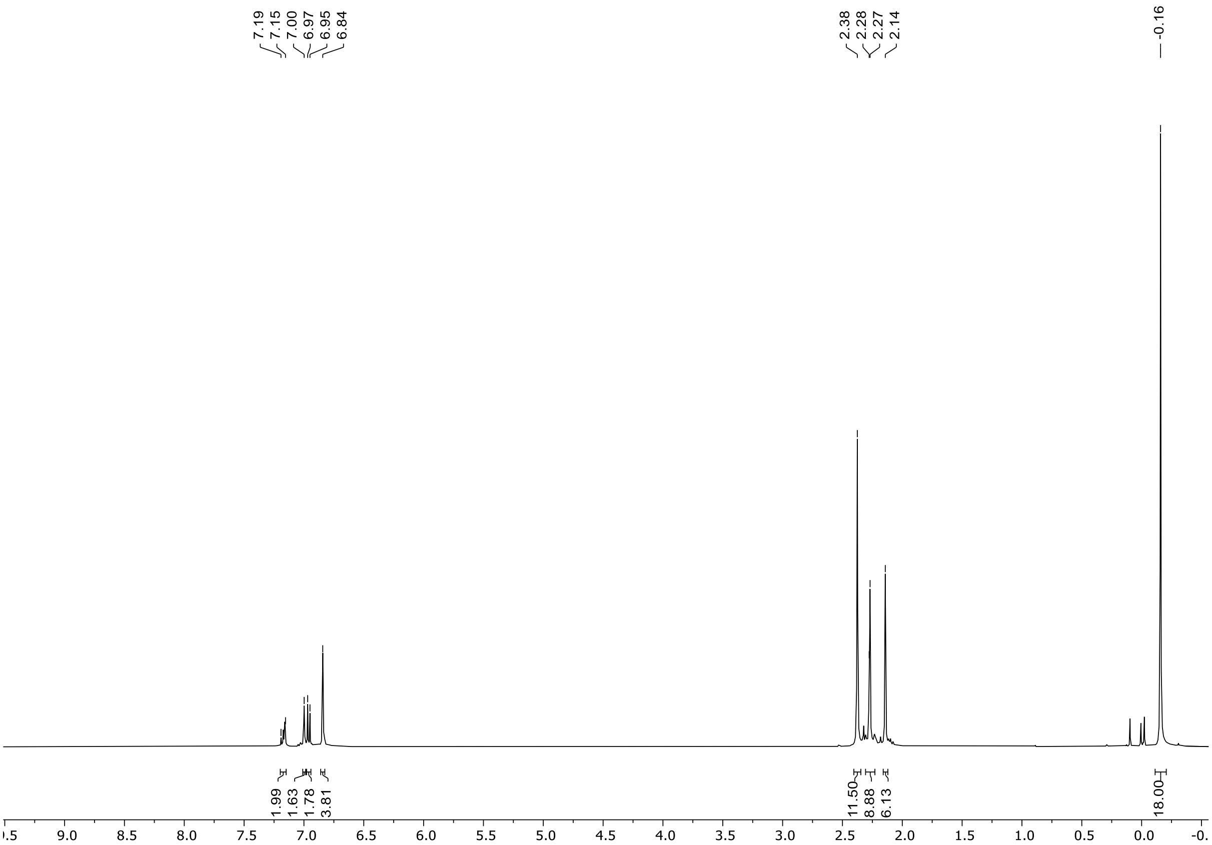

Figure S26. ^1H NMR spectrum measured after 5 minutes of $^{Mes}TerSn(N(SiMe_3)_2)$=NMes (**Sn7a**) (400 MHz, C_6D_6, 298 K).

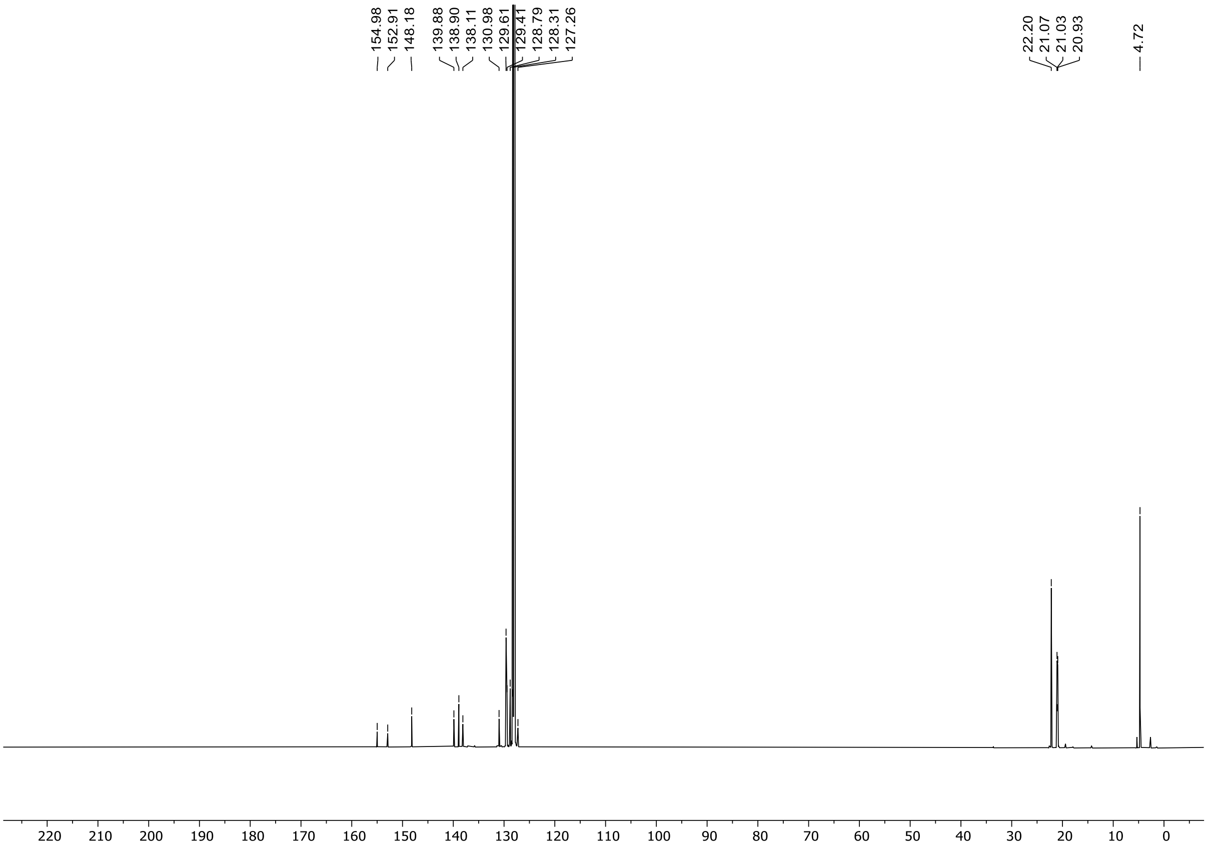

Figure S27. $^{13}C\{^1H\}$ NMR spectrum of $^{Mes}TerSn(N(SiMe_3)_3)=NMes$ (**Sn7a**) (126 MHz, C_6D_6, 298 K).

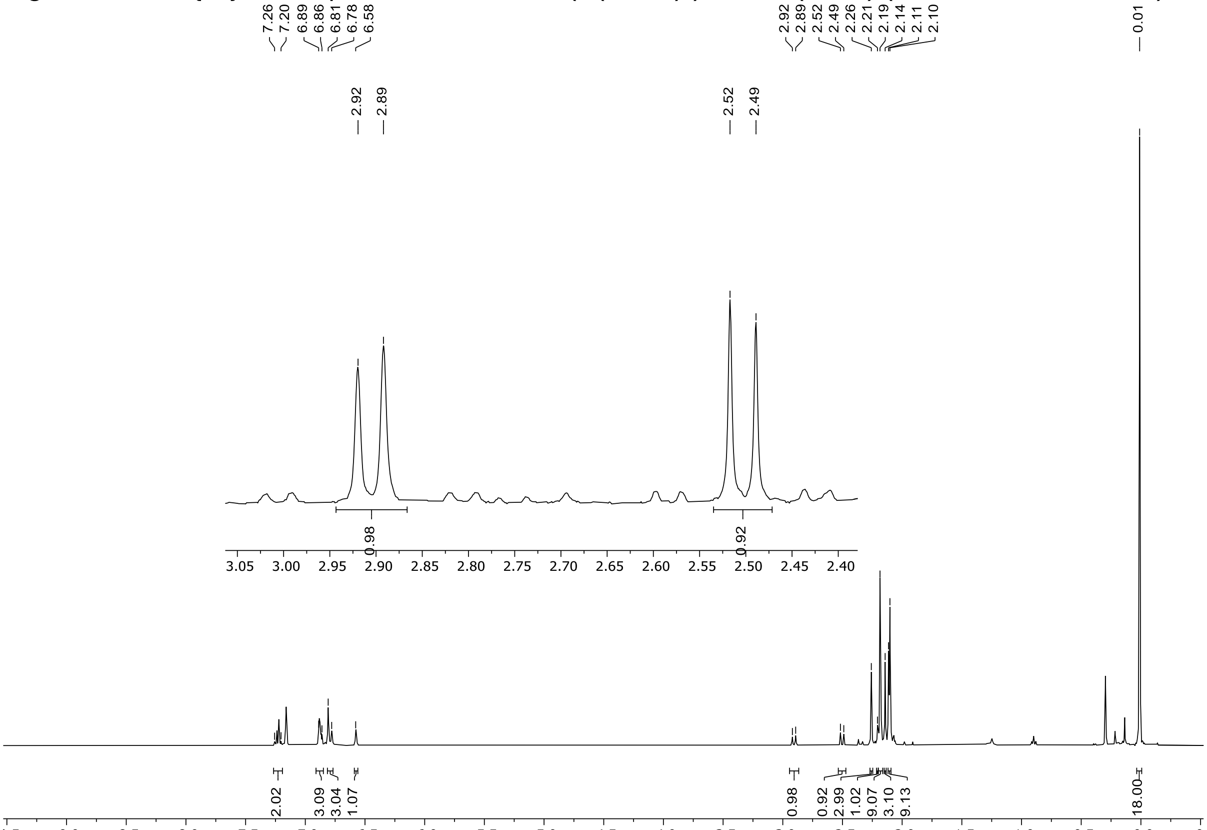

Figure S28. ^1H NMR spectrum of the C–H-activation product **Sn4b** (400 MHz, C_6D_6, 298 K); 0.29 ppm: silicon grease.

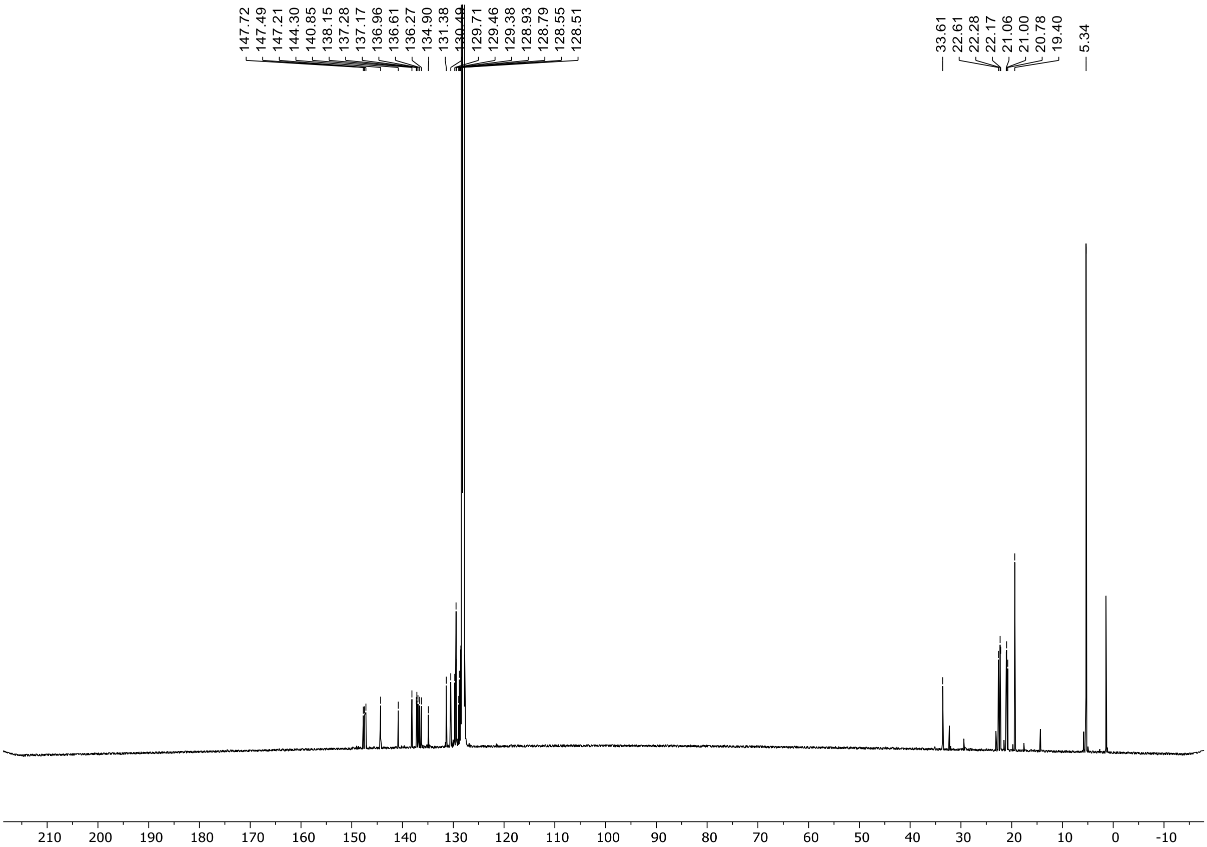

Figure S29. $^{13}C\{^1H\}$ NMR spectrum of the C–H-activation product **Sn4b** (126 MHz, C_6D_6, 298 K); 1.4 ppm: silicon grease.

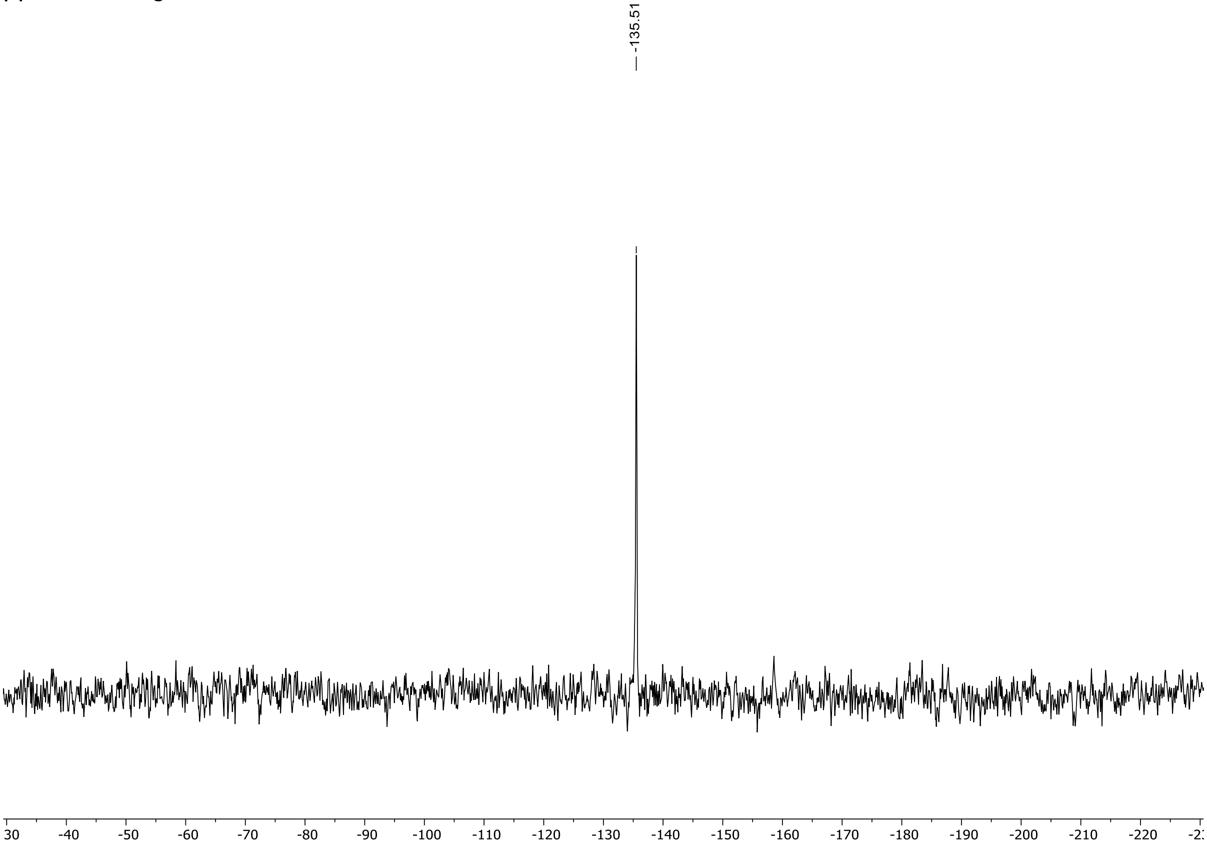

Figure S30. $^{119}Sn\{^1H\}$ NMR spectrum of the C–H-activation product **Sn4b** (149 MHz, C_6D_6, 298 K).

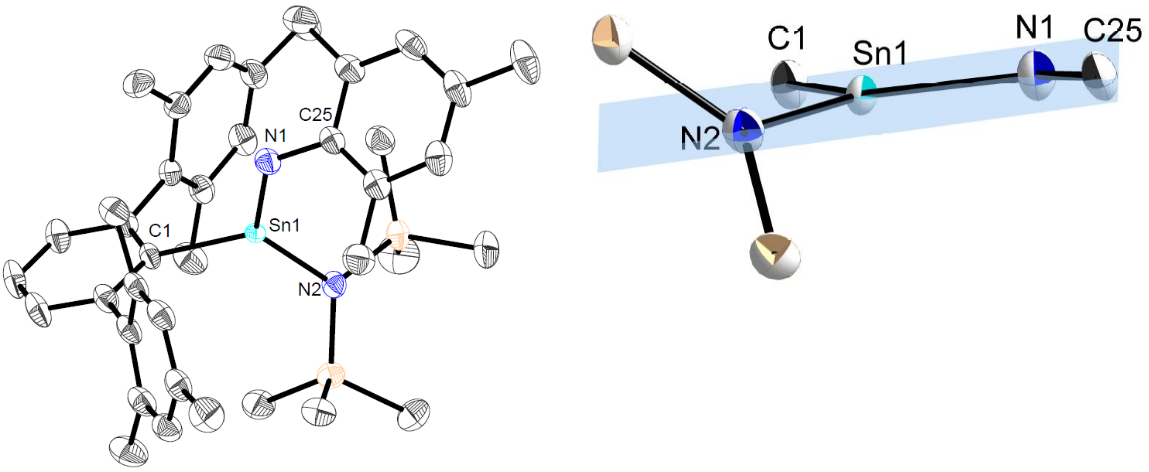

Figure S31. Molecular structure of ^{Mes}TerSnN(N(SiMe$_3$)$_2$)=NMes (**Sn7a**) in the crystal. Thermal ellipsoids are drawn at the 50% probability level (hydrogen atoms have been omitted for clarity). Selected bond lengths (Å) and angles (deg): Sn1–N1 1.9354(16), Sn1–N2 2.0455(16), Sn1–C1 2.1481(18), N1–C25 1.401(2), C1–Sn1–N1 116.46(7), N1–Sn1–N2 125.17(7), C1–Sn1–N2 118.34(7).

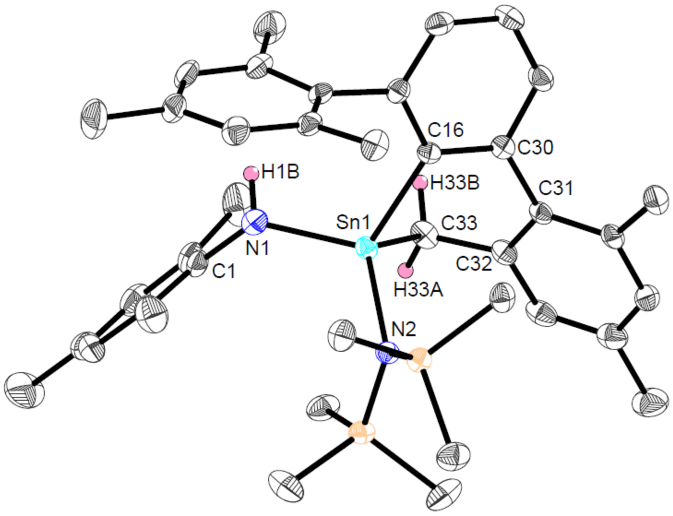

Figure S32. Molecular structure of C–H activation product **Sn4b** in the crystal. Thermal ellipsoids are drawn at the 50% probability level (hydrogen atoms (except H3, H1A and H1B) have been omitted for clarity). Selected bond lengths (Å) and angles (deg): Sn1–N1 2.037(2), Sn1–N2 2.0566(19), Sn1–C33 2.147(2), Sn1–C16 2.159(2), C32–C33 1.495(4), C31–C32 1.411(3), C30–C31 1.500(3), C16–C30 1.421(3), N1–Sn1–N2 112.30(8), N2–Sn1–C33 110.64(9).

Synthesis of ^{Mes}TerSn(N(SiMe$_3$)$_2$)=NDipp (Sn7b) and of the C–H-Activation Product Sn4c

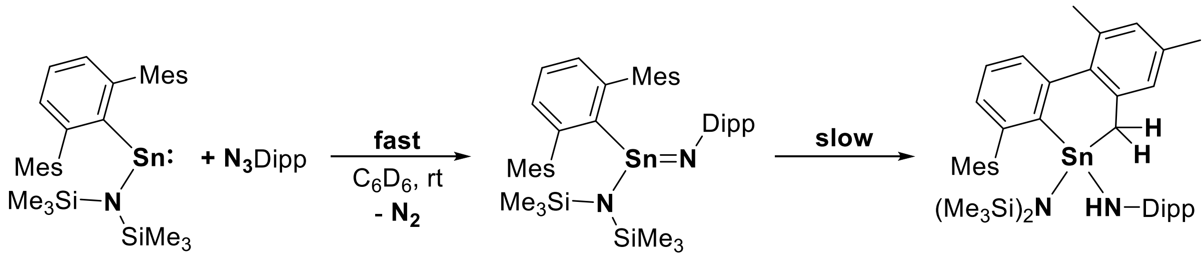

To a solution of ^{Mes}TerSnN(SiMe$_3$)$_2$ (**Sn1**) (0.050 g, 0.084 mmol) in 0.3 mL of C_6D_6 was added a solution of N$_3$Dipp (**A3**) (0.017 g, 0.084 mmol) in 0.3 mL of C_6D_6 leading to immediate gas evolution and a colour change to orange-red. Subsequent NMR analysis revealed clean formation of ^{Mes}TerSn(N(SiMe$_3$)$_2$)=NDipp (**Sn7b**). Removal of all volatile components yielded **Sn7b** as an orange-red solid.
In solution **Sn7b** undergoes intramolecular C–H-activation to give **Sn4c**. Therefore, only the ^{1}H NMR data of ^{Mes}TerSn(N(SiMe$_3$)$_2$)=NDipp (**Sn7b**) is given below. To obtain analytically pure **Sn4c**, the above-mentioned reaction mixture was heated to 70 °C for 16 h resulting in a colour change to pale orange. All volatile components were removed under vacuum. The residue was suspended in 0.7 mL of *n*-hexane, filtered and stored at 4 °C to give **Sn4b** as colourless crystals. Crystals obtained this way were suitable for single crystal X-ray diffraction.

Note: ^{Mes}TerSn(N(SiMe$_3$)$_2$)=NDipp (**Sn7b**) even in the solid state slowly undergoes intramolecular C–H-activation to give **Sn4c**. Due to this behaviour, **Sn4c** was synthesized *in situ* and directly reacted further for the reactivity studies. Although **Sn7b** can be obtained repeatedly as a single crystalline material following the above procedure, we recommend to follow the *in situ* protocol.

Characteristic NMR data of ^{Mes}TerSn(N(SiMe$_3$)$_2$)=NDipp (Sn7b):
^{1}H NMR (400 MHz, C_6D_6, 298 K): δ = -0.23 (s, 18H, Si(CH$_3$)$_3$), 1.28 (d, $^3J_{H,H}$ = 6.9 Hz, 12H, CH(C*H*$_3$)$_2$), 2.15 (s, 6H, CH$_3$), 2.38 (s, 12H, CH$_3$), 3.21 (hept, $^3J_{H,H}$ = 6.9 Hz, 2H, C*H*(CH$_3$)$_2$), 6.86-6.87 (m, 4H, CH$_{Aryl}$), 6.94-6.97 (m, 3H, CH$_{Aryl}$), 7.15-7.19 (m, 1H, CH$_{Aryl}$)*, 7.21-7.23 (m, 2H, CH$_{Aryl}$) ppm.
* = overlap with C_6D_5H signal
^{13}C{^{1}H} NMR (101 MHz, C_6D_6, 298 K): δ = 4.5 (Si(CH$_3$)$_3$), 21.1 (CH$_3$), 22.2 (CH$_3$), 23.9 (br, CH(CH$_3$)$_2$), 28.7 (CH(CH$_3$)$_2$), 120.1 (CH$_{Aryl}$), 122.1 (CH$_{Aryl}$), 129.6 (CH$_{Aryl}$), 129.8 (CH$_{Aryl}$), 131.1 (CH$_{Aryl}$), 138.0 (br, C$_{q,Aryl}$), 138.8 (C$_{q,Aryl}$), 138.9 (C$_{q,Aryl}$), 139.8 (C$_{q,Aryl}$), 148.2 (C$_{q,Aryl}$), 152.3 (C$_{q,Aryl}$), 154.5 (C$_{q,Aryl}$) ppm.
^{119}Sn{^{1}H} NMR (149 MHz, C_6D_6, 298 K): δ = 48.5 ppm. (assigned by ^{1}H/^{119}Sn HMBC)

Analytical data of C–H-activation product Sn4c:
Yield: 0.019 g (0.025 mmol; 30%).
^{1}H NMR (400 MHz, C_6D_6, 298 K): δ = -0.04 (s, 18H, Si(CH$_3$)$_3$), 1.07-1.09 (m, 6H, CH(C*H*$_3$)$_2$), 1.27 (d, $^3J_{H,H}$ = 6.6 Hz, 6H, CH(C*H*$_3$)$_2$), 2.11 (s, 3H, CH$_3$), 2.17 (s, 3H, CH$_3$), 2.18 (s, 3H, CH$_3$), 2.29 (s, 3H, CH$_3$), 2.30 (s, 3H, CH$_3$), 2.51 (s, 1H, Sn satellites: $^2J_{119Sn,H}$ = 55.7 Hz, $^2J_{117Sn,H}$ = 45.6 Hz, NH), 2.63 (d, $^2J_{H,H}$ = 11.4 Hz, Sn satellites: $^2J_{119Sn,H}$ = 63.2 Hz, $^2J_{117Sn,H}$ = 46.4 Hz, 1H, CH$_2$), 2.88 (d, $^2J_{H,H}$ = 11.2 Hz, Sn satellites: $^2J_{119Sn,H}$ = 90.6 Hz, $^2J_{117Sn,H}$ = 68.2 Hz, 1H, CH$_2$), 3.19-3.26 (m, 2H, C*H*(CH$_3$)$_2$), 6.77-6.88 (m, 5H, CH$_{Aryl}$), 6.96-7.02 (m, 1H, CH$_{Aryl}$), 7.09-7.11 (m, 2H, CH$_{Aryl}$), 7.21-7.26 (m, 2H, CH$_{Aryl}$) ppm.
^{13}C{^{1}H} NMR (101 MHz, C_6D_6, 298 K): δ = 5.5 (Si(CH$_3$)$_3$), 21.1 (CH$_3$), 21.2 (CH$_3$), 22.3 (CH$_3$), 22.5 (CH$_3$), 22.7 (CH$_3$), 23.9 (br, CH(CH$_3$)$_2$), 24.7 (br, CH(CH$_3$)$_2$), 27.9 (CH(CH$_3$)$_2$), 31.9 (Sn satellites: $^1J_{119Sn,C}$ = 475.6 Hz, $^1J_{117Sn,C}$ = 453.9 Hz, CH$_2$), 122.4 (CH$_{Aryl}$), 123.1 (CH$_{Aryl}$), 128.6 (CH$_{Aryl}$), 128.8 (CH$_{Aryl}$), 129.0 (CH$_{Aryl}$), 129.7 (CH$_{Aryl}$), 130.6 (CH$_{Aryl}$), 131.7 (CH$_{Aryl}$), 135.0

($C_{q,Aryl}$), 136.5 ($C_{q,Aryl}$), 136.6 ($C_{q,Aryl}$), 136.9 ($C_{q,Aryl}$), 137.3 ($C_{q,Aryl}$), 137.8 ($C_{q,Aryl}$), 138.3 ($C_{q,Aryl}$), 141.1 ($C_{q,Aryl}$), 142.5 (br, $C_{q,Aryl}$), 143.8 ($C_{q,Aryl}$), 147.3 ($C_{q,Aryl}$), 147.4 ($C_{q,Aryl}$), 147.6 ($C_{q,Aryl}$) ppm.
^{29}Si{^{1}H} NMR (80 MHz, C_6D_6, 298 K): δ = 4.2 ppm. (assigned by ^{1}H/^{29}Si HMBC)
^{119}Sn{^{1}H} NMR (149 MHz, C_6D_6, 298 K): δ = -133.5 ppm.

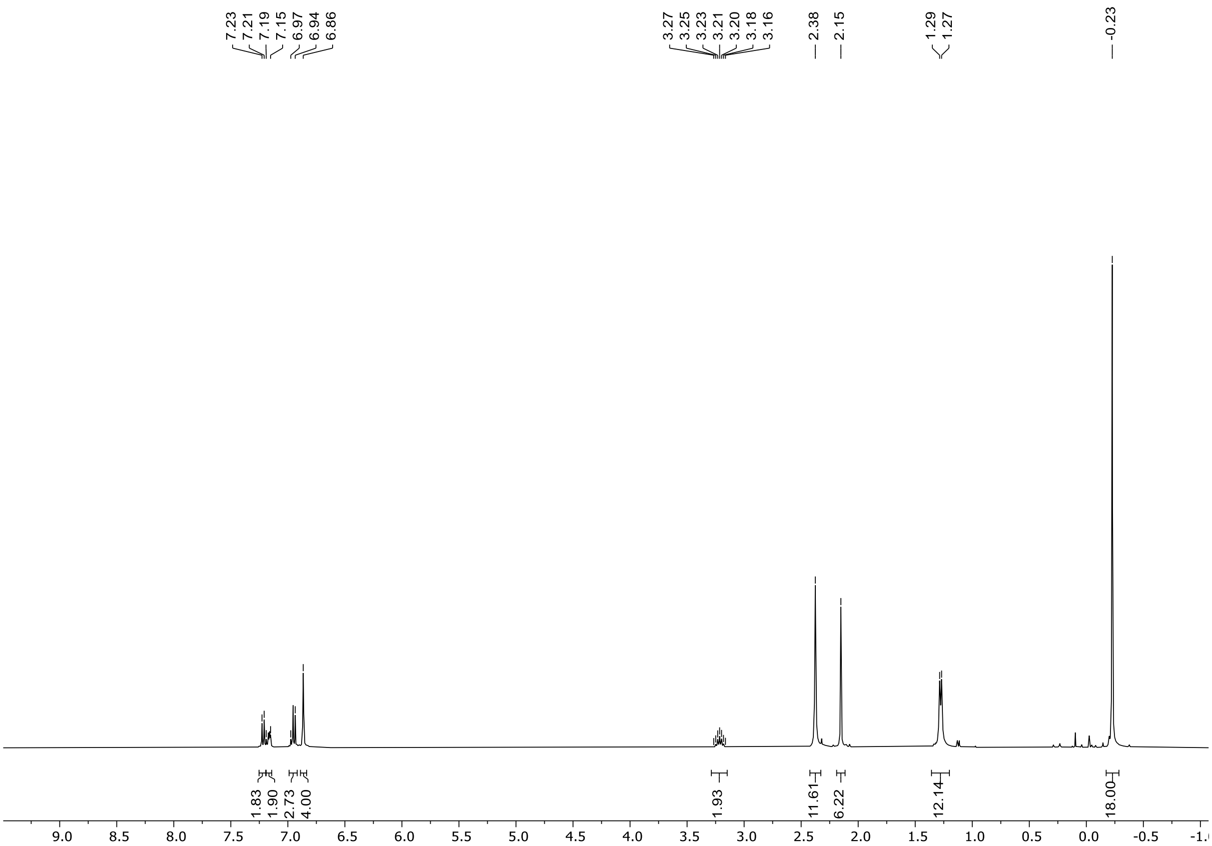

Figure S33. ^{1}H NMR spectrum measured after 5 minutes of ^{Mes}TerSn(N(SiMe$_3$)$_2$)=NDipp (**Sn7b**) (400 MHz, C_6D_6, 298 K).

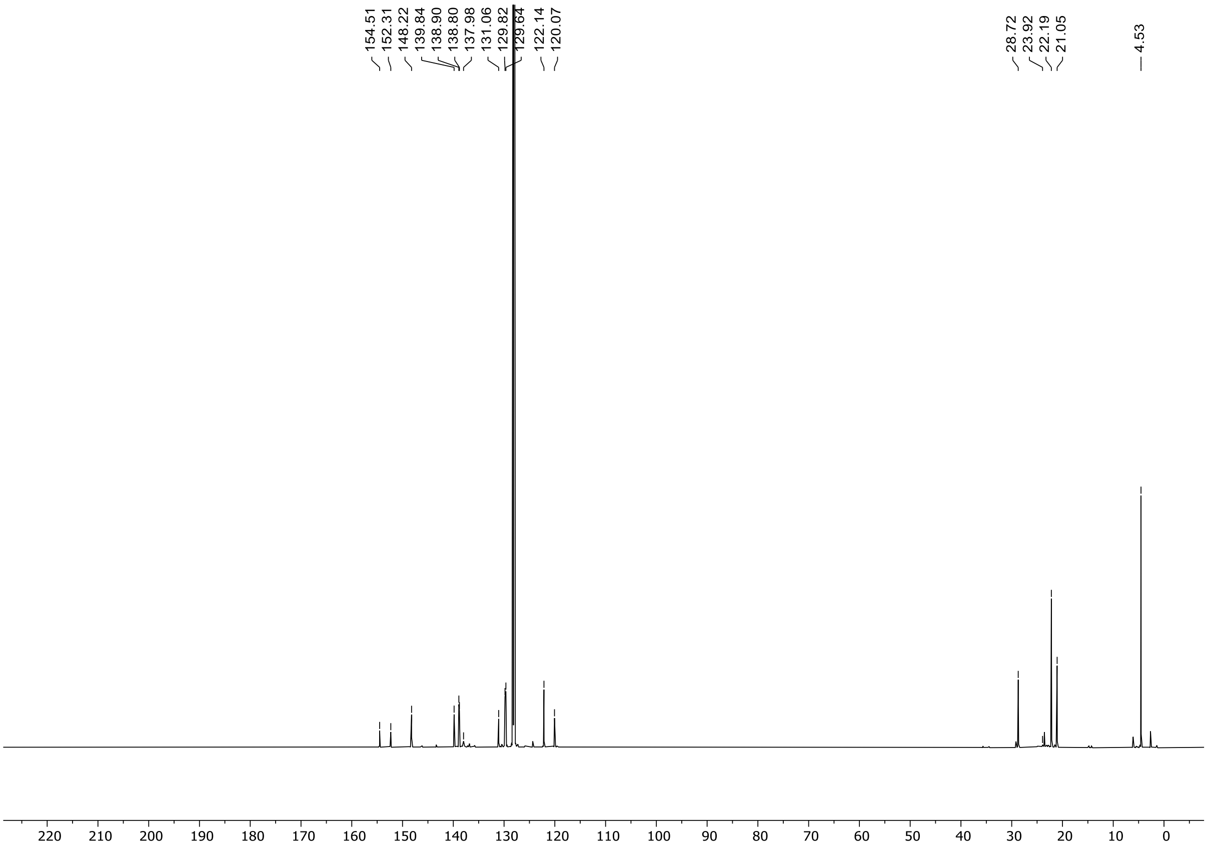

Figure S34. $^{13}C\{^{1}H\}$ NMR spectrum of ^{Mes}TerSn(N(SiMe$_3$)$_3$)=NDipp **Sn7b** (400 MHz, C_6D_6, 298 K); 1.4 ppm: silicon grease.

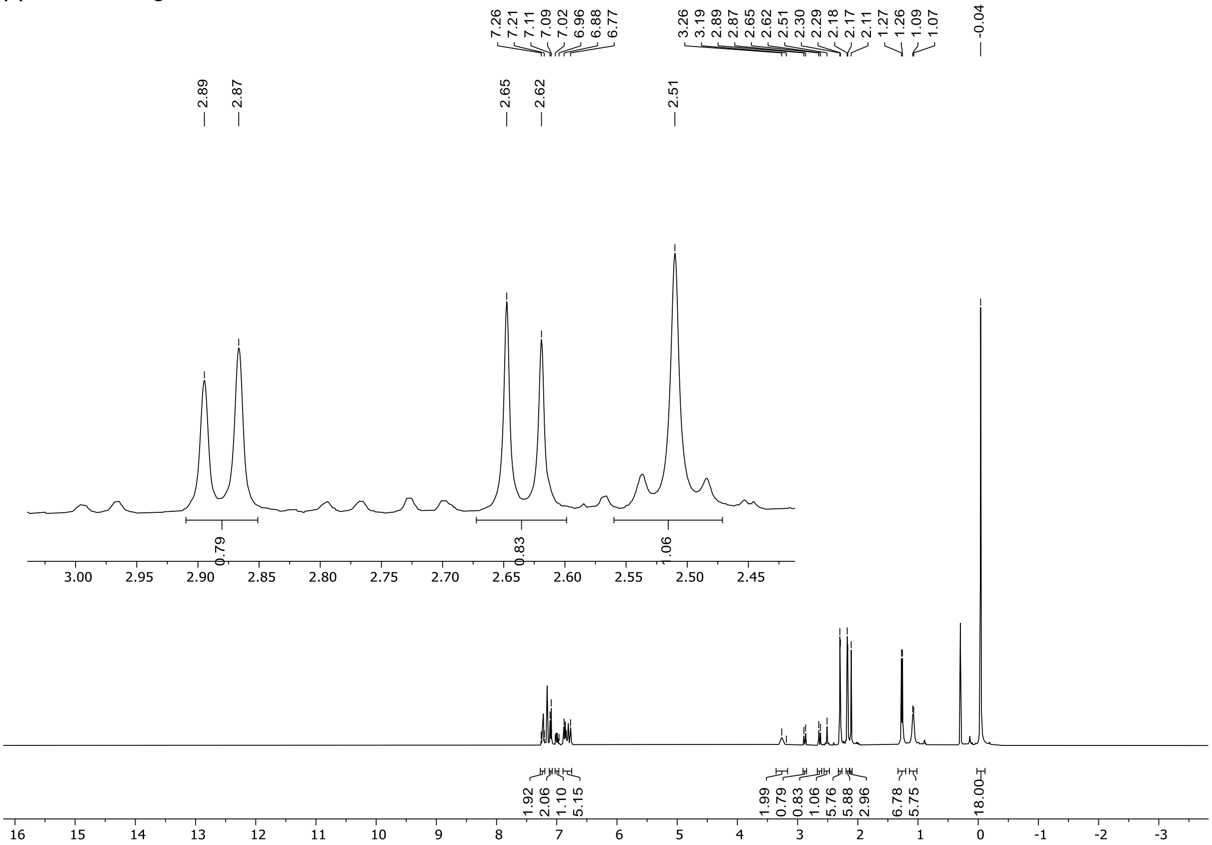

Figure S35. ^{1}H NMR spectrum of the C–H-activation product **Sn4c** (400 MHz, C_6D_6, 298 K); 0.29 ppm: silicon grease.

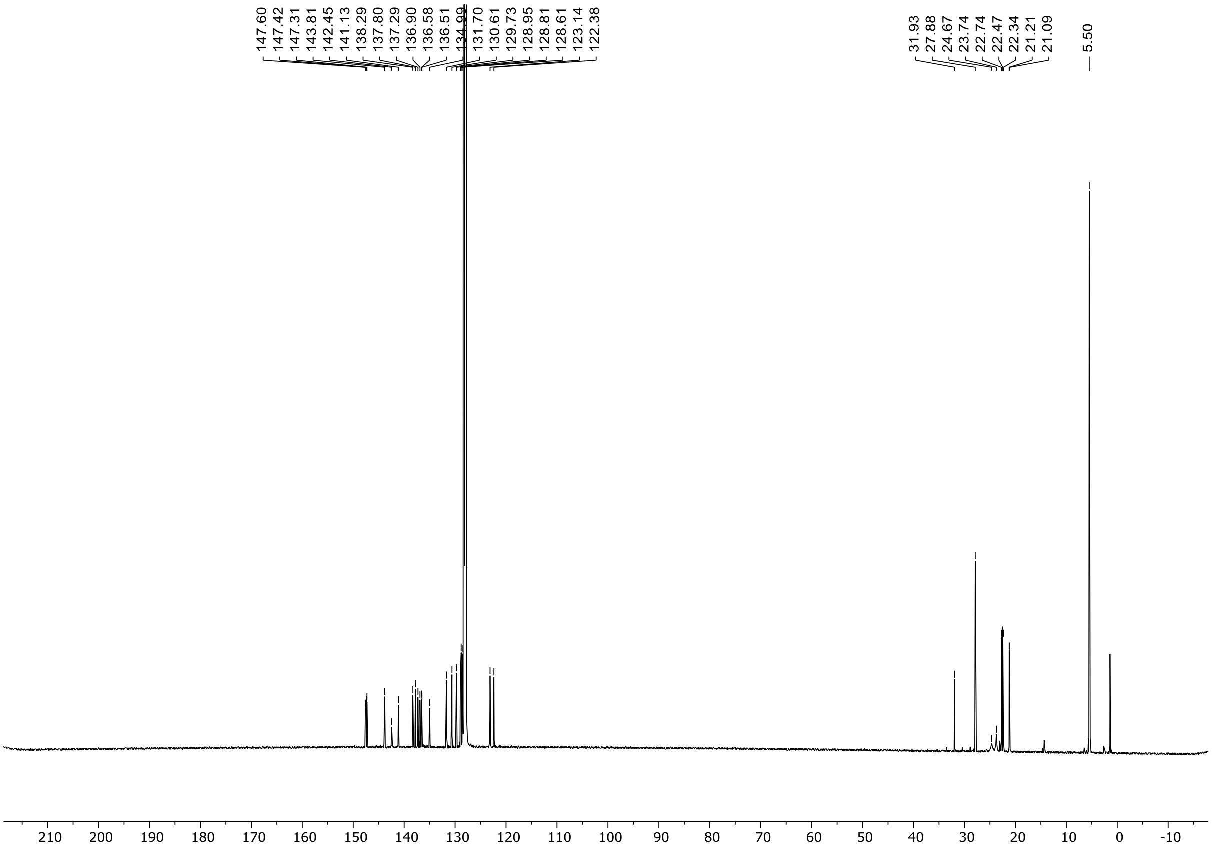

Figure S36. $^{13}C\{^1H\}$ NMR spectrum of the C–H-activation product **Sn4c** (400 MHz, C_6D_6, 298 K); 1.4 ppm: silicon grease.

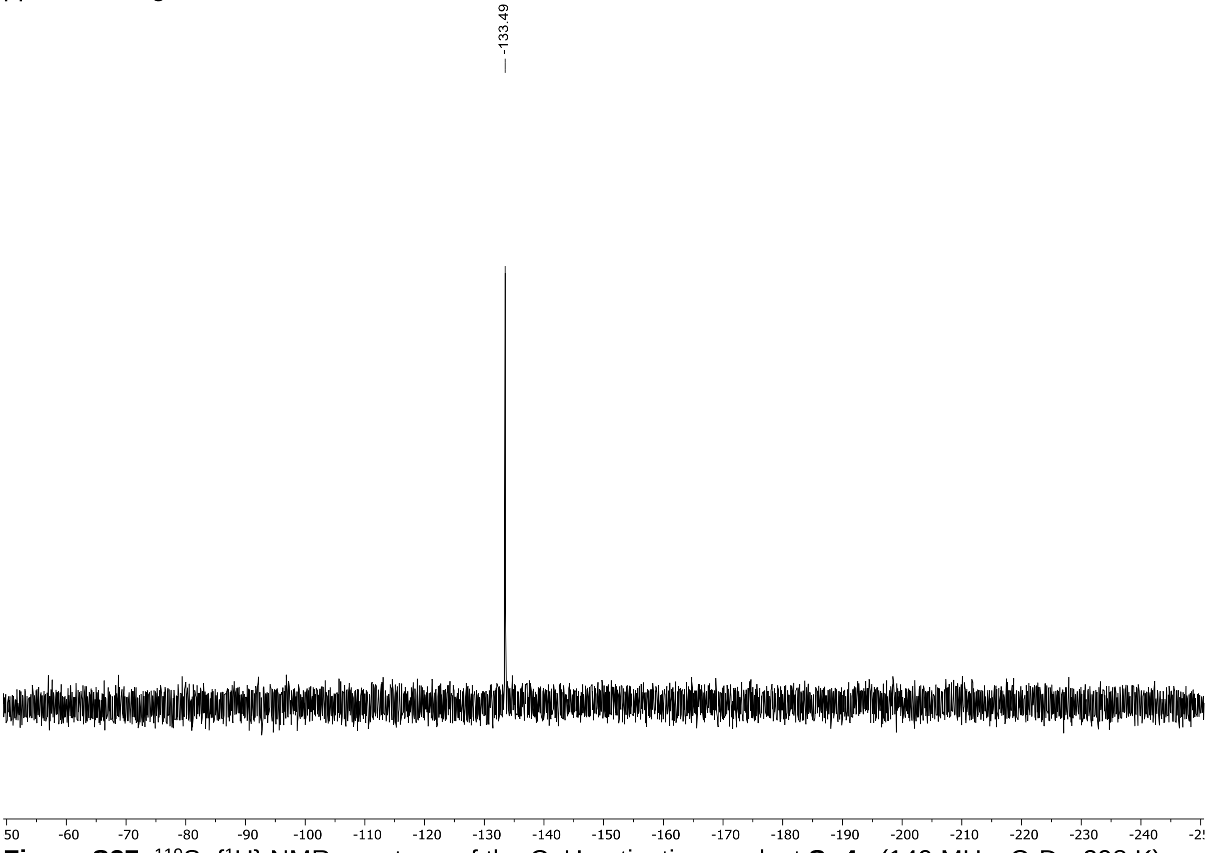

Figure S37. $^{119}Sn\{^1H\}$ NMR spectrum of the C–H-activation product **Sn4c** (149 MHz, C_6D_6, 298 K).

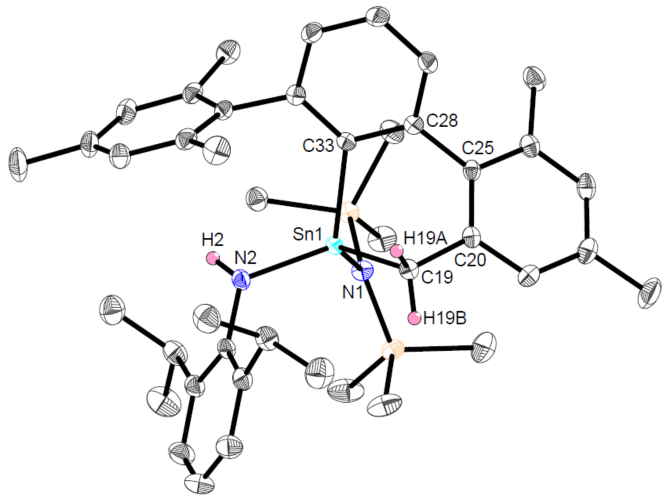

Figure S38. Molecular structure of C–H activation product **Sn4c** in the crystal. Thermal ellipsoids are drawn at the 50% probability level (hydrogen atoms (except H2, H19A and H19B) have been omitted for clarity). Selected bond lengths (Å) and angles (deg): Sn1–N1 2.0648(12), Sn1–N2 2.0521(13), Sn1–C19 2.1465(14), Sn1–C33 2.1557(14), C19–C20 1.5087(19), C20–C25 1.414(2), C25–C28 1.5057(19), C28–C33 1.4190(19), N1–Sn1–N2 112.11(5), N1–Sn1–C19 113.08(5).

Attempted Reaction of $^{Mes}TerSnN(SiMe_3)_2$ (Sn1) with $N_3{}^{Mes}Ter$ (A4)

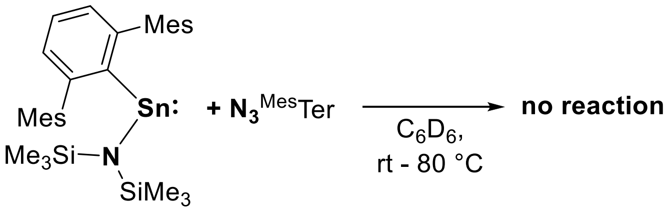

To a solution of $^{Mes}TerSnN(SiMe_3)_2$ (**Sn1**) (0.030 g, 0.051 mmol) in 0.3 mL of C_6D_6 was added a solution of $N_3{}^{Mes}Ter$ (**A4**) (0.018 g, 0.051 mmol) in 0.3 mL of C_6D_6. Since subsequent NMR analysis revealed no reaction, the reaction mixture was heated to 80 °C for 24 h which also did not result in any reaction between both reactants. Shown below is the ^{1}H NMR spectrum of pure $N_3{}^{Mes}Ter$ (**A4**) and the obtained ^{1}H NMR spectrum of the reaction mixture after 24 h at 80 °C.

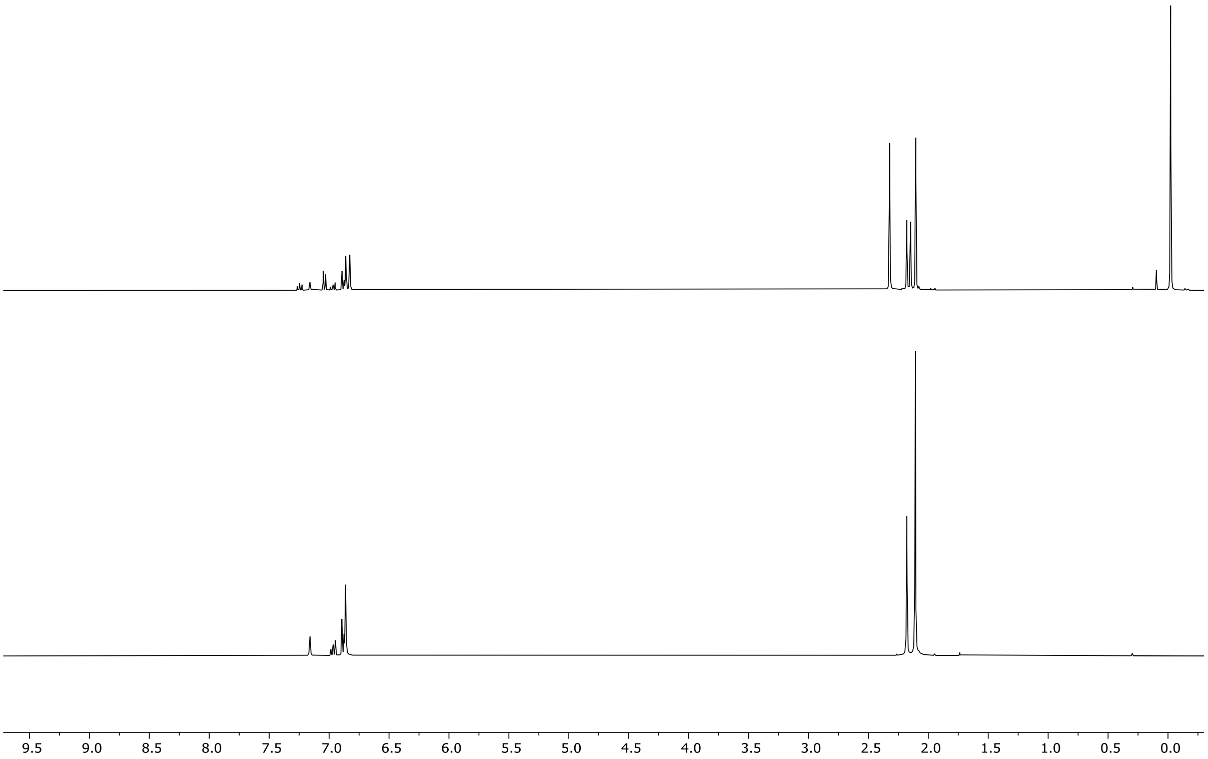

Figure S39. ^{1}H NMR spectrum of $N_3{}^{Mes}Ter$ (**A4**) (bottom) and after the addition of $^{Mes}TerSnN(SiMe_3)_2$ (**Sn1**) after 24 h at 80 °C (400 MHz, C_6D_6, 298 K).

Synthesis of ^{Mes}TerSn(hmds)=NQuin (Sn8) and of the C–H-Activation Product Sn4d

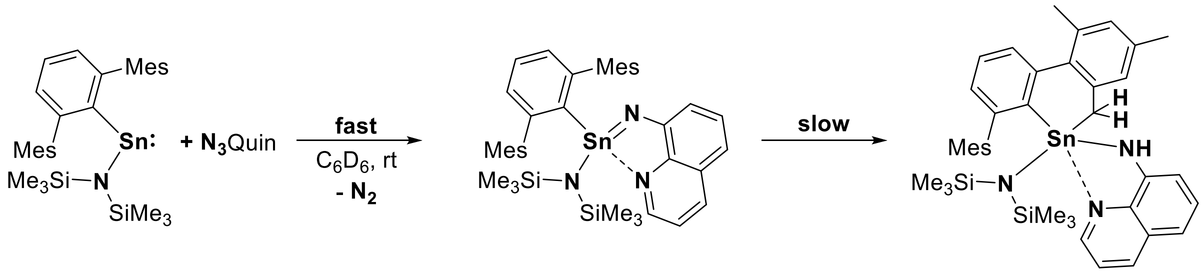

To a solution of ^{Mes}TerSnN(SiMe$_3$)$_2$ (**Sn1**) (0.025 g, 0.042 mmol) in 0.3 mL of C_6D_6 was added a solution of N$_3$Quin (**A5**) (0.007 g, 0.042 mmol) in 0.3 mL of C_6D_6. The reaction immediately results in gas evolution and a colour change to dark green. Subsequent NMR analysis (Figure S40) reveals overall clean conversion to a single product. All volatile components were removed under vacuum and the residue was suspended in 0.5 mL of *n*-hexane, filtered and stored at -30 °C to give dark green crystals suitable for single crystal X-ray diffraction, confirming that the initial product of the reaction is ^{Mes}TerSn(N(SiMe$_3$)$_2$)=NQuin (**Sn8**).
In solution ^{Mes}TerSn(N(SiMe$_3$)$_2$)=NQuin (**Sn8**) reacts to another single product and this reaction progress was monitored by ^{1}H NMR spectroscopy (Figure S37). After the reaction is finished all volatile components were removed under vacuum and the remaining solid was suspended in 0.5 mL of *n*-heptane. Filtration and subsequent storage of the bright yellow solution at -30 °C yields the intramolecular C–H activation product **Sn4d** as a crystalline yellow solid. These crystals were suitable for single crystal X-ray diffraction. The remaining small amount of crystals (approx. 5 mg) was used for multinuclear NMR spectroscopy.
During one attempt to obtain crystals of **Sn4d** suitable for single crystal X-ray diffraction at higher temperatures (>80 °C) a small amount of red crystals was obtained suitable for single crystal X-ray diffraction and were verified to be the dimeric complex **Sn9** shown in Figure S44.

Note: ^{Mes}TerSn(N(SiMe$_3$)$_2$)=NQuin (**Sn8**) in the solid state slowly undergoes intramolecular C–H-activation to give **Sn4d**. Due to this behaviour, **Sn8** was synthesized *in situ* and directly reacted further for the reactivity studies.

Characteristic NMR data of ^{Mes}TerSn(N(SiMe$_3$)$_2$)=NQuin (Sn8):
^{1}H NMR (400 MHz, C_6D_6, 298 K): δ = -0.03 (s(br), 18H, Si(CH$_3$)$_3$), 1.99 (s(br), 6H, CH$_3$), 2.21 (s, 6H, CH$_3$), 2.43 (s(br), 6H, CH$_3$), 6.17-6.19 (m, 1H, CH$_{Aryl}$), 6.26-6.29 (m, 1H, CH$_{Aryl}$), 6.61-6.82(m(br), 3H, CH$_{Aryl}$), 6.87-6.92 (m, 3H, CH$_{Aryl}$), 7.09-7.14 (m, 2H, CH$_{Aryl}$), 7.34-7.42 (m, 2H, CH$_{Aryl}$), 7.51-7.53 (m, 1H, CH$_{Aryl}$) ppm.

Analytical data of C–H-activation product Sn4d:
^{1}H NMR (400 MHz, C_6D_6, 298 K): δ = -0.11 (s(br), 9H, Si(CH$_3$)$_3$), 0.12 (s(br), 9H, Si(CH$_3$)$_3$), 1.73 (s, 3H, CH$_3$), 1.95-1.96 (m, 6H, CH$_3$), 2.18 (d, $^3J_{H,H}$ = 10.1 Hz, 1H, CH$_2$), 2.31 (s, 3H, CH$_3$), 2.44-2.46 (m, 4H, CH$_3$, CH$_2$), 3.27 (s, 1H, NH), 5.74 (s(br), 1H, CH$_{Aryl}$), 5.89 (s, 1H, CH$_{Aryl}$), 6.26-6.27 (m, 1H, CH$_{Aryl}$), 6.51-6.52 (m, 1H, CH$_{Aryl}$), 6.61-6.63 (m, 1H, CH$_{Aryl}$), 6.68-7.00 (m, 1H, CH$_{Aryl}$), 7.01-7.03 (m, 2H, CH$_{Aryl}$), 7.08-7.11 (m, 1H, CH$_{Aryl}$), 7.21-7.23 (m, 1H, CH$_{Aryl}$), 7.27-7.29 (m, 1H, CH$_{Aryl}$), 7.38-7.40 (m, 1H, CH$_{Aryl}$), 8.37 (s, 1H, CH$_{Aryl}$) ppm.
^{13}C{^{1}H} NMR (101 MHz, C_6D_6, 298 K): δ = 4.6 (br, Si(CH$_3$)$_3$), 5.9 (br, Si(CH$_3$)$_3$), 21.1 (CH$_3$), 21.3 (CH$_3$), 21.5 (CH$_3$), 22.0 (CH$_3$), 23.1 (CH$_3$), 32.3 (CH$_2$), 109.7 (CH$_{Aryl}$), 112.2 (CH$_{Aryl}$), 120.7 (CH$_{Aryl}$), 127.58 (CH$_{Aryl}$),* 127.61 (CH$_{Aryl}$)*, 128.81 (CH$_{Aryl}$), 128.83 (CH$_{Aryl}$), 129.1 (CH$_{Aryl}$), 129.2 (CH$_{Aryl}$), 129.6 (CH$_{Aryl}$), 132.5 (CH$_{Aryl}$), 134.3 (C$_{q,Aryl}$), 134.5 (C$_{q,Aryl}$), 134.6 (C$_{q,Aryl}$), 135.7 (C$_{q,Aryl}$), 136.0 (C$_{q,Aryl}$), 136.4 (C$_{q,Aryl}$), 136.6 (CH$_{Aryl}$), 137.4 (C$_{q,Aryl}$), 140.0 (C$_{q,Aryl}$), 141.6 (C$_{q,Aryl}$), 142.8 (CH$_{Aryl}$), 146.3 (C$_{q,Aryl}$), 146.9 (C$_{q,Aryl}$), 147.9 (C$_{q,Aryl}$), 150.9 (C$_{q,Aryl}$) ppm.
* = overlap with C_6D_6 signal (assigned by ^{1}H/^{13}C HSCQC)
^{119}Sn{^{1}H} NMR (149 MHz, C_6D_6, 298 K): not observed.

EA: Anal. calcd. for $C_{39}H_{49}N_3Si_2Sn$: C, 63.76; H, 6.72; N, 5.72; Found: C, 64.46; H, 6.78; N, 5.63.

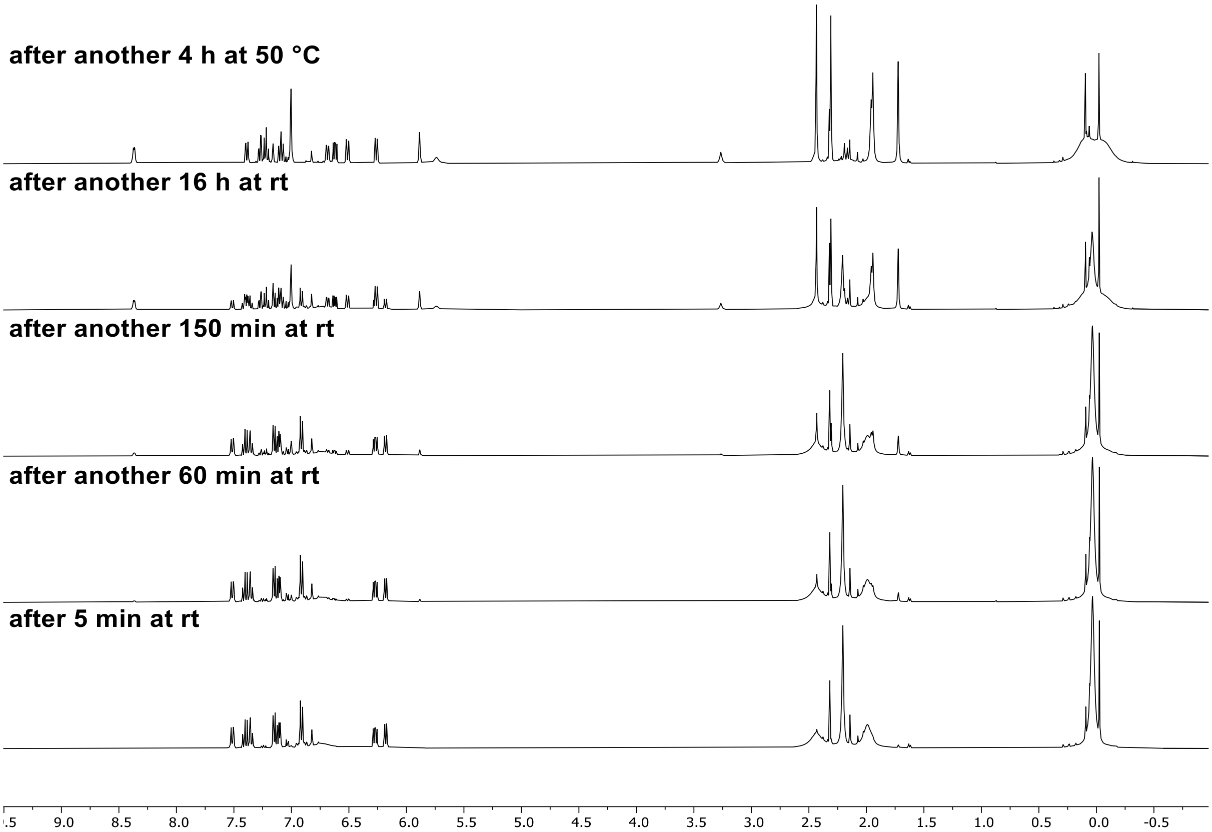

Figure S40. Monitoring of the reaction of ^{Mes}TerSnN(SiMe$_3$)$_2$ (**Sn1**) with N_3Quin (**A5**) via ^{1}H NMR spectroscopy (400 MHz, C_6D_6, 298 K).

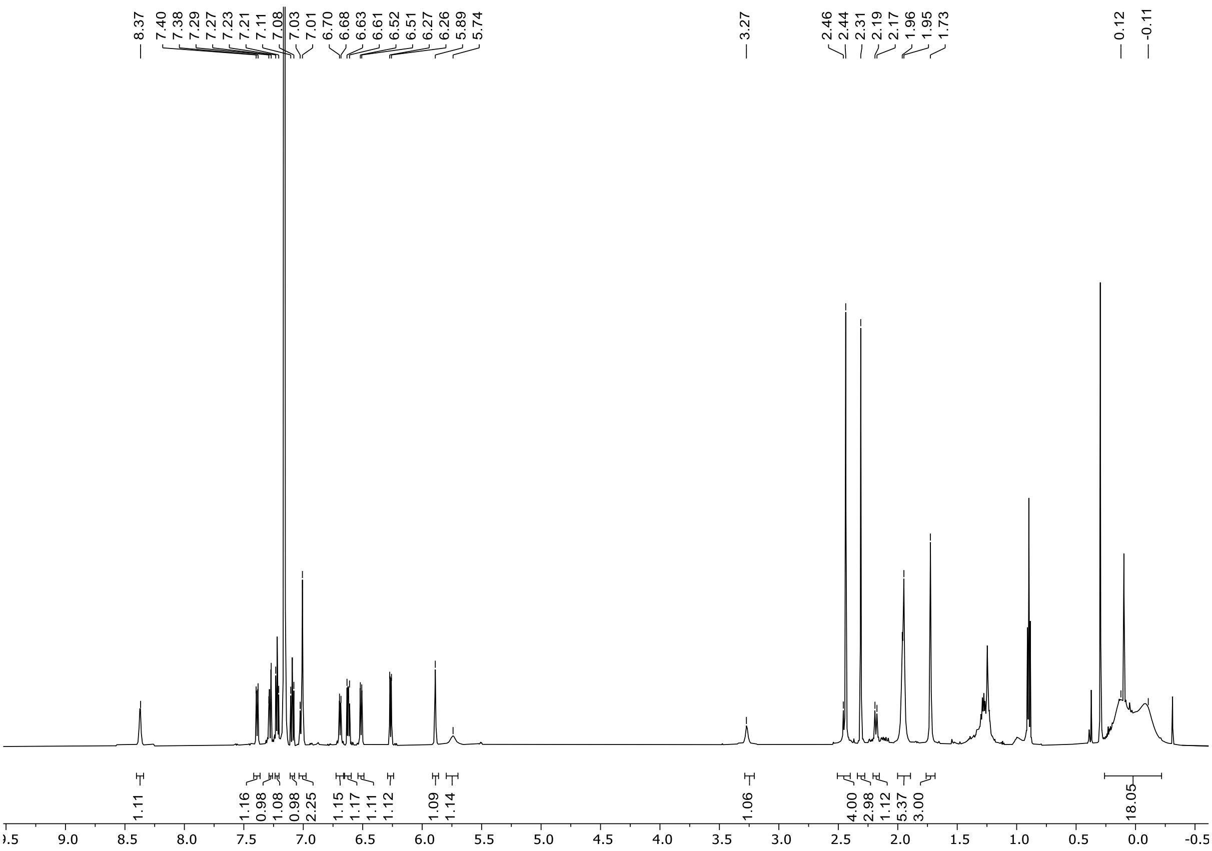

Figure S41. ^{1}H NMR spectrum of the C–H-activation product **Sn4d** (400 MHz, C_6D_6, 298 K); 0.29 ppm: silicon grease; 0.90, 1.24-1.40 ppm: *n*-heptane.

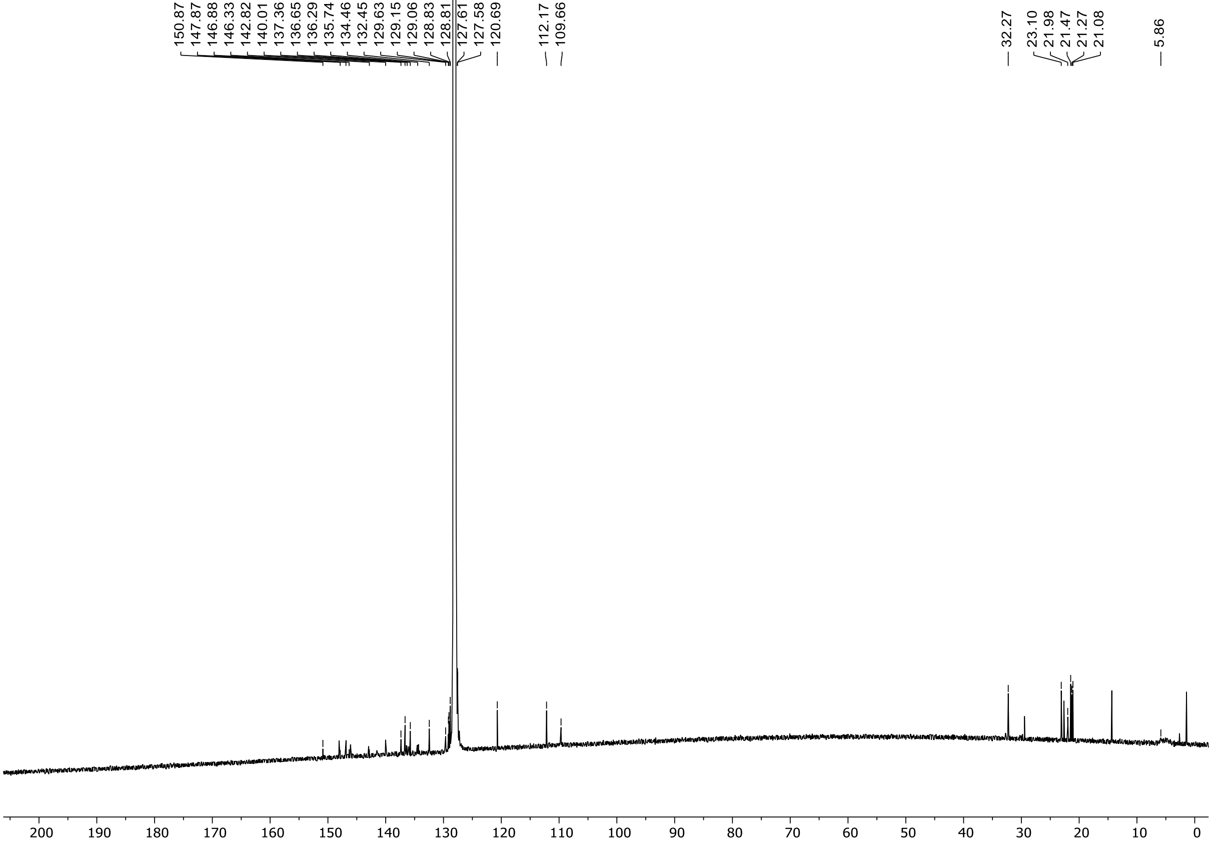

Figure S42. ^{13}C{^{1}H} NMR spectrum of the C–H-activation product **Sn4d** (400 MHz, C_6D_6, 298 K); 1.4 ppm: silicon grease, 14.4, 22.6, 29.5, 32.7 ppm: *n*-heptane.

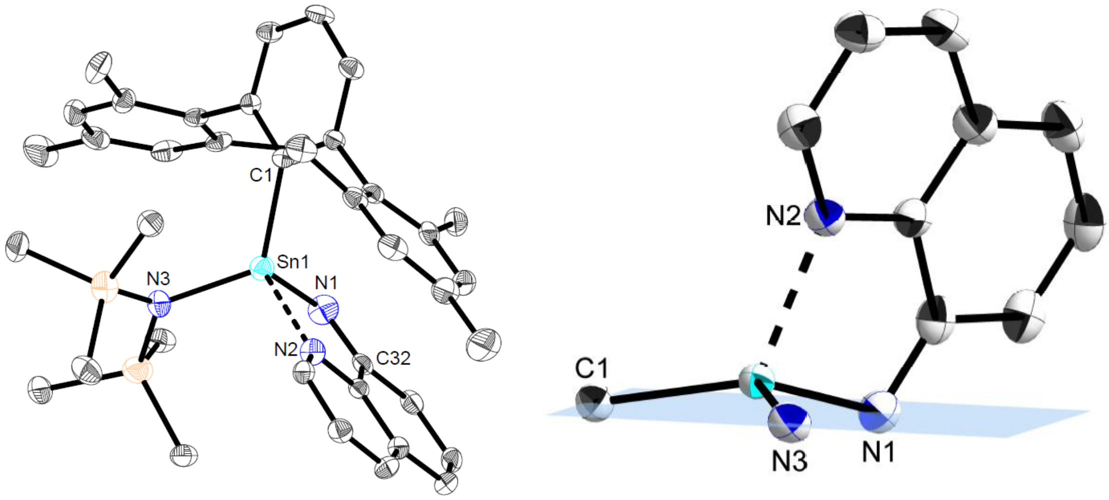

Figure S43. Molecular structure of ^{Mes}TerSnN(N(SiMe$_3$)$_2$)=NQuin (**Sn8**) in the crystal. Thermal ellipsoids are drawn at the 50% probability level (hydrogen atoms have been omitted for clarity). Selected bond lengths (Å) and angles (deg): Sn1–N1 2.022(4), Sn1–N2 2.198(3), Sn1–N3 2.032(3), Sn1–C1 2.158(4), N1–Sn1–N2 93.09(13), N1–Sn1–N3 114.71(14).

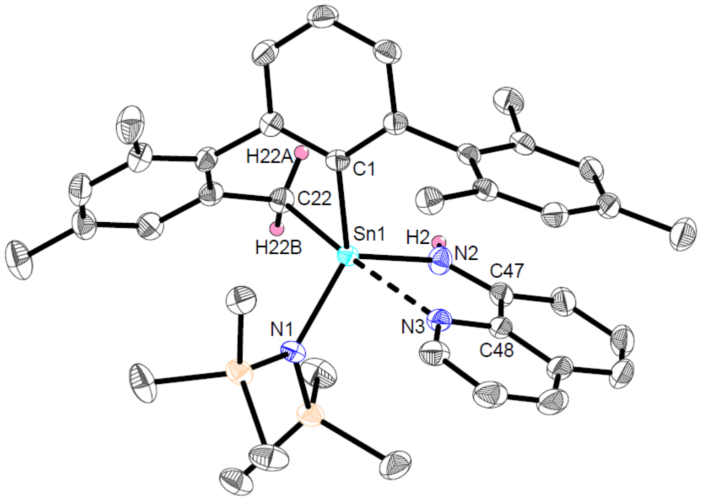

Figure S44. Molecular structure of the C–H activation product **Sn4d** in the crystal. Thermal ellipsoids are drawn at the 50% probability level (hydrogen atoms except H2, H22A and H22B have been omitted for clarity). Selected bond lengths (Å) and angles (deg): Sn1–N1 2.0717(12), Sn1–N2 2.0572(13), Sn1–N3 2.4601(13), Sn1–C22 2.1843(15), Sn1–C1 2.1746(14), N1–Sn1–C1 128.14(5), N2–Sn1–N3 71.26(5).

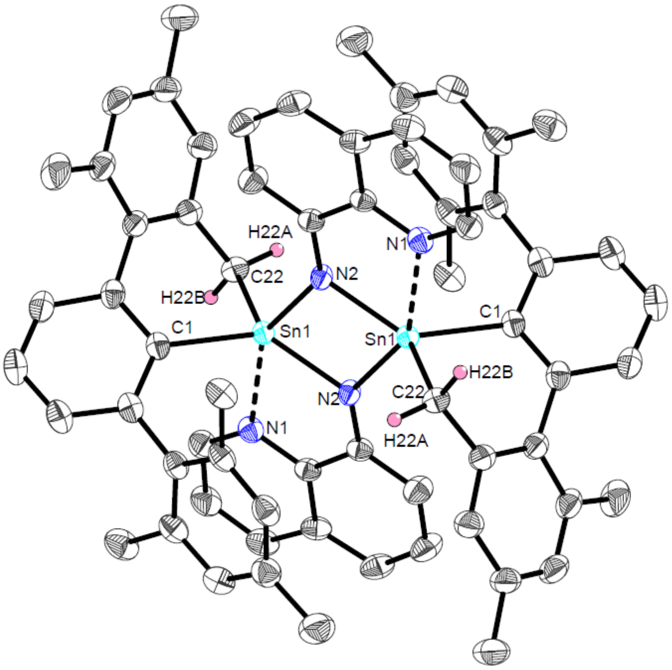

Figure S45. Molecular structure of the dimer **Sn9** in the crystal. Thermal ellipsoids are drawn at the 50% probability level (hydrogen atoms except H22A and H22B have been omitted for clarity). Selected bond lengths (Å) and angles (deg): Sn1–N1 2.4351(17), Sn1–N2 2.0617(15), Sn1–C22 2.1651(19), Sn1–C1 2.1497(19), C1–Sn1–N2 113.07(7), N2–Sn1–N2' 76.82(7), Sn1–N2–Sn1'103.18(7).

Reaction of $^{Mes}TerSn(N(SiMe_3)_2)=NDipp$ (Sn7b) with HCCPh

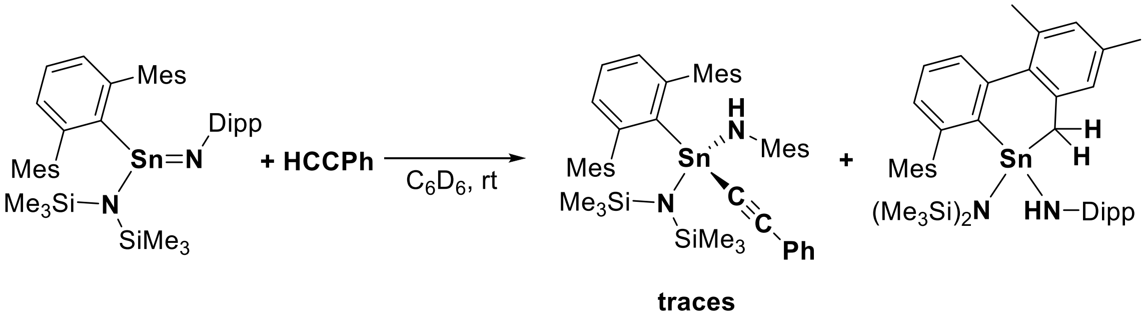

To a solution of $^{Mes}TerSnN(SiMe_3)_2$ (**Sn1**) (0.032 g, 0.054 mmol) in 0.3 mL of C_6D_6 was added a solution of N_3Dipp (**A3**) (0.011 g, 0.054 mmol) in 0.3 mL of C_6D_6 for the *in situ* generation of $^{Mes}TerSn(N(SiMe_3)_2)=NDipp$ (**Sn7b**). After the gas evolution has stopped, phenylacetylene (0.006 g, 0.054 mmol) was added and the reaction progress was monitored by ^1H NMR spectroscopy (Figure S45), showing that both the intramolecular C–H activation product **Sn4c** and the HCCPh 1,2-addition product are formed during the reaction in approximately 1:1 ratio.

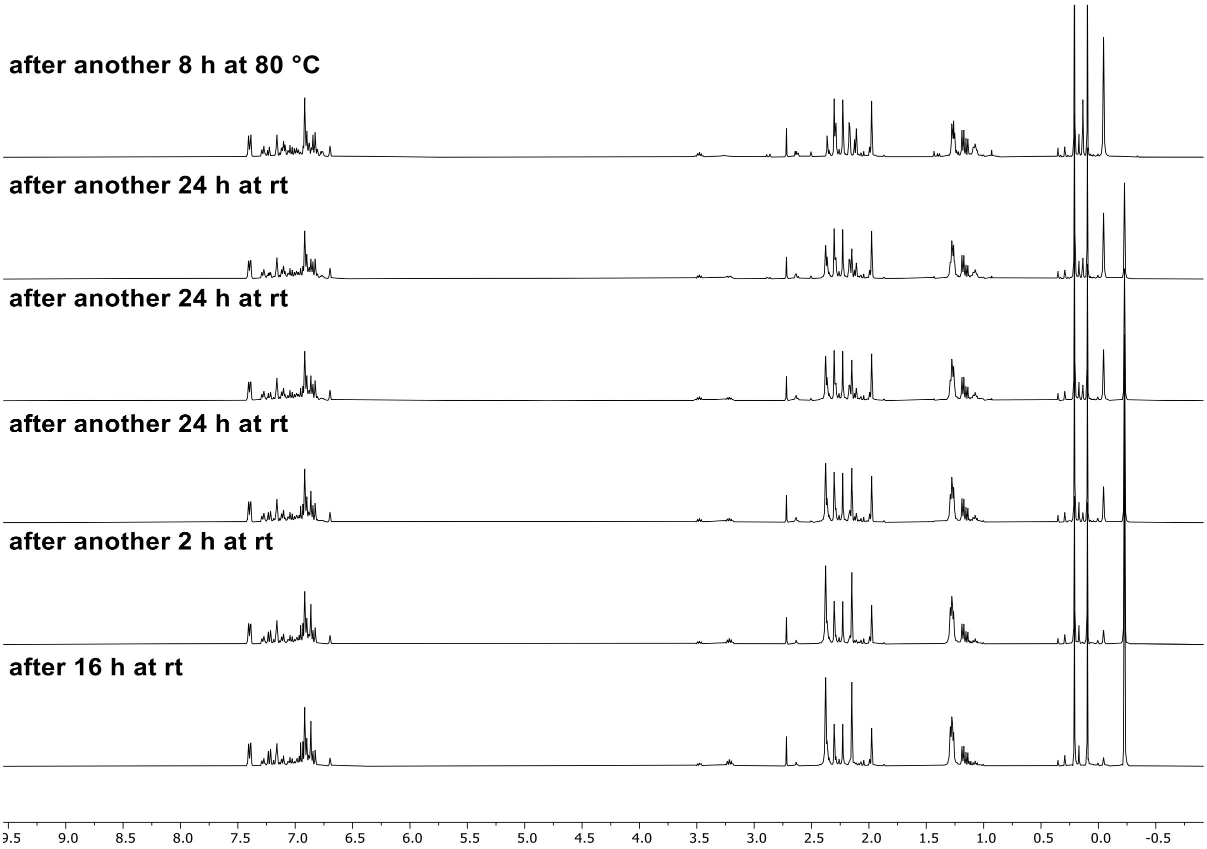

Figure S46. Monitoring of the reaction of $^{Mes}TerSn(N(SiMe_3)_2)=NDipp$ (**Sn7b**) with HCCPh via ^1H NMR spectroscopy (400 MHz, C_6D_6, 298 K); 1.29-1.31 and 3.48 ppm: excess N_3Dipp (**A3**).

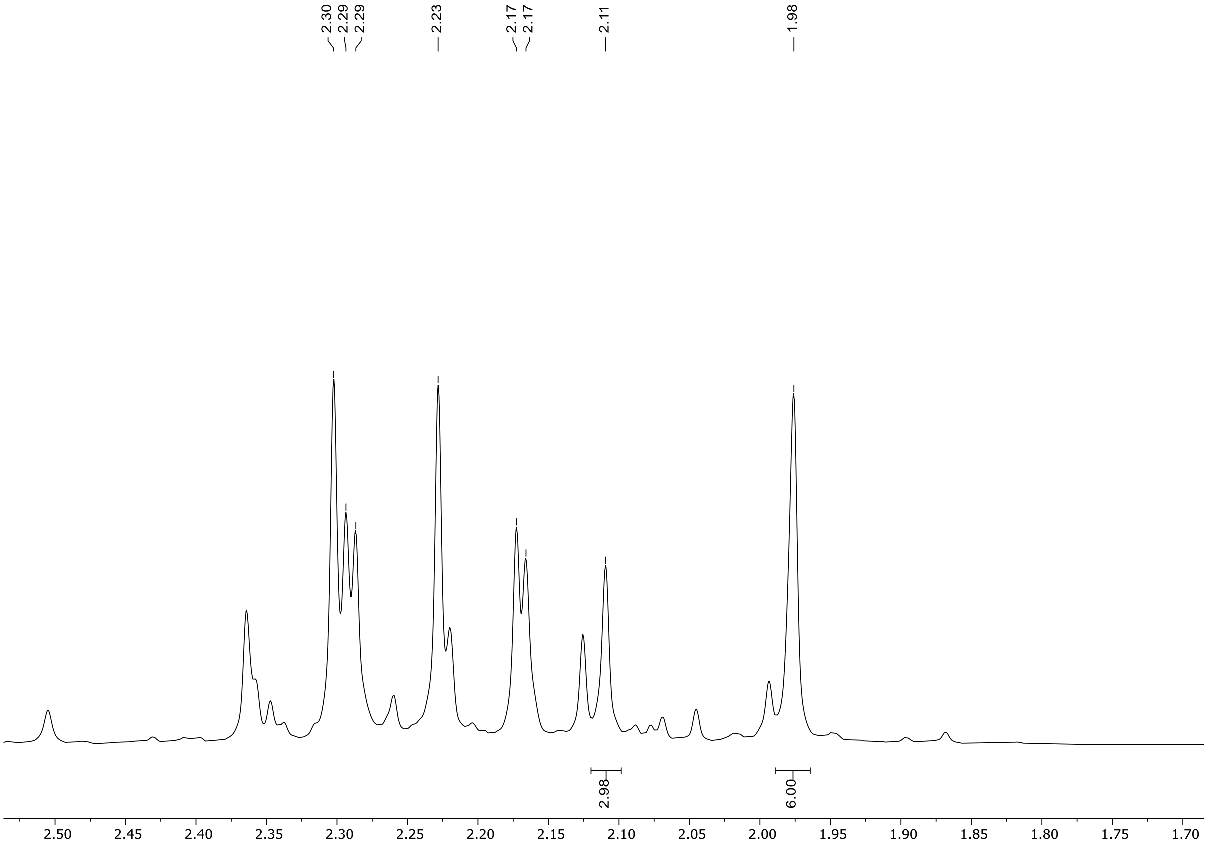

Figure S47. Excerpt of the ^{1}H NMR spectrum after full consumption of ^{Mes}TerSn(N(SiMe$_3$)$_2$)=NDipp (**Sn7b**) (400 MHz, C_6D_6, 298 K); 2.11, 2.17, 2.17, 2.29 and 2.29 ppm: Intramolecular C–H activation product **Sn4c**; 1.98, 2.23 and 2.30 ppm: 1,2-addition product.

Synthesis of ^{Mes}TerSn(N(SiMe$_3$)$_2$)(CCPh)NHMes (Sn10a)

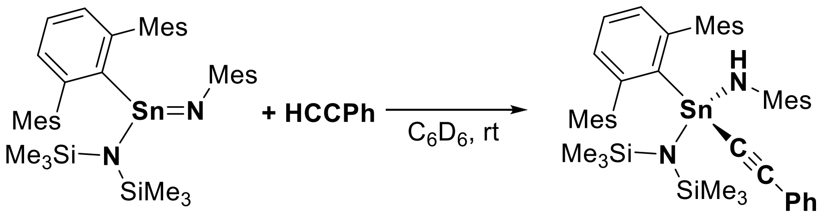

To a solution of ^{Mes}TerSnN(SiMe$_3$)$_2$ (**Sn1**) (0.050 g, 0.084 mmol) in 0.3 mL of C_6D_6 was added a solution of N$_3$Mes (**A2**) (0.014 g, 0.084 mmol) in 0.3 mL of C_6D_6 for the *in situ* generation of ^{Mes}TerSn(N(SiMe$_3$)$_2$)=NMes (**Sn7a**). After the gas evolution has stopped, phenylacetylene (0.009 g, 0.084 mmol) was added which results in a colour change to a bright yellow-orange. All volatiles were removed under vacuum and 0.6 mL of *n*-hexane were added to the solid followed by filtration and subsequent storage of the saturated solution at 4 °C to give ^{Mes}TerSn(N(SiMe$_3$)$_2$)(CCPh)NHMes (**Sn10a**) as colourless crystals. Crystals obtained this way were suitable for single crystal X-ray diffraction.

Yield: 0.035 g (0.042 mmol; 50%).
^{1}H NMR (400 MHz, C_6D_6, 298 K): δ = -0.19 (s, 18H, Si(CH$_3$)$_3$), 2.05 (s, 6H, CH$_3$), 2.18 (s, 3H, CH$_3$), 2.26 (s, 6H, CH$_3$), 2.33-2.34 (m, 7H, NH, CH$_3$), 2.37 (s, 6H, CH$_3$), 6.78-6.79 (m, 4H, CH$_{Aryl}$), 6.80-6.90 (m, 4H, CH$_{Aryl}$), 6.97-7.07 (m, 3H, CH$_{Aryl}$), 7.11-7.15 (m, 1H, CH$_{Aryl}$), 7.36-7.38 (m, 2H, CH$_{Aryl}$) ppm.
^{13}C{^{1}H} NMR (101 MHz, C_6D_6, 298 K): δ = 6.4 (Si(CH$_3$)$_3$), 20.7 (CH$_3$), 21.0 (CH$_3$), 21.8 (CH$_3$), 22.3 (CH$_3$), 22.6 (CH$_3$), 101.4 (Sn satellites: $^{1}J_{119Sn,C}$ = 785.8 Hz, $^{1}J_{117Sn,C}$ = 751.0 Hz, Sn$\underline{C}_q$C$_q$Ph), 108.8 (Sn satellites: $^{2}J_{119Sn,C}$ = 155.3 Hz, $^{2}J_{117Sn,C}$ = 149.0 Hz, SnC$_q\underline{C}_q$Ph), 124.7 (C$_{q,Aryl}$), 128.0 (CH$_{Aryl}$)*, 128.47 (CH$_{Aryl}$), 128.51 (CH$_{Aryl}$), 129.4 (CH$_{Aryl}$), 129.5 (C$_{q,Aryl}$), 129.6 (CH$_{Aryl}$), 129.7 (CH$_{Aryl}$), 130.3 (CH$_{Aryl}$), 130.9 (CH$_{Aryl}$), 131.7 (CH$_{Aryl}$), 137.1 (C$_{q,Aryl}$), 137.4 (C$_{q,Aryl}$), 137.8 (C$_{q,Aryl}$), 140.1 (C$_{q,Aryl}$), 143.0 (C$_{q,Aryl}$), 144.5 (C$_{q,Aryl}$), 149.2 (C$_{q,Aryl}$) ppm.
* = overlap with C_6D_6 signal (assigned by ^{1}H/^{13}C HSCQC)
^{29}Si{^{1}H} NMR (80 MHz, C_6D_6, 298 K): δ = 5.9 ppm. (assigned by ^{1}H/^{29}Si HMBC)
^{119}Sn{^{1}H} NMR (149 MHz, C_6D_6, 298 K): δ = -199.6 ppm.
EA: Anal. calcd. for $C_{47}H_{60}N_2Si_2Sn$: C, 68.19; H, 7.31; N, 3.38; Found: C, 67.16; H, 7.55; N, 3.19.

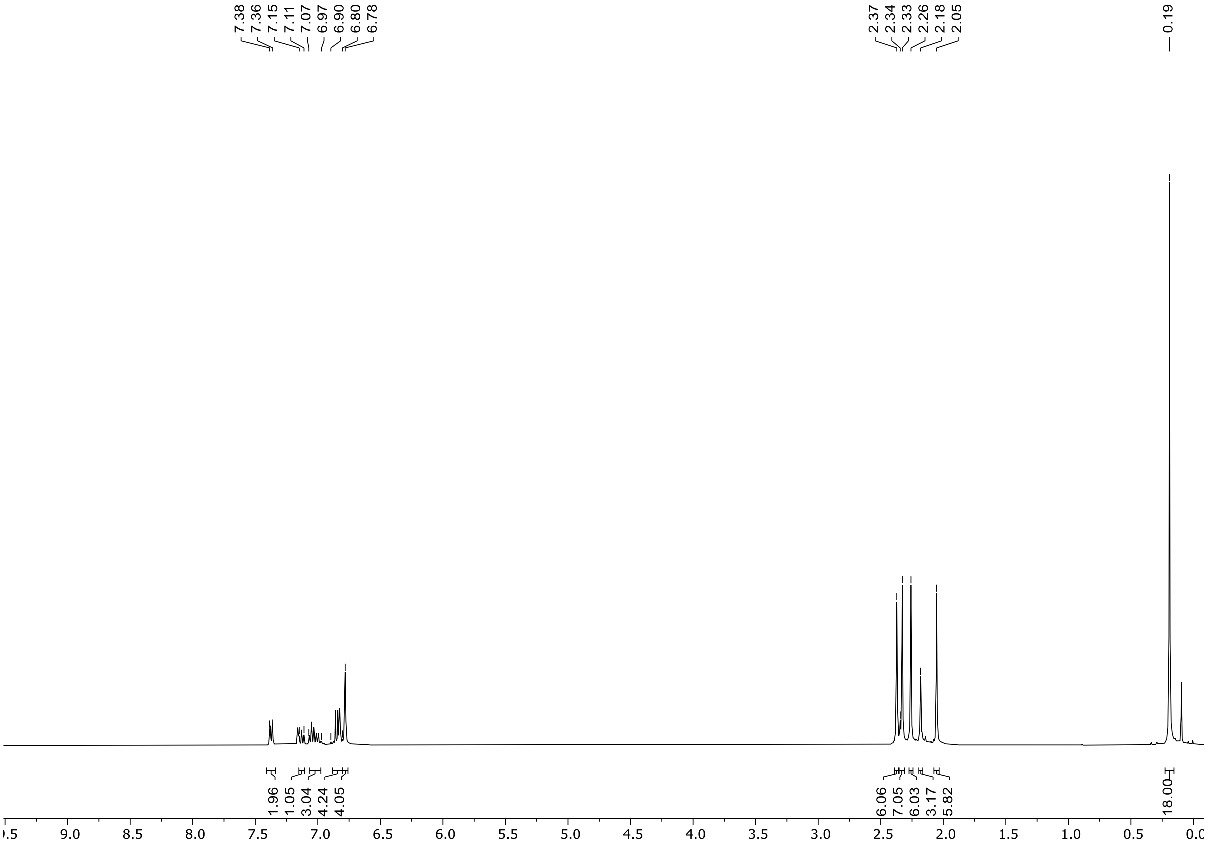

Figure S48. ^{1}H NMR spectrum measured of ^{Mes}TerSn(N(SiMe$_3$)$_2$)(CCPh)NHMes (**Sn10a**) (400 MHz, C_6D_6, 298 K).

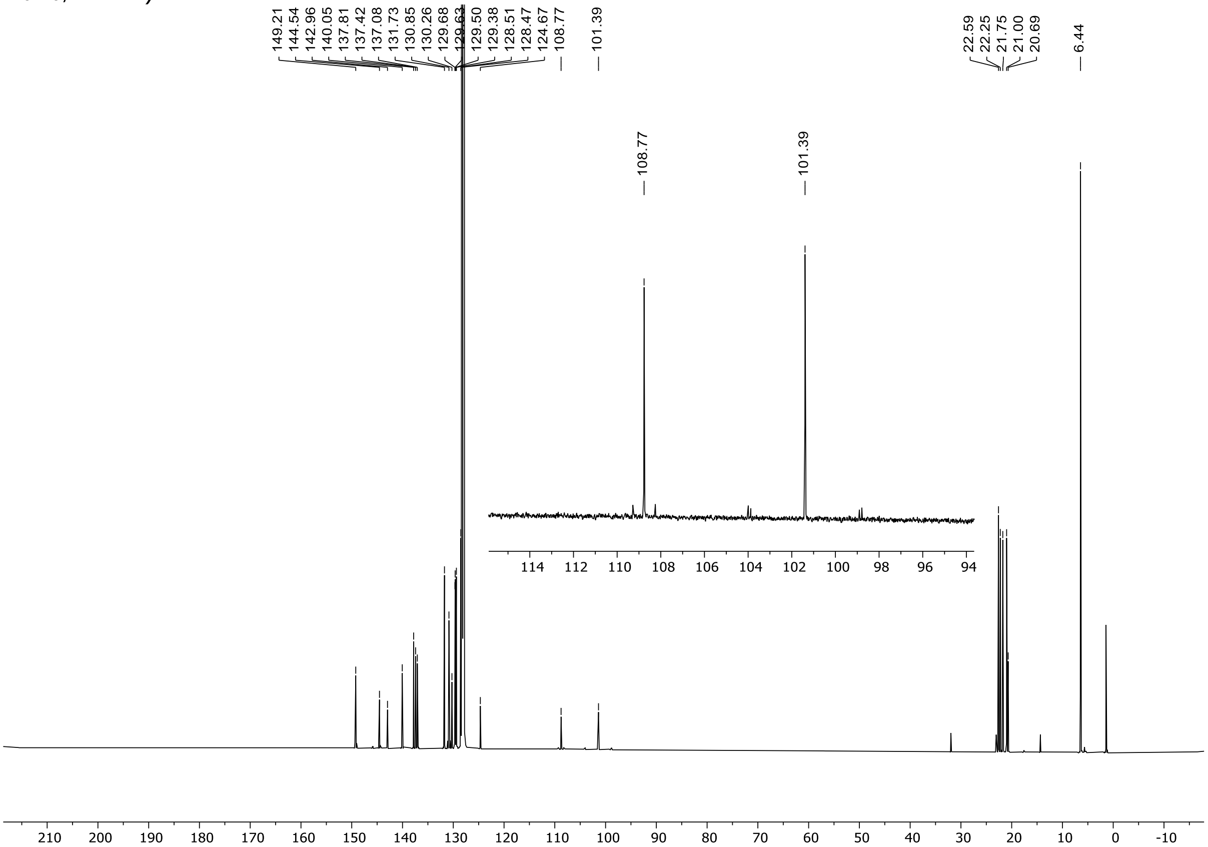

Figure S49. ^{13}C{^{1}H} NMR spectrum of ^{Mes}TerSn(N(SiMe$_3$)$_2$)(CCPh)NHMes (**Sn10a**) (400 MHz, C_6D_6, 298 K); 1.4 ppm: silicon grease; 14.3, 23.0, 32.0 ppm: *n*-hexane.

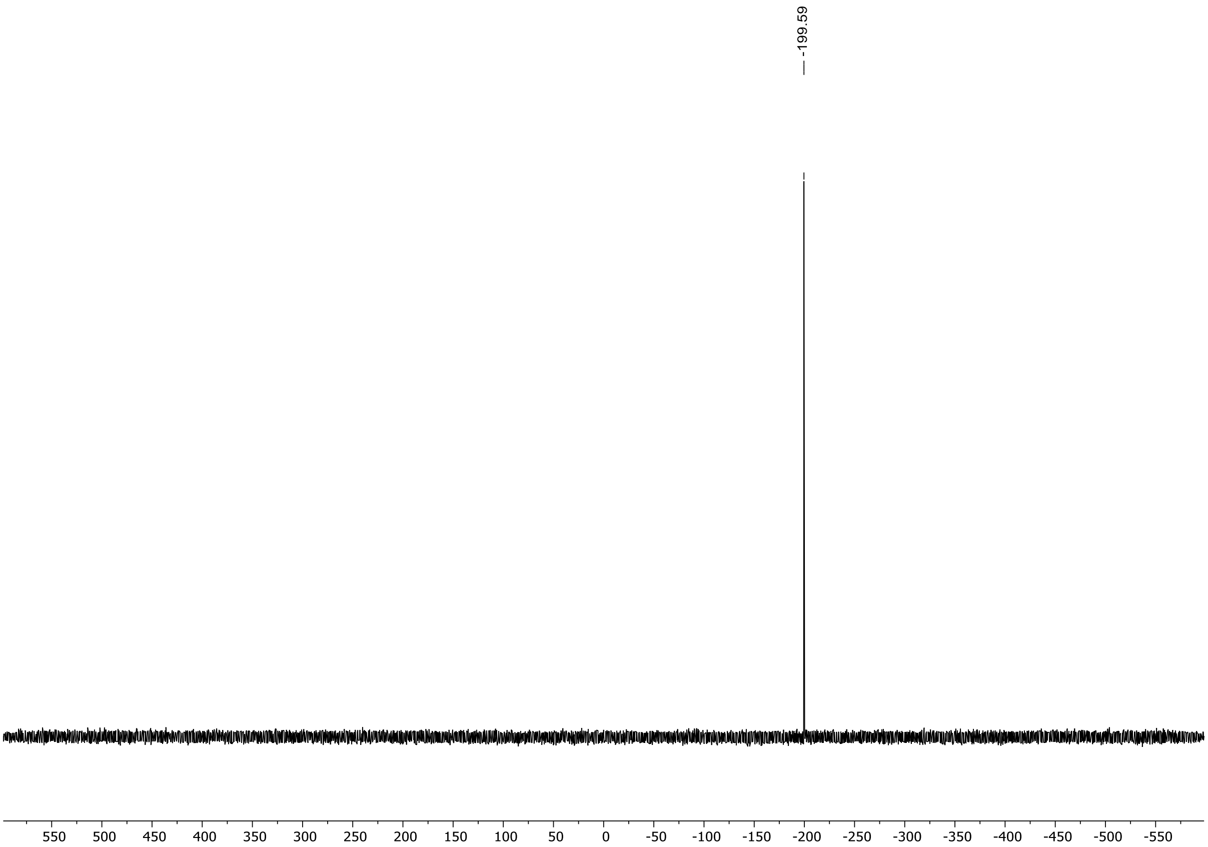

Figure S50. $^{119}Sn\{^{1}H\}$ NMR spectrum of ^{Mes}TerSn(N(SiMe$_3$)$_2$)(CCPh)NHMes (**Sn10a**) (149 MHz, C_6D_6, 298 K).

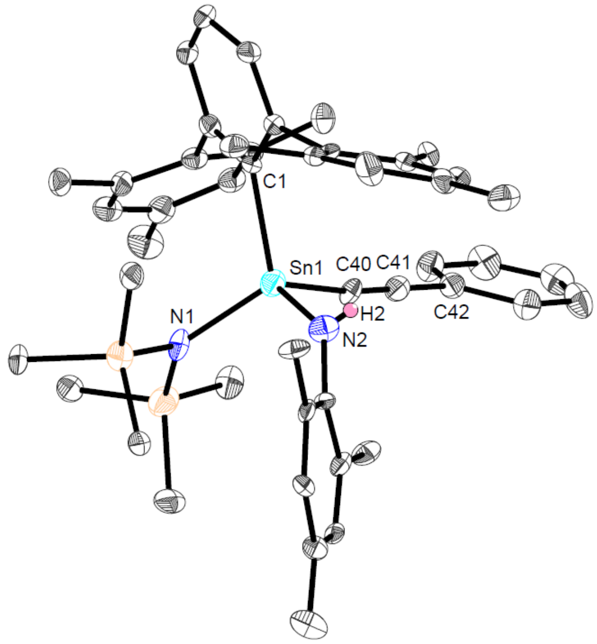

Figure S51. Molecular structure of ^{Mes}TerSn(N(SiMe$_3$)$_2$)(NHMes)CCC$_4$H$_8$Cl (**Sn10a**) in the crystal. Thermal ellipsoids are drawn at the 50% probability level (hydrogen atoms (except H1) have been omitted for clarity). Selected bond lengths (Å) and angles (deg): Sn1–N1 2.048(13), Sn1–N2 2.024(14), Sn1–C1 2.186(15), Sn1–C40 2.099(17), C40–C41 1.18(3), C41–C42 1.45(3), N1–Sn1–N2 106.1(6), N1–Sn1–C40 113.1(6).

Synthesis of ^{Mes}TerSn(N(SiMe$_3$)$_2$)(CC(C$_4$H$_8$)Cl)NHMes (Sn10b)

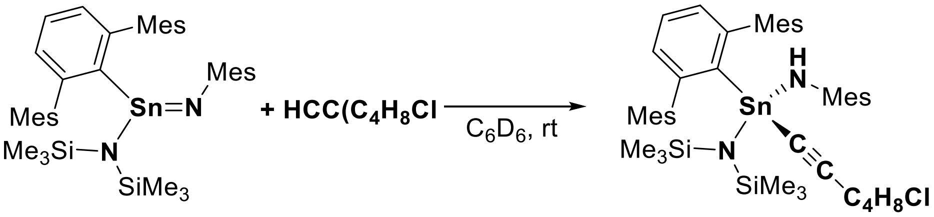

To a solution of ^{Mes}TerSnN(SiMe$_3$)$_2$ (**Sn1**) (0.040 g, 0.068 mmol) in 0.3 mL of C_6D_6 was added a solution of N$_3$Mes (**A2**) (0.011 g, 0.068 mmol) in 0.3 mL of C_6D_6 for the *in situ* generation of ^{Mes}TerSn(N(SiMe$_3$)$_2$)=NMes (**Sn7a**). After the gas evolution has stopped, 6-chlor-hex-1-yne (0.008 g, 0.068 mmol) was added which results in a colour change to a bright yellow-orange. All volatiles were removed under vacuum and 1.0 mL of *n*-hexane were added to the solid followed by filtration and subsequent storage of the saturated solution at -30 °C to give ^{Mes}TerSn(N(SiMe$_3$)$_2$)(CC(C$_4$H$_8$)NHMes (**Sn10b**) as colourless crystals. Crystals obtained this way were suitable for single crystal X-ray diffraction.

Yield: 0.043 g (0.051 mmol; 75%).
^{1}H NMR (400 MHz, C_6D_6, 298 K): δ = 0.15 (s, 18H, Si(CH$_3$)$_3$), 1.35-1.42 (m, 2H, CH$_2$), 1.50-1.56 (m, 2H, CH$_2$), 1.85 (t, $^3J_{H,H}$ = 6.9 Hz, 2H, CH$_2$), 2.14 (s, 6H, CH$_3$), 2.18 (s, 3H, CH$_3$), 2.22 (s, 1H, NH), 2.24 (s, 6H, CH$_3$), 2.31 (s, 6H, CH$_3$), 2.34 (s, 6H, CH$_3$), 3.14 (t, $^3J_{H,H}$ = 6.5 Hz, 2H, CH$_2$), 6.79 (s, 2H, CH$_{Aryl}$), 6.82-6.88 (m, 6H, CH$_{Aryl}$), 7.11-7.14 (m, 1H, CH$_{Aryl}$) ppm.
^{13}C{^{1}H} NMR (101 MHz, C_6D_6, 298 K): δ = 6.5 (Si(CH$_3$)$_3$), 19.9 (CH$_2$), 20.7 (CH$_3$), 21.1 (CH$_3$), 21.8 (CH$_3$), 22.3 (CH$_3$), 22.7 (CH$_3$), 25.4 (CH$_2$), 31.9 (CH$_2$), 44.5 (CH$_2$), 91.0 (Sn satellites: $^1J_{119Sn,C}$ = 831.5 Hz, $^1J_{117Sn,C}$ = 793.5 Hz, Sn$\underline{C}_q$C$_q$), 109.9 (Sn satellites: $^2J_{119Sn,C}$ = 163.8 Hz, $^2J_{117Sn,C}$ = 156.0 Hz, SnC$_q\underline{C}_q$), 129.3 (C$_{q,Aryl}$), 129.37 (CH$_{Aryl}$), 129.42 (CH$_{Aryl}$), 129.7 (CH$_{Aryl}$), 130.1 (CH$_{Aryl}$), 130.9 (CH$_{Aryl}$), 137.2 (C$_{q,Aryl}$), 137.4 (C$_{q,Aryl}$), 137.6 (C$_{q,Aryl}$), 140.2 (C$_{q,Aryl}$), 143.0 (C$_{q,Aryl}$), 144.6 (C$_{q,Aryl}$), 149.1 (C$_{q,Aryl}$) ppm.
^{29}Si{^{1}H} NMR (80 MHz, C_6D_6, 298 K): δ = 5.6 ppm. (assigned by ^{1}H/^{29}Si HMBC)
^{119}Sn{^{1}H} NMR (149 MHz, C_6D_6, 298 K): δ = -203.3 ppm.
EA: Anal. calcd. for $C_{45}H_{63}ClN_2Si_2Sn$: C, 64.17; H, 7.54; N, 3.33; Found: C, 63.88; H, 7.01; N, 2.89.

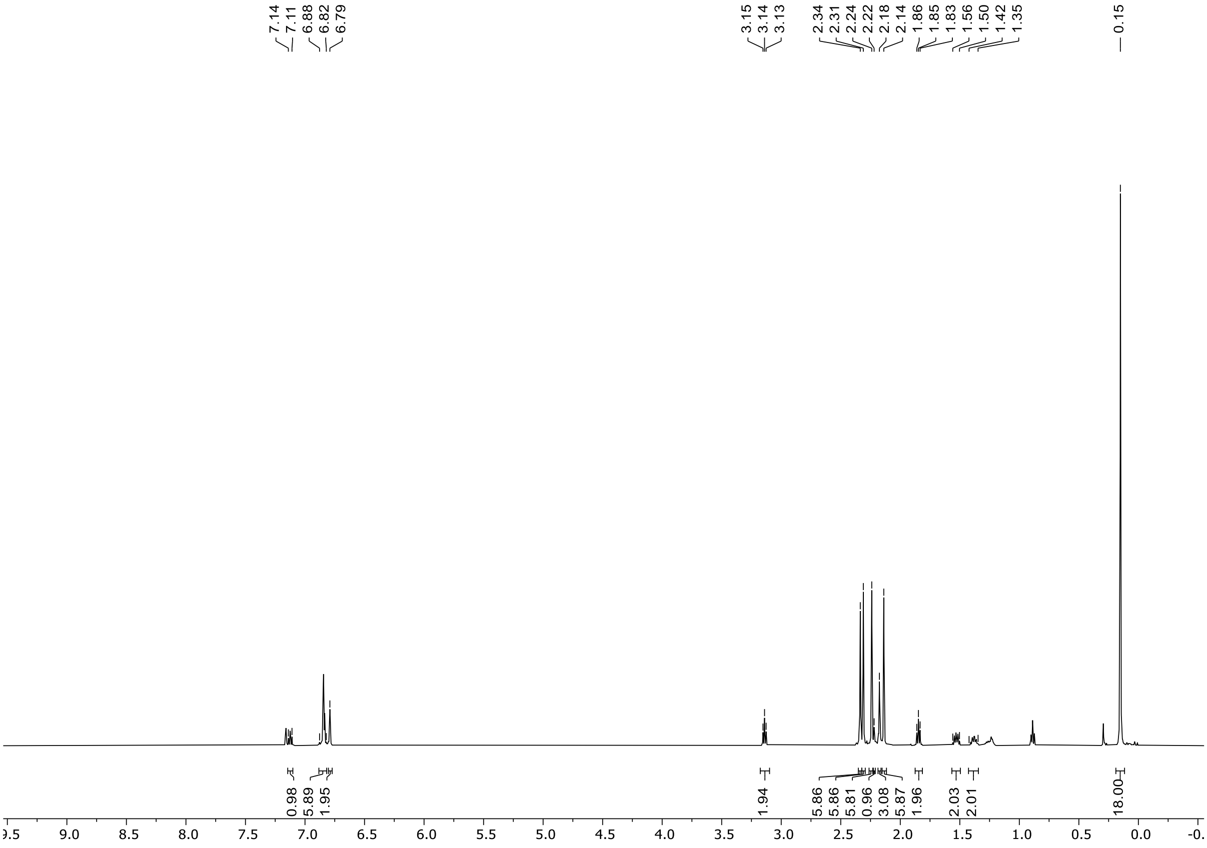

Figure S52. ^{1}H NMR spectrum measured of ^{Mes}TerSn(hmds)(NHMes)CCC$_4$H$_8$Cl (**Sn10b**) (400 MHz, C$_6$D$_6$, 298 K); 0.89, 1.23 ppm: *n*-hexane.

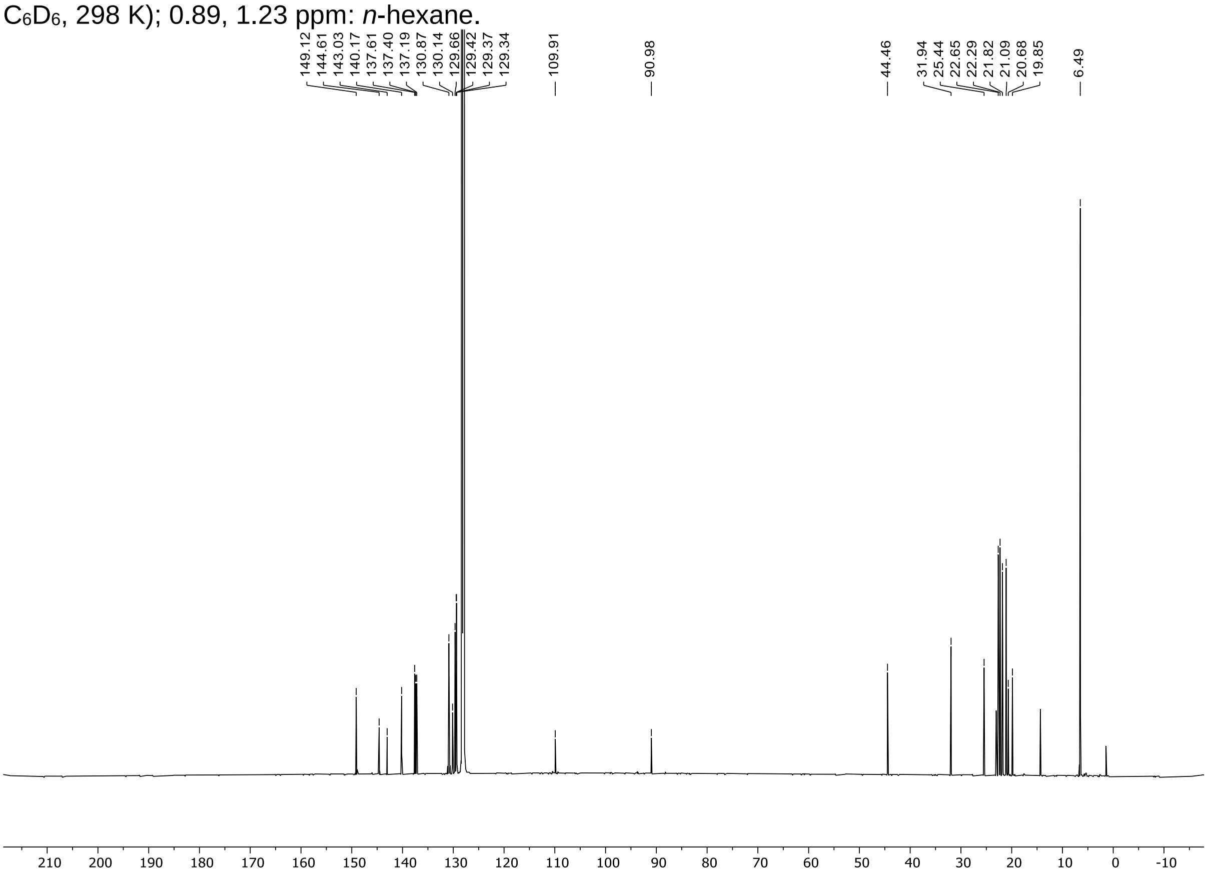

Figure S53. ^{13}C{^{1}H} NMR spectrum of ^{Mes}TerSn(hmds)(NHMes)CCC$_4$H$_8$Cl (**Sn10b**) (400 MHz, C$_6$D$_6$, 298 K); 1.4 ppm: silicon grease; 14.3, 23.0, 32.0 ppm: *n*-hexane.

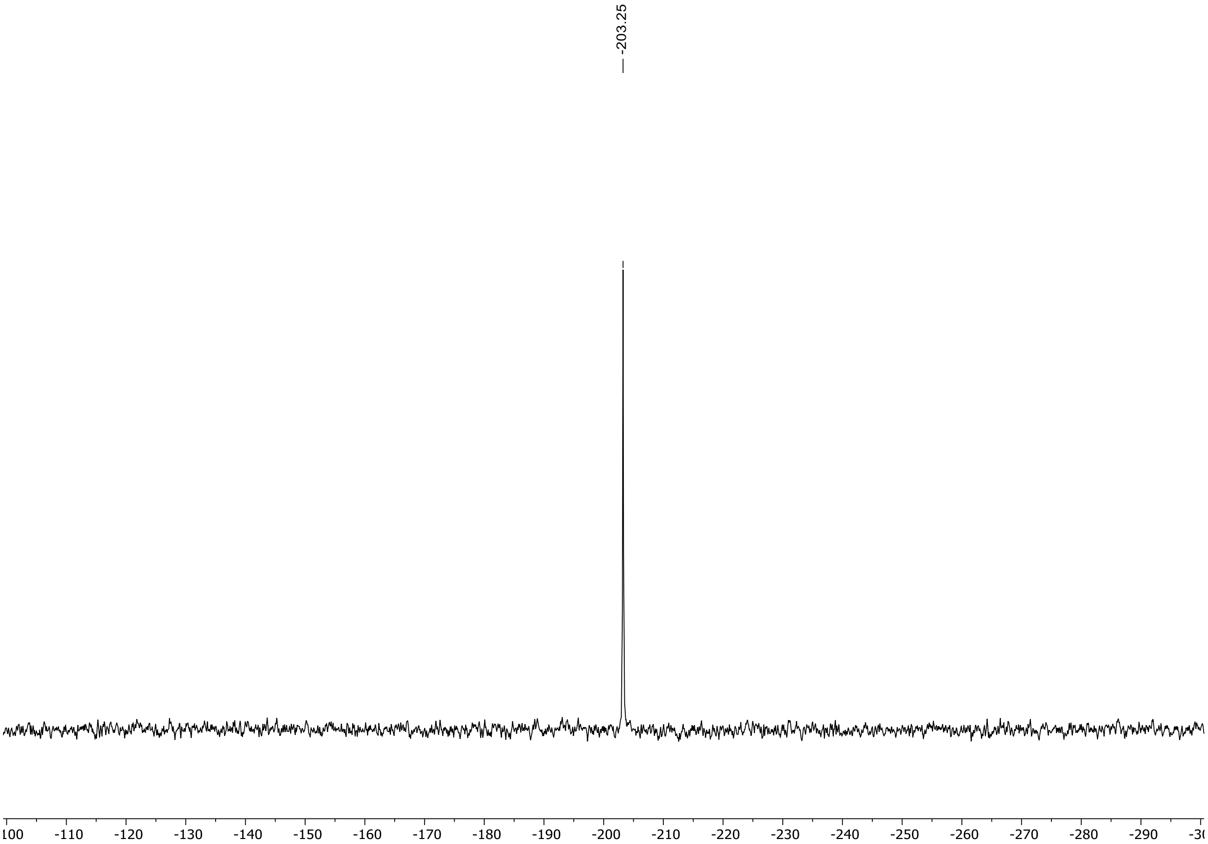

Figure S54. $^{119}Sn\{^{1}H\}$ NMR spectrum of ^{Mes}TerSn(hmds)(NHMes)CCC$_4$H$_8$Cl (**Sn10b**) (149 MHz, C_6D_6, 298 K).

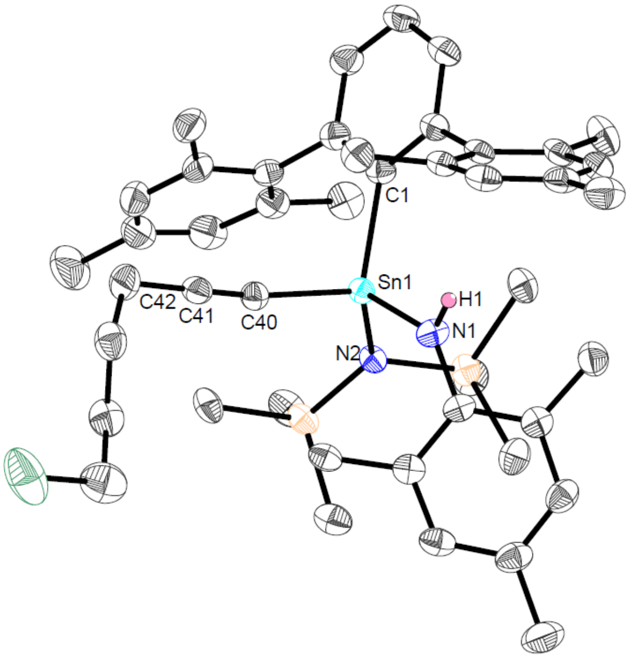

Figure S55. Molecular structure of ^{Mes}TerSn(hmds)(NHMes)CCC$_4$H$_8$Cl (**Sn10b**) in the crystal. Thermal ellipsoids are drawn at the 50% probability level (hydrogen atoms (except H1) have been omitted for clarity). Selected bond lengths (Å) and angles (deg): Sn1–N1 2.057(2), Sn1–N2 2.051(2), Sn1–C1 2.181(3), Sn1–C40 2.087(3), C40–C41 1.189(4), C41–C42 1.468(4), N1–Sn1–N2 106.37(9), N1–Sn1–C40 104.17(10).

Reaction of ^{Mes}TerSn(N(SiMe$_3$)$_2$)=NMes (Sn7a) with CO_2

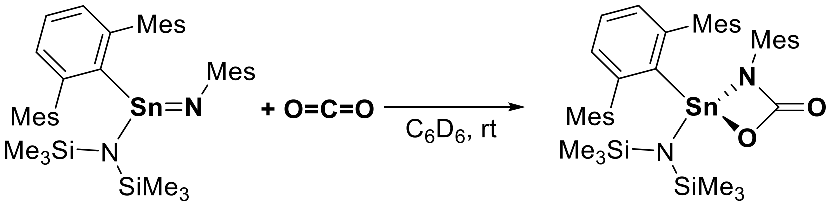

To a solution of ^{Mes}TerSnN(SiMe$_3$)$_2$ (**Sn1**) (0.016 g, 0.027 mmol) in 0.3 mL of C_6D_6 was added a solution of N$_3$Mes (**A2**) (0.004 g, 0.027 mmol) in 0.3 mL of C_6D_6 for the *in situ* generation of ^{Mes}TerSn(N(SiMe$_3$)$_2$)=NMes (**Sn7a**). The solution was freeze-pump-thaw degassed three times and backfilled with 1 bar of CO_2 which results in an immediately occurring colour change from deep red to colourless. After the red colour has disappeared completely, the reaction mixture was analysed by NMR spectroscopy verifying clean formation of the [2+2] cycloaddition product **Sn11a**. The solution was concentrated under vacuum to approximately 0.1 mL followed by addition of 0.5 mL of *n*-heptane which results in precipitation of a colourless solid. The suspension was heated to 100 °C for a prolonged time which results in the formation of colourless crystals above the solution phase. These crystals were suitable for single crystal X-ray diffraction. For isolation of **Sn11a**, ^{Mes}TerSnN(SiMe$_3$)$_2$ (**Sn1**) (0.030 g, 0.051 mmol) in 0.3 mL of C_6D_6 was added to a solution of N$_3$Mes (**A2**) (0.008 g, 0.027 mmol) in 0.3 mL of C_6D_6 for the in situ generation of ^{Mes}TerSn(N(SiMe$_3$)$_2$)=NMes (**Sn7a**). The solution was freeze-pump-thaw degassed three times and backfilled with 1 bar of CO_2. After all starting material has been consumed according to ^{1}H NMR spectroscopy, the solution was transferred to a pre-weight vial. The vial was transferred to a Schlenk tube and the solution was carefully dried under vacuum to give **Sn7a** as a slightly yellow powdery material.

Yield: 0.034 g (0.044 mmol; 86%).
^{1}H NMR (400 MHz, C_6D_6, 298 K): δ = 0.07 (s, 18H, Si(CH$_3$)$_3$), 2.03 (s, 6H, CH$_3$), 2.10 (s, 6H, CH$_3$), 2.18 (s, 3H, CH$_3$), 2.22 (s, 6H, CH$_3$), 2.23 (s, 6H, CH$_3$), 6.41 (s, 2H, CH$_{Aryl}$), 6.72 (s, 2H, CH$_{Aryl}$), 6.76-6.78 (m, 2H, CH$_{Aryl}$), 6.84 (s, 2H, CH$_{Aryl}$), 7.04-7.08 (m, 1H, CH$_{Aryl}$) ppm.
^{13}C{^{1}H} NMR (101 MHz, C_6D_6, 298 K): δ = 5.4 (Si(CH$_3$)$_3$), 20.8 (CH$_3$), 21.2 (CH$_3$), 21.8 (CH$_3$), 22.3 (CH$_3$), 22.6 (CH$_3$), 129.4 (CH$_{Aryl}$), 129.5 (CH$_{Aryl}$), 129.8 (CH$_{Aryl}$), 130.8 (CH$_{Aryl}$), 131.9 (CH$_{Aryl}$), 132.8 (C$_{q,Aryl}$), 133.8 (C$_{q,Aryl}$), 136.4 (C$_{q,Aryl}$), 137.3 (C$_{q,Aryl}$), 138.0 (C$_{q,Aryl}$), 138.9 (C$_{q,Aryl}$), 145.3 (C$_{q,Aryl}$), 148.6 (C$_{q,Aryl}$), 159.3 (OC=O) ppm.
^{29}Si{^{1}H} NMR (80 MHz, C_6D_6, 298 K): δ = 4.3 ppm. (assigned by ^{1}H/^{29}Si HMBC)
^{119}Sn{^{1}H} NMR (149 MHz, C_6D_6, 298 K): δ = -211.4 ppm.

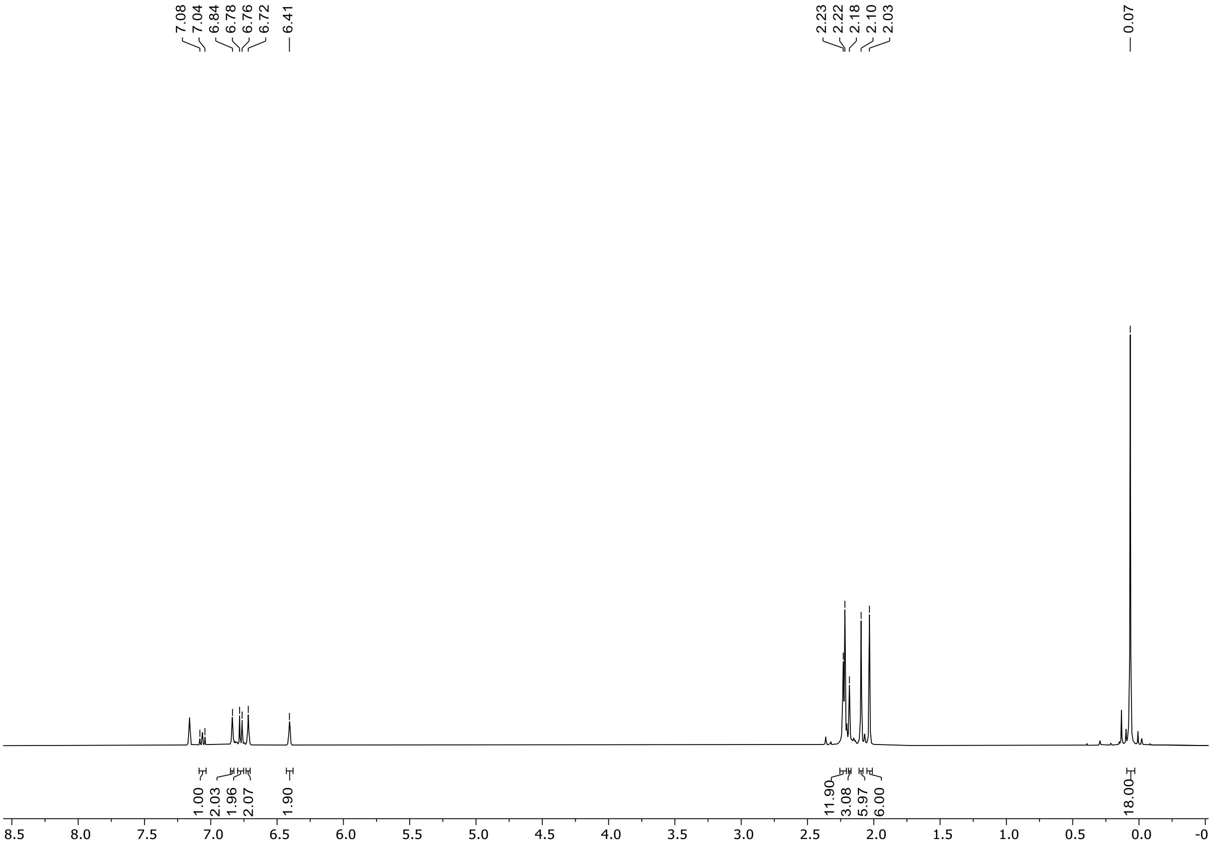

Figure S56. ^{1}H NMR spectrum measured of the [2+2] cycloaddition product **Sn11a** (400 MHz, C_6D_6, 298 K).

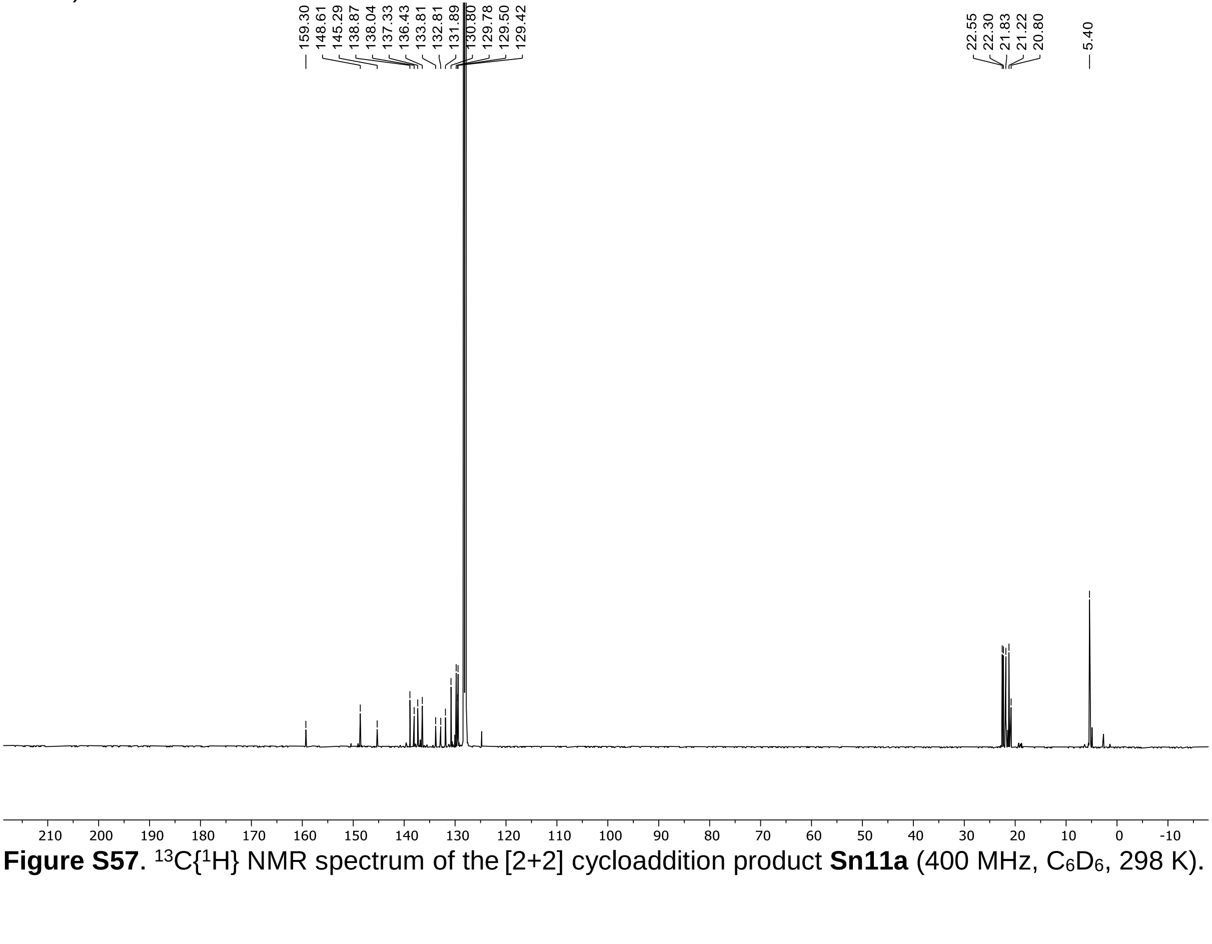

Figure S57. $^{13}C\{^{1}H\}$ NMR spectrum of the [2+2] cycloaddition product **Sn11a** (400 MHz, C_6D_6, 298 K).

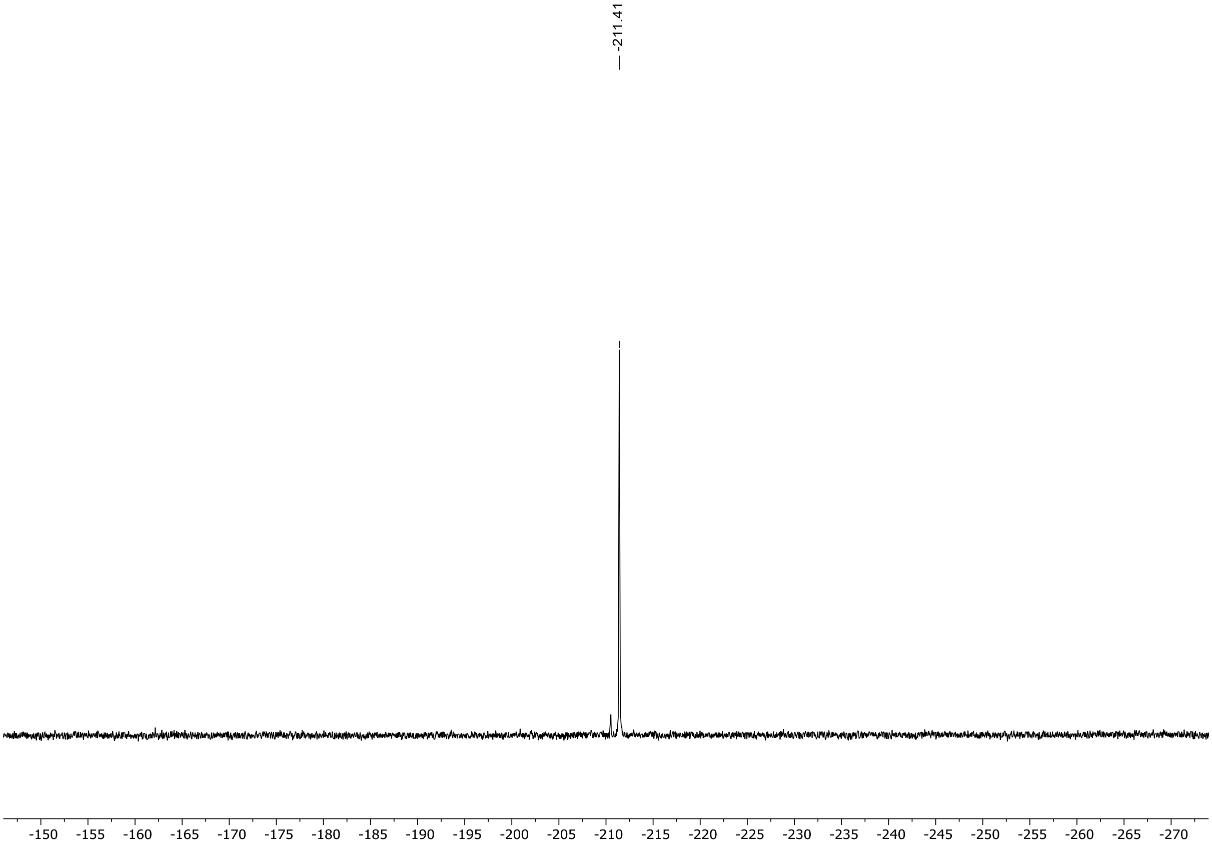

Figure S58. $^{119}Sn\{^1H\}$ NMR spectrum of the [2+2] cycloaddition product **Sn11a** (149 MHz, C_6D_6, 298 K).

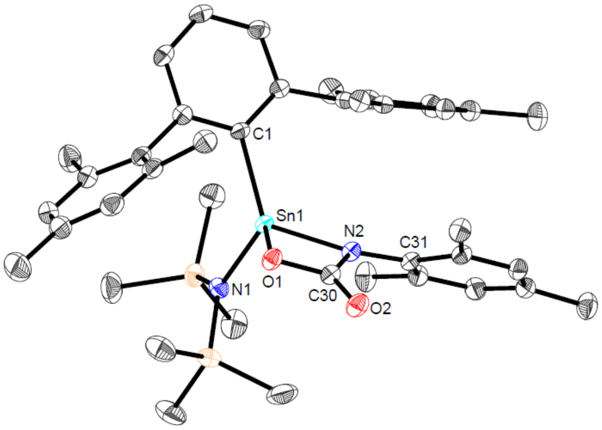

Figure S59. Molecular structure of the [2+2] cycloaddition product **Sn11a** in the crystal. Thermal ellipsoids are drawn at the 50% probability level (hydrogen atoms have been omitted for clarity). Selected bond lengths (Å) and angles (deg): Sn1–N1 2.021(2), Sn1–N2 2.0976(19), Sn1–O1 2.0361(16), Sn1–C1 2.140(2), O1–C30 1.355(3), N2–C30 1.390(3), O2–C30 1.214(3), N1–Sn1–N2 112.46(8), O1–Sn1–N2 65.10(7).

Synthesis of the [2+2] cycloaddition product Sn11b

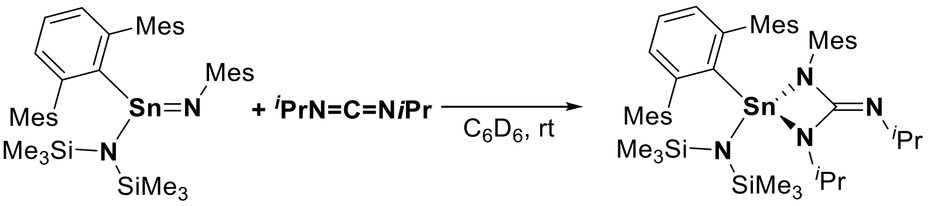

To a solution of ^{Mes}TerSnN(SiMe$_3$)$_2$ (**Sn1**) (0.025 g, 0.042 mmol) in 0.3 mL of C_6D_6 was added a solution of N$_3$Mes (**A2**) (0.007 g, 0.042 mmol) in 0.3 mL of C_6D_6 for the *in situ* generation of ^{Mes}TerSn(N(SiMe$_3$)$_2$)=NMes (**Sn7a**). After the gas evolution has stopped, the solution was added to *N*,*N*'-diisopropylcarbodiimide (0.005 g, 0.042 mmol) which results in a colour change to bright yellow. All volatiles were removed under vacuum and 0.8 mL of *n*-hexane were added to the solid followed by filtration and subsequent storage of the saturated solution at -4 °C to give the [2+2] cycloaddition product **Sn11b** as colourless crystals. Crystals obtained this way were suitable for single crystal X-ray diffraction.

Upscale experiment: To a solution of ^{Mes}TerSnN(SiMe$_3$)$_2$ (**Sn1**) (0.500 g, 0.844 mmol) in 4 mL of benzene N$_3$Mes (**A2**) (0.136 g, 0.844 mmol) in 1 mL of benzene was added and the reaction mixture was stirred until the gas evolution has stopped. An aliquot was taken (0.2 mL), dried and analysed by ^{1}H NMR spectroscopy to prove the purity of ^{Mes}TerSn(N(SiMe$_3$)$_2$)=NMes (**Sn7a**) (Figure S62). To the remaining solution *N*,*N*'-diisopropylcarbodiimide (0.106 g, 0.844 mmol) in 1 mL of benzene was added and the reaction mixture was stirred for 16 h at room temperature to give a slightly yellow solution. All volatile components have been removed under vacuum to give **Sn11b** as a slightly yellow powdery material (corresponding ^{1}H NMR shown in Figure S63).

Yield: 0.018 g (0.021 mmol; 50%); Upscale: 0.628 g (0.737 mmol; 87%).
^{1}H NMR (400 MHz, C_6D_6, 298 K): δ = 0.06 (s, 18H, Si(CH$_3$)$_3$), 1.03 (d, $^3J_{H,H}$ = 6.1 Hz, 3H, CH(C*H*$_3$)$_2$), 1.21 (d, $^3J_{H,H}$ = 6.1 Hz, 3H, CH(C*H*$_3$)$_2$), 1.49 (d, $^3J_{H,H}$ = 6.5 Hz, 3H, CH(C*H*$_3$)$_2$), 1.61 (d, $^3J_{H,H}$ = 6.5 Hz, 3H, CH(C*H*$_3$)$_2$), 1.81 (s, 3H, CH$_3$), 2.16 (s, 3H, CH$_3$), 2.18-2.19 (m, 12H, CH$_3$), 2.22 (s, 6H, CH$_3$), 2.46 (s, 3H, CH$_3$), 3.20 (hept, $^3J_{H,H}$ = 6.0 Hz, 1H, CH(C*H*$_3$)$_2$), 3.55 (hept, $^3J_{H,H}$ = 6.5 Hz, 1H, CH(C*H*$_3$)$_2$), 6.90-6.70 (m, 2H, CH$_{Aryl}$), 6.72-6.74 (m, 2H, CH$_{Aryl}$), 6.77 (s, 2H, CH$_{Aryl}$), 6.83 (s, 2H, CH$_{Aryl}$), 7.01-7.04 (m, 1H, CH$_{Aryl}$) ppm.
^{13}C{^{1}H} NMR (101 MHz, C_6D_6, 298 K): δ = 6.0 (Si(CH$_3$)$_3$), 19.6 (CH$_3$), 20.9 (CH$_3$), 21.1 (CH$_3$), 22.4 (CH$_3$), 22.5 (CH($\underline{C}$H$_3$)$_2$), 23.0 (CH($\underline{C}$H$_3$)$_2$), 23.0 (CH$_3$), 23.2 (CH$_3$), 25.6 (CH($\underline{C}$H$_3$)$_2$), 26.1 (CH($\underline{C}$H$_3$)$_2$), 46.6 ($\underline{C}$H(CH$_3$)$_2$), 48.2 ($\underline{C}$H(CH$_3$)$_2$), 129.4 (CH$_{Aryl}$), 129.6 (CH$_{Aryl}$), 129.9 (C$_{q,Aryl}$), 130.0 (CH$_{Aryl}$), 130.7 (CH$_{Aryl}$), 131.8 (CH$_{Aryl}$), 132.9 (C$_{q,Aryl}$), 135.2 (C$_{q,Aryl}$), 136.5 (C$_{q,Aryl}$), 137.5 (C$_{q,Aryl}$), 137.8 (C$_{q,Aryl}$), 138.4 (C$_{q,Aryl}$), 139.9 (C$_{q,Aryl}$), 142.1 (C$_{q,Aryl}$), 147.0 (C$_{q,Aryl}$), 148.7 (C$_{q,Aryl}$) 154.9 (NC=N) ppm.
^{29}Si{^{1}H} NMR (80 MHz, C_6D_6, 298 K): δ = 5.8 ppm. (assigned by ^{1}H/^{29}Si HMBC)
^{119}Sn{^{1}H} NMR (149 MHz, C_6D_6, 298 K): δ = -151.3 ppm.
EA: Anal. calcd. for $C_{46}H_{68}N_4Si_2Sn$: C, 64.85; H, 8.05; N, 6.58; Found: C, 64.06; H, 7.72; N, 5.91 (material from the upscale experiment).

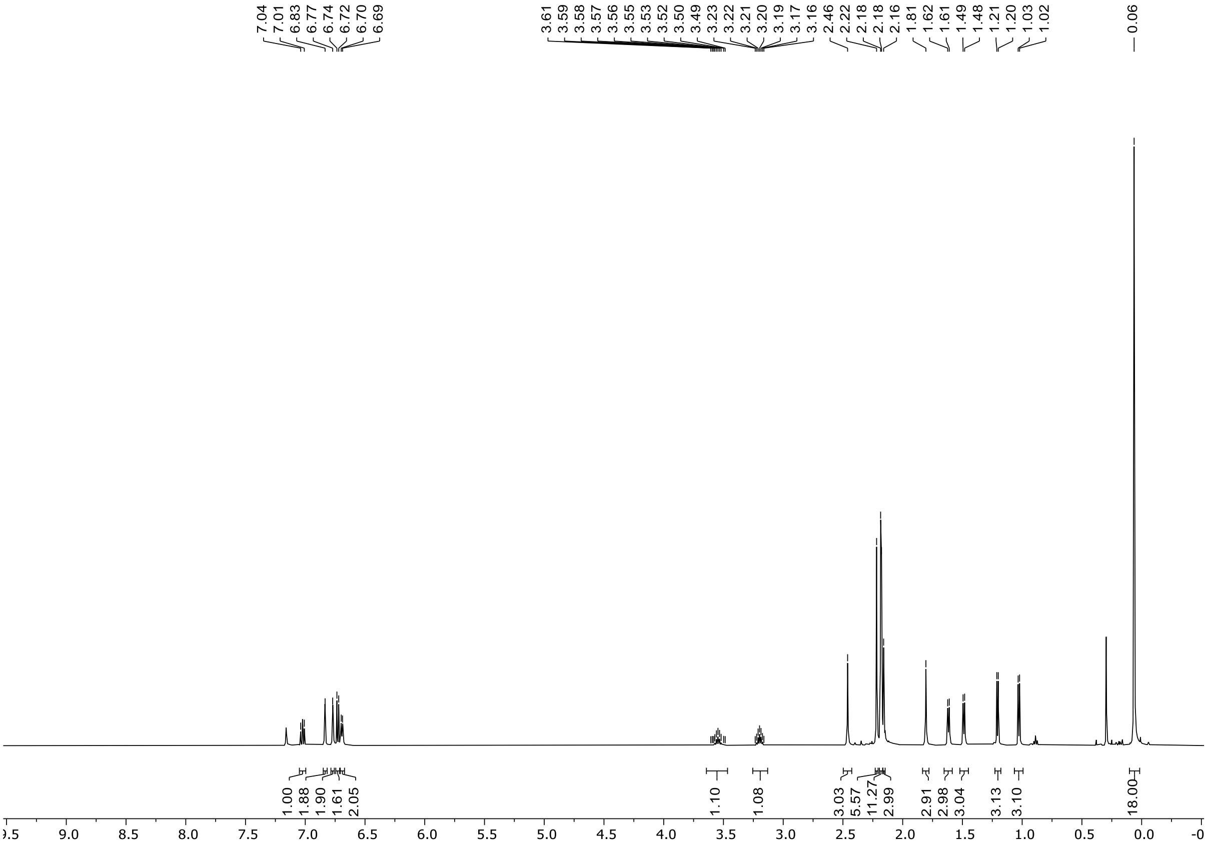

Figure S60. ^{1}H NMR spectrum of the [2+2] cycloaddition product **Sn11b** (400 MHz, C_6D_6, 298 K); 0.29 ppm: silicon grease; 0.89, 1.23 ppm: *n*-hexane.

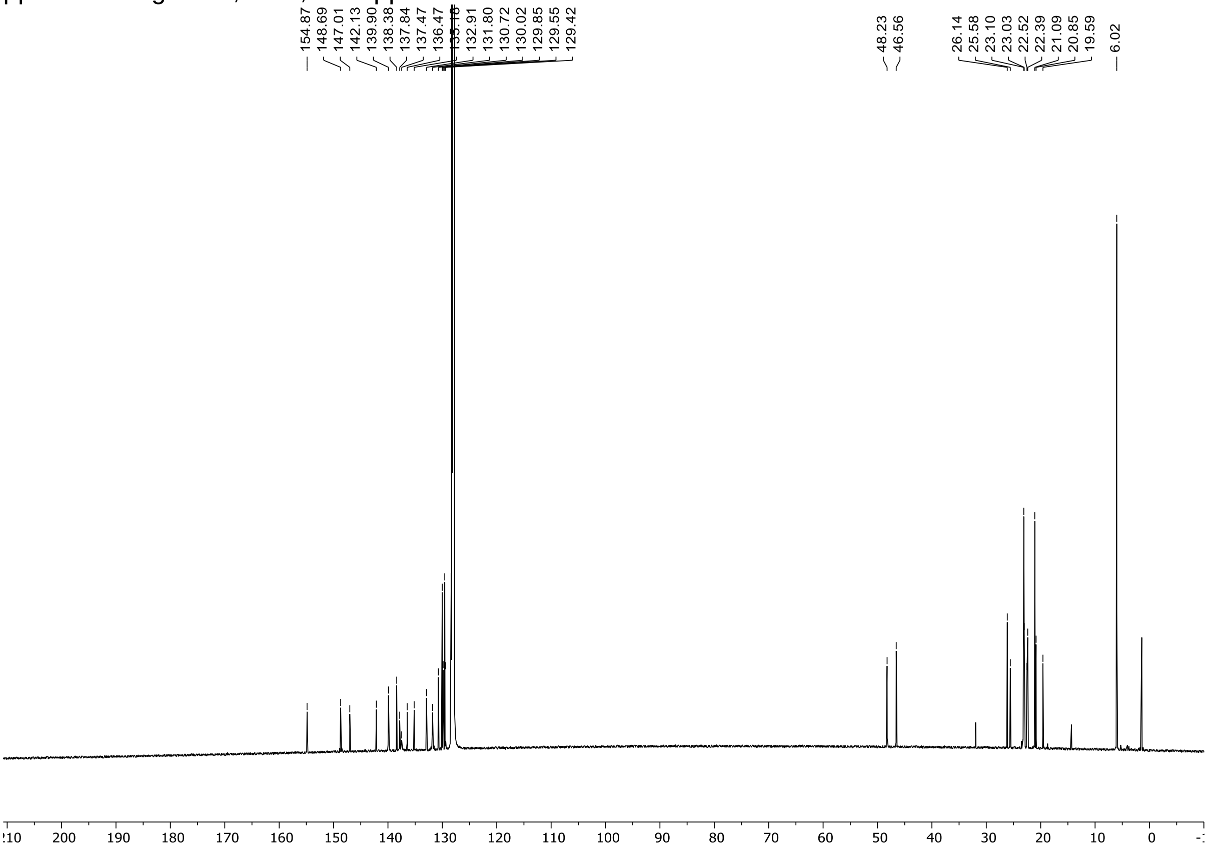

Figure S61. $^{13}C\{^{1}H\}$ NMR spectrum of the [2+2] cycloaddition product **Sn11b** (400 MHz, C_6D_6, 298 K); 1.4 ppm: silicon grease; 14.3, 23.0, 32.0 ppm: *n*-hexane.

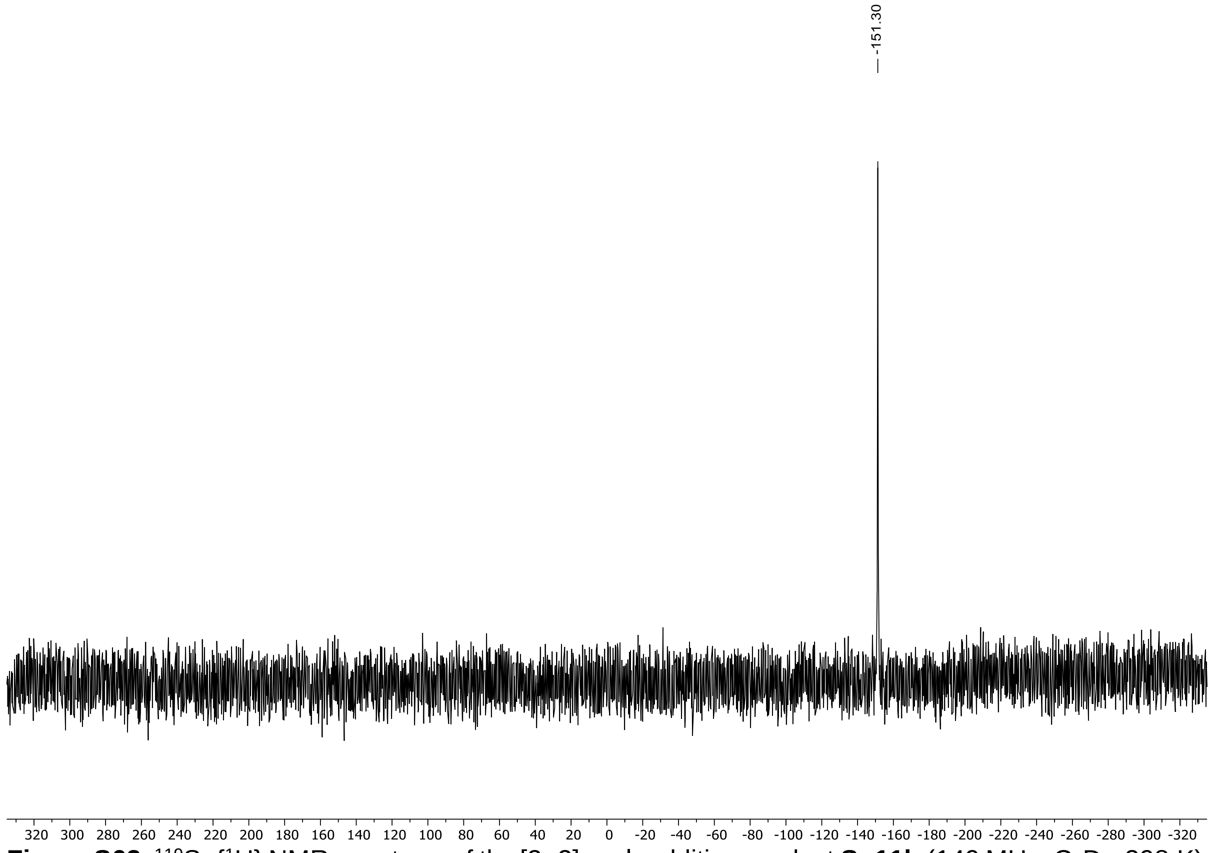

Figure S62. $^{119}Sn\{^{1}H\}$ NMR spectrum of the [2+2] cycloaddition product **Sn11b** (149 MHz, C_6D_6, 298 K).

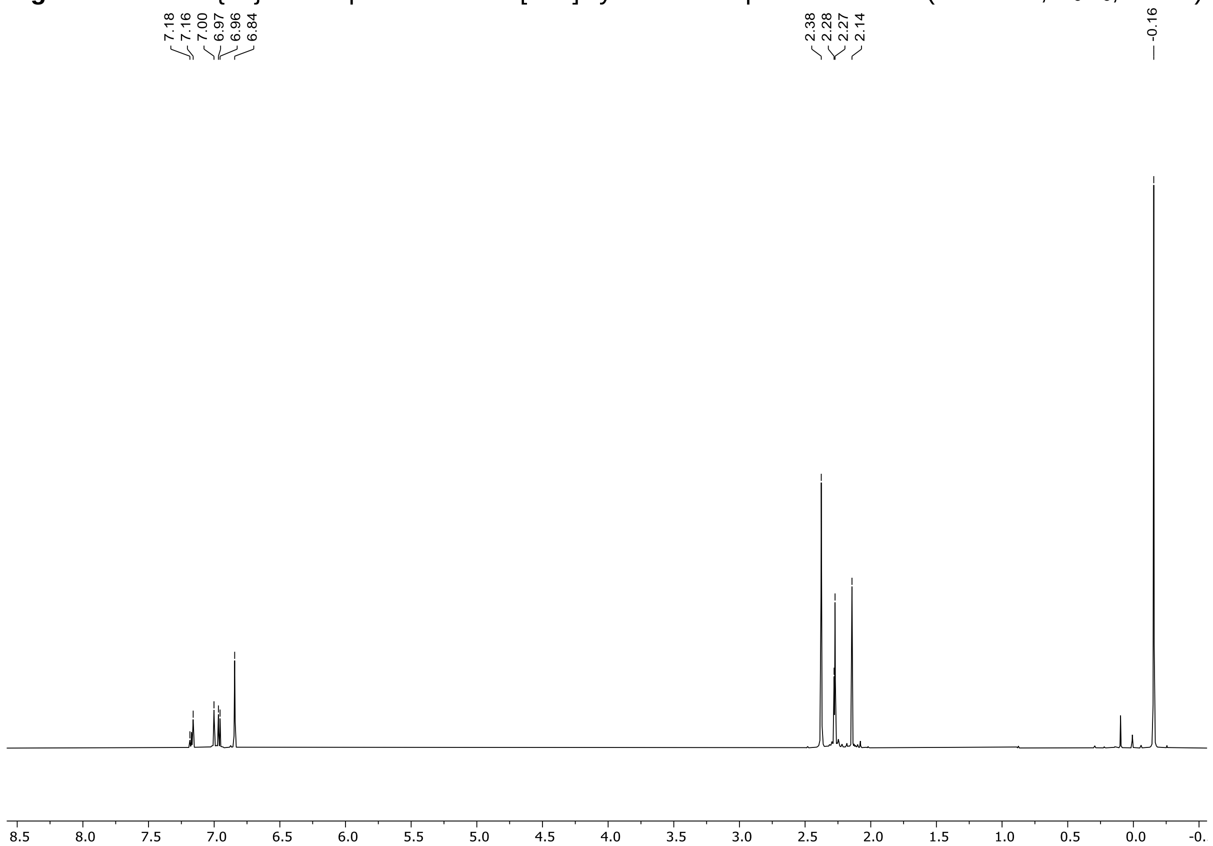

Figure S63. ^{1}H NMR spectrum of $^{Mes}TerSn(N(SiMe_3)_2)=NMes$ (**Sn7a**) obtained from the upscale experiment (400 MHz, C_6D_6, 298 K)**.**

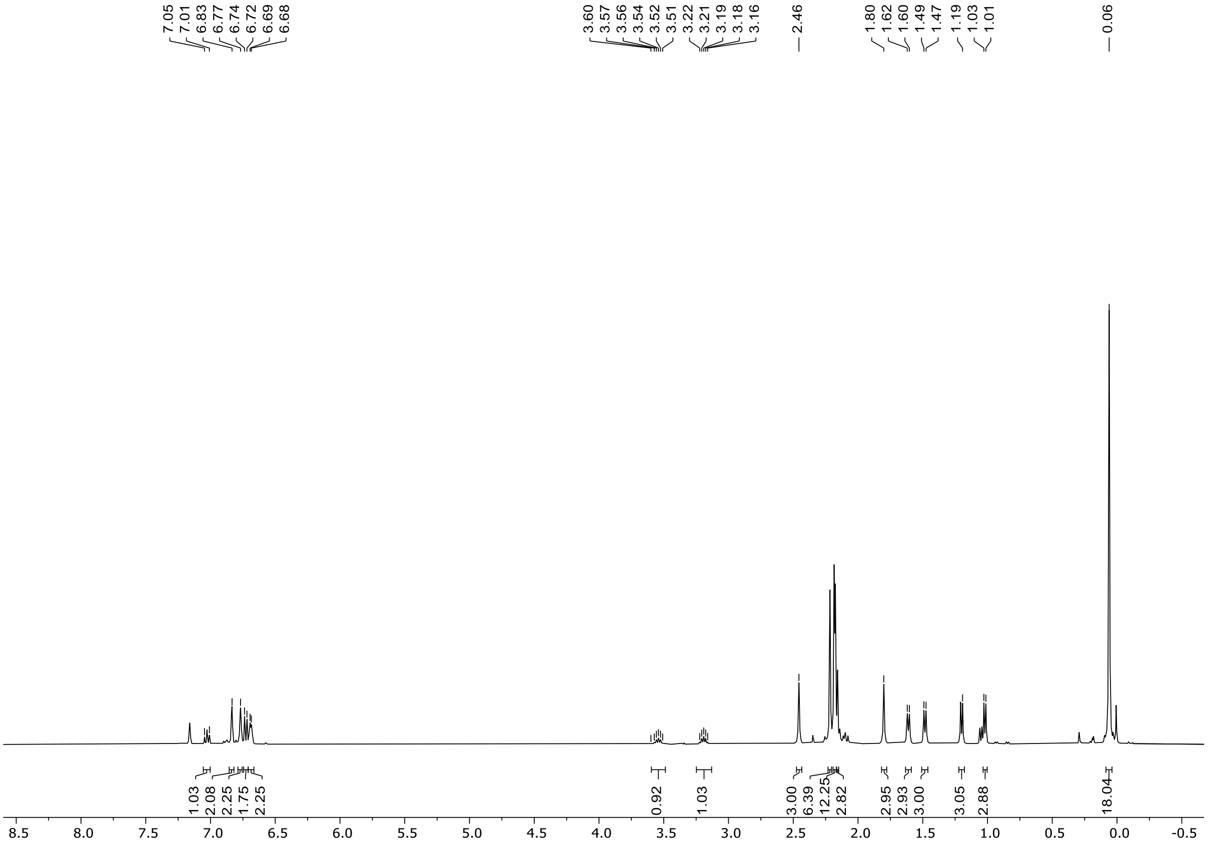

Figure S64. ^{1}H NMR spectrum of the [2+2] cycloaddition product **Sn11b** obtained from the upscale experiment (400 MHz, C_6D_6, 298 K)**.**

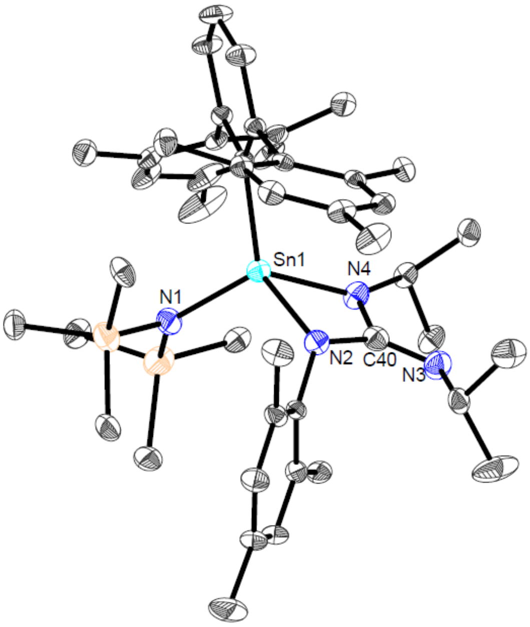

Figure S65. Molecular structure of the [2+2] cycloaddition product **Sn11b** in the crystal. Thermal ellipsoids are drawn at the 50% probability level (hydrogen atoms (except H1) have been omitted for clarity). Selected bond lengths (Å) and angles (deg): Sn1–N1 2.052(2), Sn1–N2 2.100(2), Sn1–N4 2.058(2), Sn1–C1 2.189(3), N2–C40 1.421(4), N4–C40 1.391(4), N3–C40 1.280(4), N1–Sn1–N2 107.53(9), N2–Sn1–N4 65.67(9).

Reaction of ^{Mes}TerSn(N(SiMe$_3$)$_2$)=NMes (Sn7a) with CS$_2$

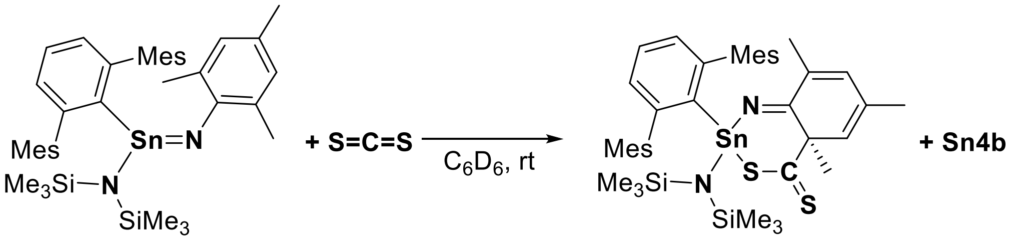

To a solution of ^{Mes}TerSnN(SiMe$_3$)$_2$ (**Sn1**) (0.035 g, 0.059 mmol) in 0.3 mL of C_6D_6 was added a solution of N$_3$Mes (**A2**) (0.010 g, 0.059 mmol) in 0.3 mL of C_6D_6 for the *in situ* generation of ^{Mes}TerSn(N(SiMe$_3$)$_2$)=NMes (**Sn7a**). An excess of CS$_2$ (5 drops with a 1 mL syringe) was added and the reaction progress was monitored by ^{1}H NMR spectroscopy revealing complete consumption of **Sn7a** within 16 h at room temperature. The corresponding ^{1}H NMR spectrum (Figure S61) revealed that significant amounts of the intramolecular C–H activation product **Sn4b** have been formed alongside a single other product (ratio approximately 1.5:1.0 as estimated by integration of appropriate ^{1}H NMR signals). All volatile components were removed under vacuum, the remaining solid was suspended in 0.7 mL of *n*-hexane, filtered and stored at -30 °C. Both **Sn4b** and the [4+2] cycloaddition product **Sn12** co-crystallized. Red crystals of **Sn12** were suitable for single crystal X-ray diffraction. Crystals of **Sn12** were separated as good as possible and allowed for assignment of the main signals by ^{1}H NMR spectroscopy (Figure S62). Due to these difficulties only the ^{1}H NMR data and the results of single crystal X-ray diffraction are given.

^{1}H NMR (400 MHz, C_6D_6, 298 K): δ = 0.11 (s(br), 9H, Si(CH$_3$)$_3$), 0.17 (s(br), 9H, Si(CH$_3$)$_3$), 1.59 (s, 3H, CH$_3$), 1.68 (s, 3H, CH$_3$), 1.87 (s, 3H, CH$_3$), 2.15 (s, 6H, CH$_3$), 2.20 (s, 6H, CH$_3$), 2.30 (s, 6H, CH$_3$), 6.08 (s, 1H, CH$_{Aryl}$), 6.28 (s, 1H, CH$_{Aryl}$), 6.75-6.81 (m, 6H, CH$_{Aryl}$), 7.07-7.11 (m, 1H, CH$_{Aryl}$) ppm.

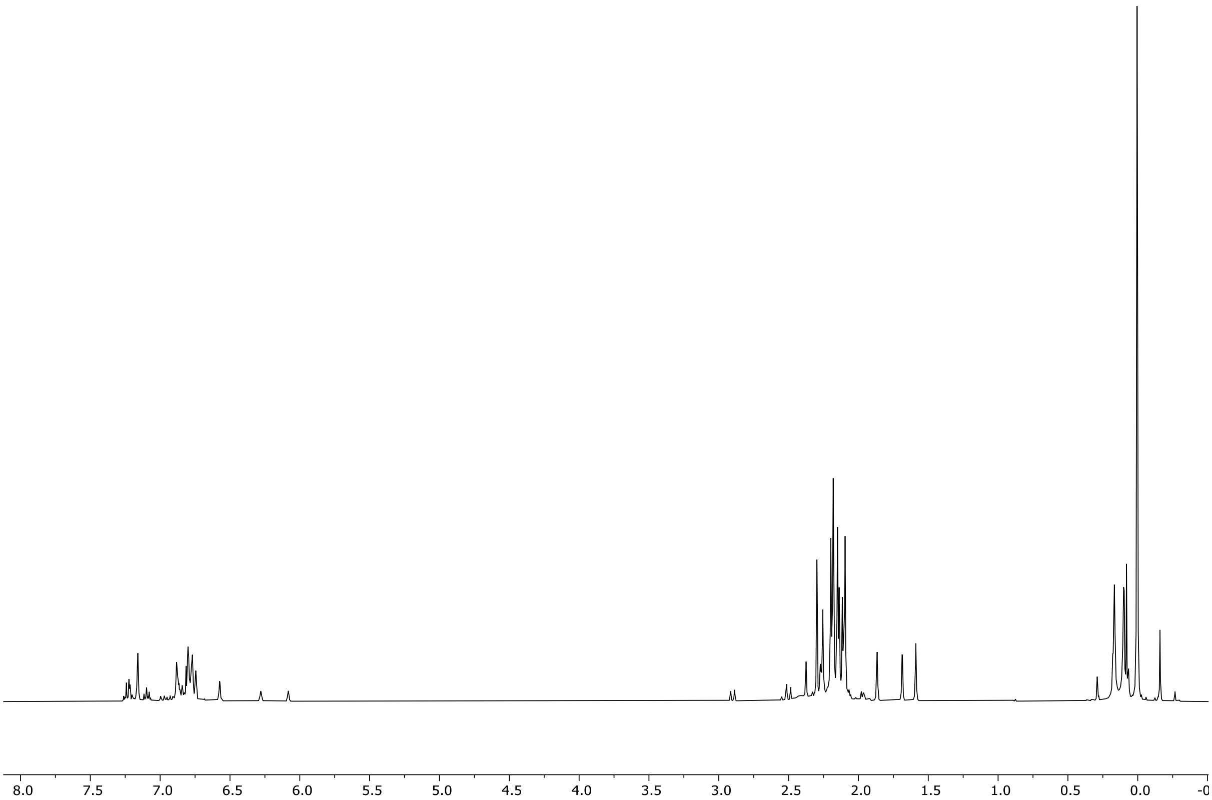

Figure S66. ^{1}H NMR spectrum after 16 h at room temperature of the reaction of ^{Mes}TerSn(N(SiMe$_3$)$_2$)=NMes (**Sn7a**) with CS_2 (400 MHz, C_6D_6, 298 K).

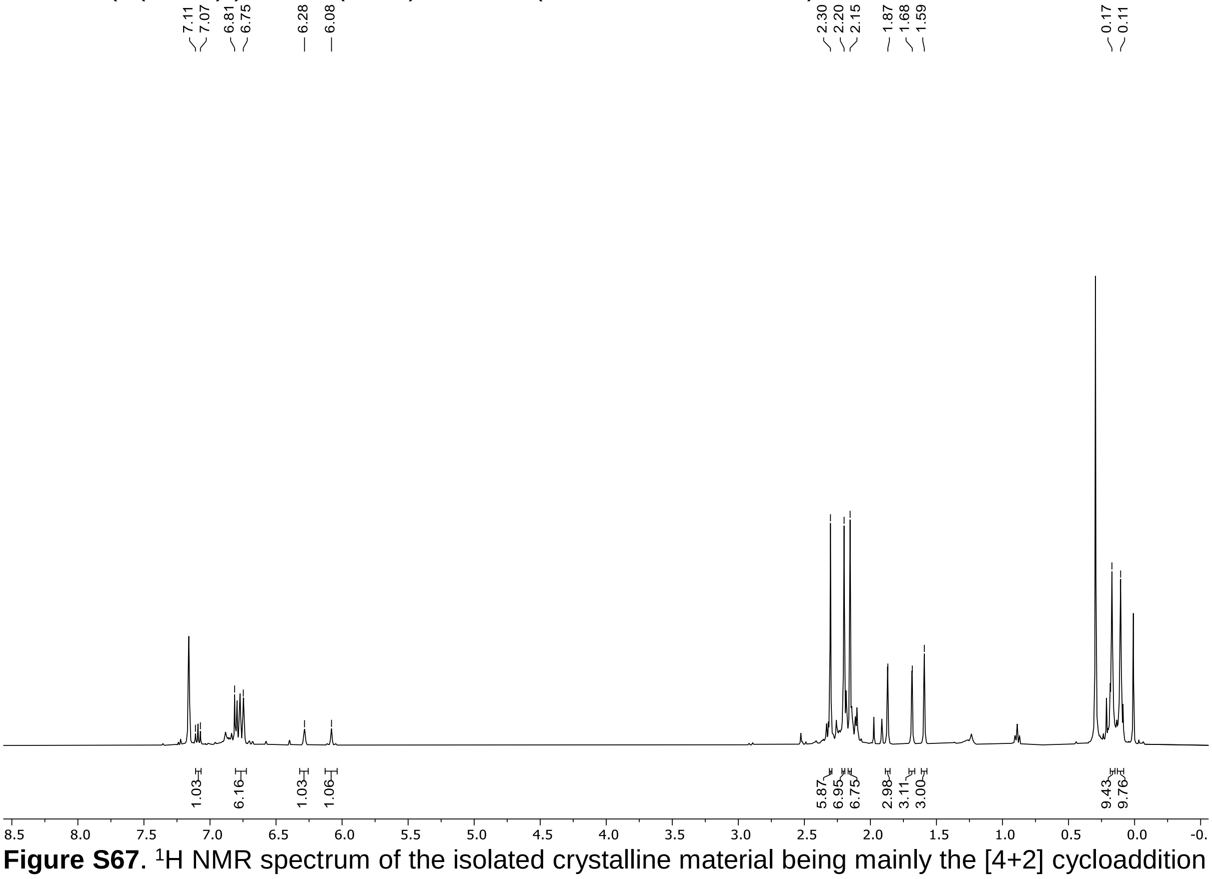

Figure S67. ^{1}H NMR spectrum of the isolated crystalline material being mainly the [4+2] cycloaddition product **Sn12** (400 MHz, C_6D_6, 298 K); 0.29 ppm: silicon grease.

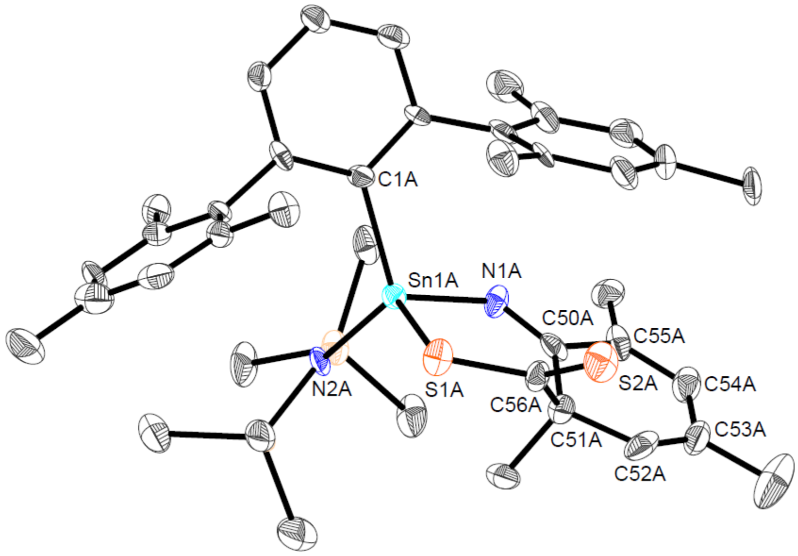

Figure S68. Molecular structure of the [4+2] cycloaddition product **Sn12** in the crystal. Thermal ellipsoids are drawn at the 50% probability level (hydrogen atoms have been omitted for clarity). Selected bond lengths (Å) and angles (deg): Sn1A–N1A 2.036(11), Sn1A–N2A 2.058(9), Sn1A–C1A 2.152(11), Sn1A–S1A 2.438(3), S1A–C56A 1.775(13), S2A–C56A 1.649(14), N1A–C50A 1.269(17), C50A–C51A 1.559(19), C51A–C52A 1.502(18), C52A–C53A 1.31(2), C53A–C54A 1.48(2), C54A–C55A 1.37(2), C50A–C55A 1.483(18), C51A–C56A 1.503(19), N1A–Sn1A–S1A 98.9(3), N1A–Sn1A–C1A 110.7(4).

Reaction of ^{Mes}TerSn(N(SiMe$_3$)$_2$)=NMes (Sn7a) with SCNXyl

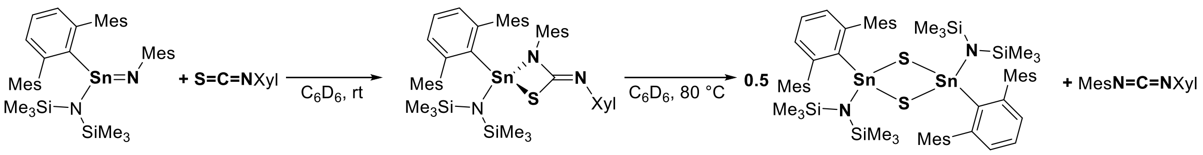

To a solution of ^{Mes}TerSnN(SiMe$_3$)$_2$ (**Sn1**) (0.030 g, 0.051 mmol) in 0.3 mL of C_6D_6 was added a solution of N$_3$Mes (**A2**) (0.008 g, 0.051 mmol) in 0.3 mL of C_6D_6 for the *in situ* generation of ^{Mes}TerSn(N(SiMe$_3$)$_2$)=NMes (**Sn7a**). SCNXyl (0.008 g, 0.051 mmol) in 0.1 mL of C_6D_6 was added and the reaction progress was monitored by ^{1}H NMR spectroscopy until ^{Mes}TerSn(N(SiMe$_3$)$_2$)=NMes (**Sn7a**) was consumed. All volatile components were removed under vacuum, the remaining solid was washed with *n*-hexane (2 x 0.5 mL), dried under vacuum and the colourless solid was dissolved in 0.6 mL of C_6D_6 for NMR characterization indicating the formation of the [2+2] cycloaddition product **Sn11c**. Colourless crystals of **Sn11c** suitable for single crystal X-ray diffraction were obtained from the combined wash solutions after storage at room temperature for several days. For isolation of **Sn11c**, ^{Mes}TerSnN(SiMe$_3$)$_2$ (**Sn1**) (0.030 g, 0.051 mmol) in 0.3 mL of C_6D_6 was added to a solution of N$_3$Mes (**A2**) (0.008 g, 0.027 mmol) in 0.3 mL of C_6D_6 for the in situ generation of ^{Mes}TerSn(N(SiMe$_3$)$_2$)=NMes (**Sn7a**). SCNXyl (0.008 g, 0.051 mmol) in 0.1 mL of C_6D_6 was added and the solution was transferred to a pre-weight vial. The vial was transferred to a Schlenk tube and the solution was carefully dried under vacuum to give **Sn11c** as a colourless solid. Heating a solution of the [2+2] cycloaddition product **Sn11c** to 80 °C for one hour results in clean formation of (^{Mes}TerSn(hmds)S)$_2$ (**Sn13**) and MesN=C=NXyl (Figure S68). This reaction also occurs slowly at room temperature. Therefore, only a ^{13}C{^{1}H} NMR spectrum of a mixture of **Sn11c**, **Sn13** and MesN=C=NXyl is given below and only the clearly assignable signals are listed.

NMR data of the [2+2] cycloaddition product Sn11c:
Yield: 0.043 g (0.048 mmol; 94%).
^{1}H NMR (400 MHz, C_6D_6, 298 K): δ = 0.06 (s, 18H, Si(CH$_3$)$_3$), 1.97 (s, 12H, CH$_3$), 2.15 (s, 3H, CH$_3$, 2.27 (s, 3H, CH$_3$), 2.37-2.42 (m(br), 12H, CH$_3$), 2.55 (s, 3H, CH$_3$), 6.58 (s, 2H, CH$_{Aryl}$), 6.70 (s, 2H, CH$_{Aryl}$), 6.76-6.78 (m, 4H, CH$_{Aryl}$), 6.88-6.92 (m, 1H, CH$_{Aryl}$), 7.04-7.08 (m, 3H, CH$_{Aryl}$) ppm.
^{13}C{^{1}H} NMR (101 MHz, C_6D_6, 298 K): δ = 5.8 (Si(CH$_3$)$_3$), 19.6 (br, CH$_3$), 20.9 (CH$_3$), 21.32 (CH$_3$), 21.34 (CH$_3$), 21.8 (br, CH$_3$), 23.5 (br, CH$_3$), 122.4 (CH$_{Aryl}$), 131.5 (CH$_{Aryl}$), 132.1 (CH$_{Aryl}$), 141.4 (C$_{q,Aryl}$), 144.3 (C$_{q,Aryl}$), 149.4 (C$_{q,Aryl}$), 156.5 (SC=N) ppm.
^{29}Si{^{1}H} NMR (80 MHz, C_6D_6, 298 K): δ = 7.0 ppm. (assigned by ^{1}H/^{29}Si HMBC)
^{119}Sn{^{1}H} NMR (149 MHz, C_6D_6, 298 K): δ = -155.7 ppm.
EA: Anal. calcd. for $C_{48}H_{63}N_3SSi_2Sn$: C, 64.85; H, 7.14; N, 4.73; Found: C, 65.01; H, 7.21; N, 4.01.

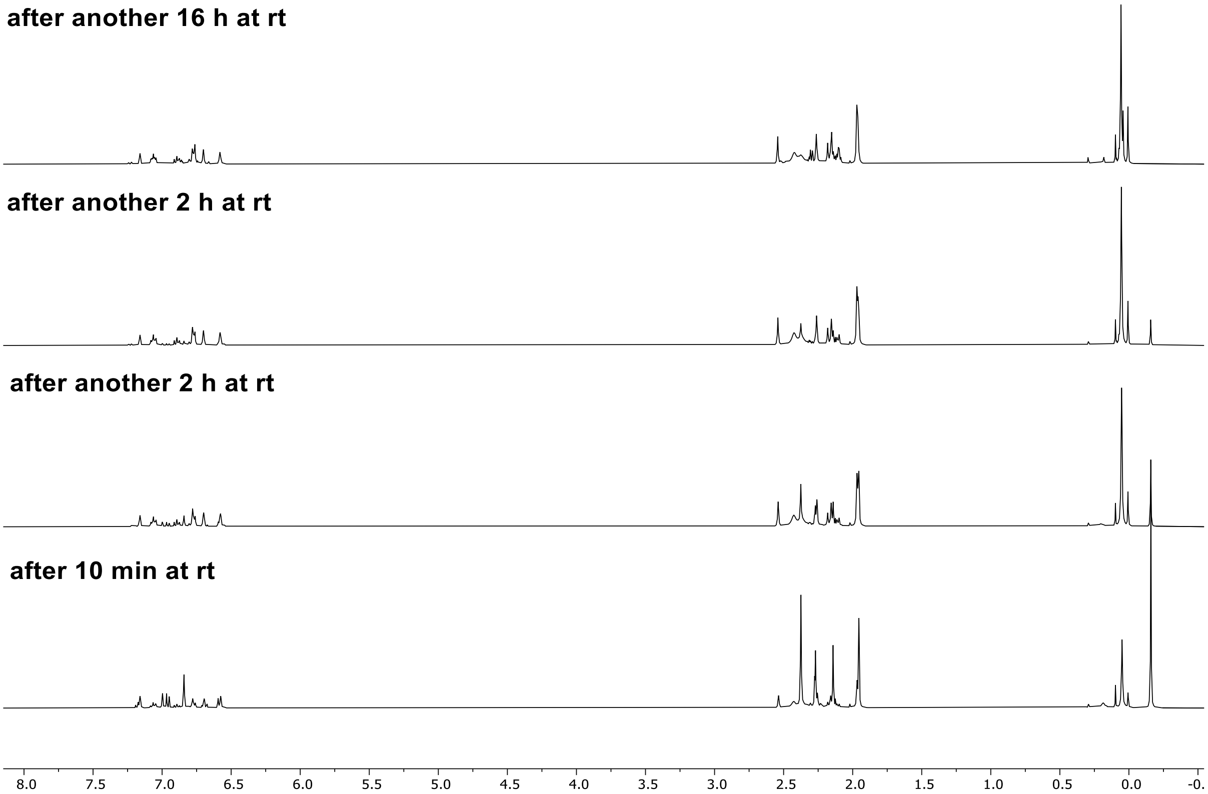

Figure S69. Monitoring of the reaction of $^{Mes}TerSnN(SiMe_3)_2$ (**Sn1**) with SCNXyl via ^1H NMR spectroscopy (400 MHz, C_6D_6, 298 K).

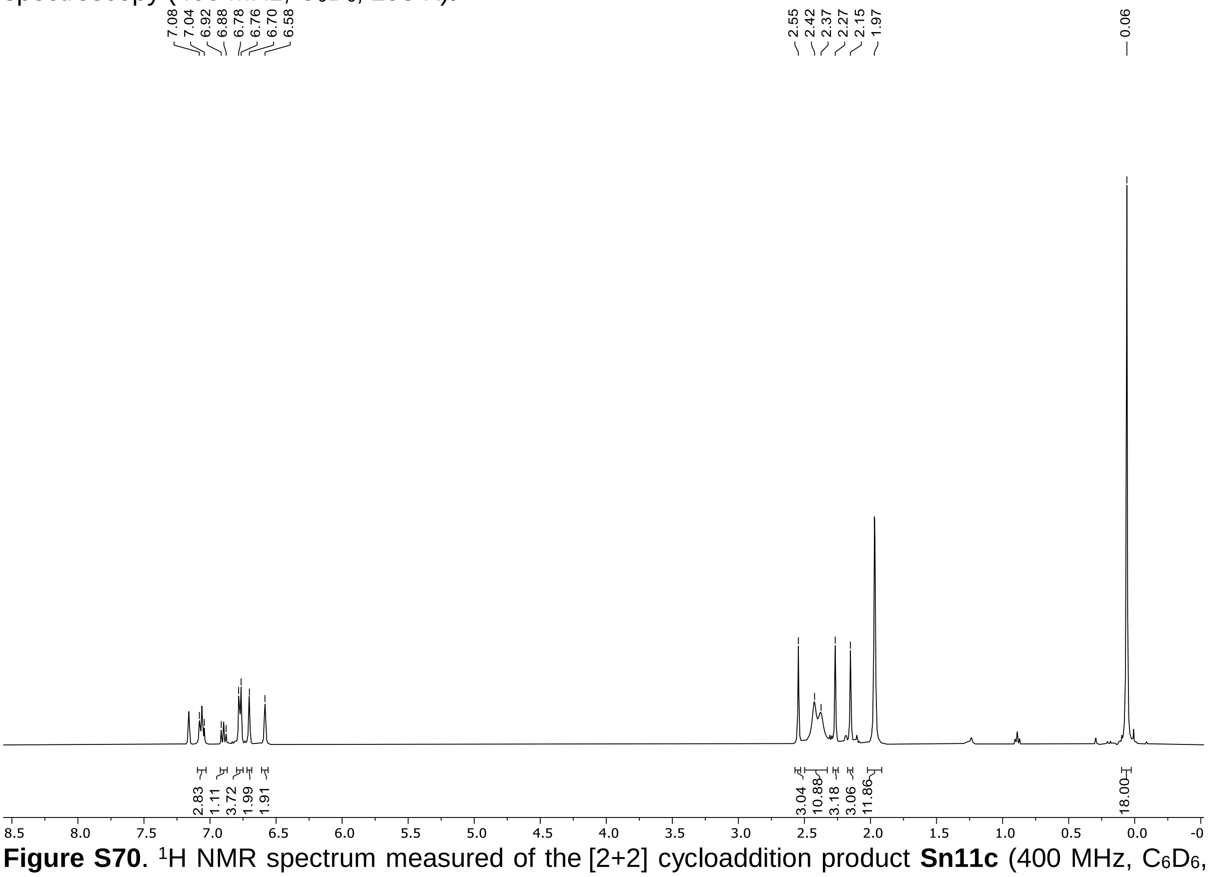

Figure S70. ^1H NMR spectrum measured of the [2+2] cycloaddition product **Sn11c** (400 MHz, C_6D_6, 298 K).

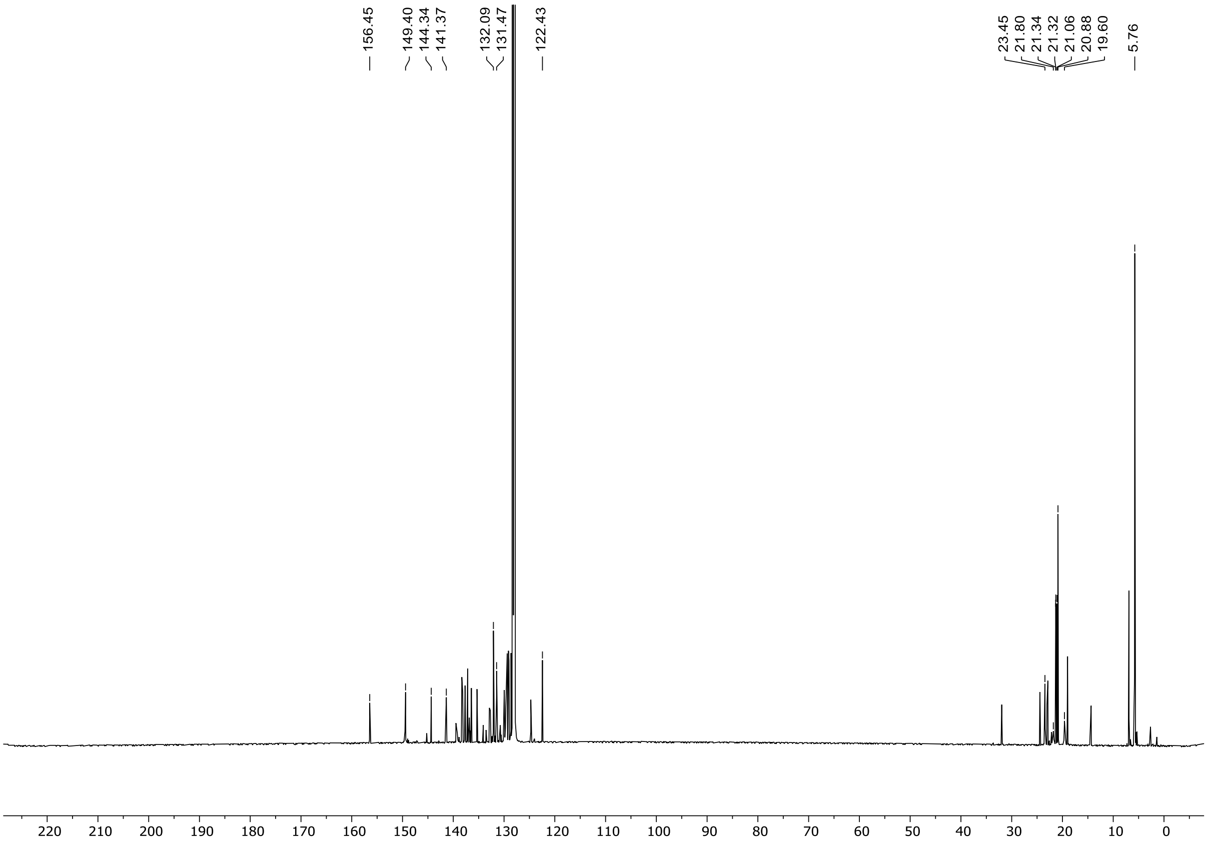

Figure S71. $^{13}C\{^1H\}$ NMR spectrum of a mixture of **Sn11c**, **Sn13** and MesN=C=NXyl (400 MHz, C_6D_6, 298 K); only signals of **Sn11c** picked.

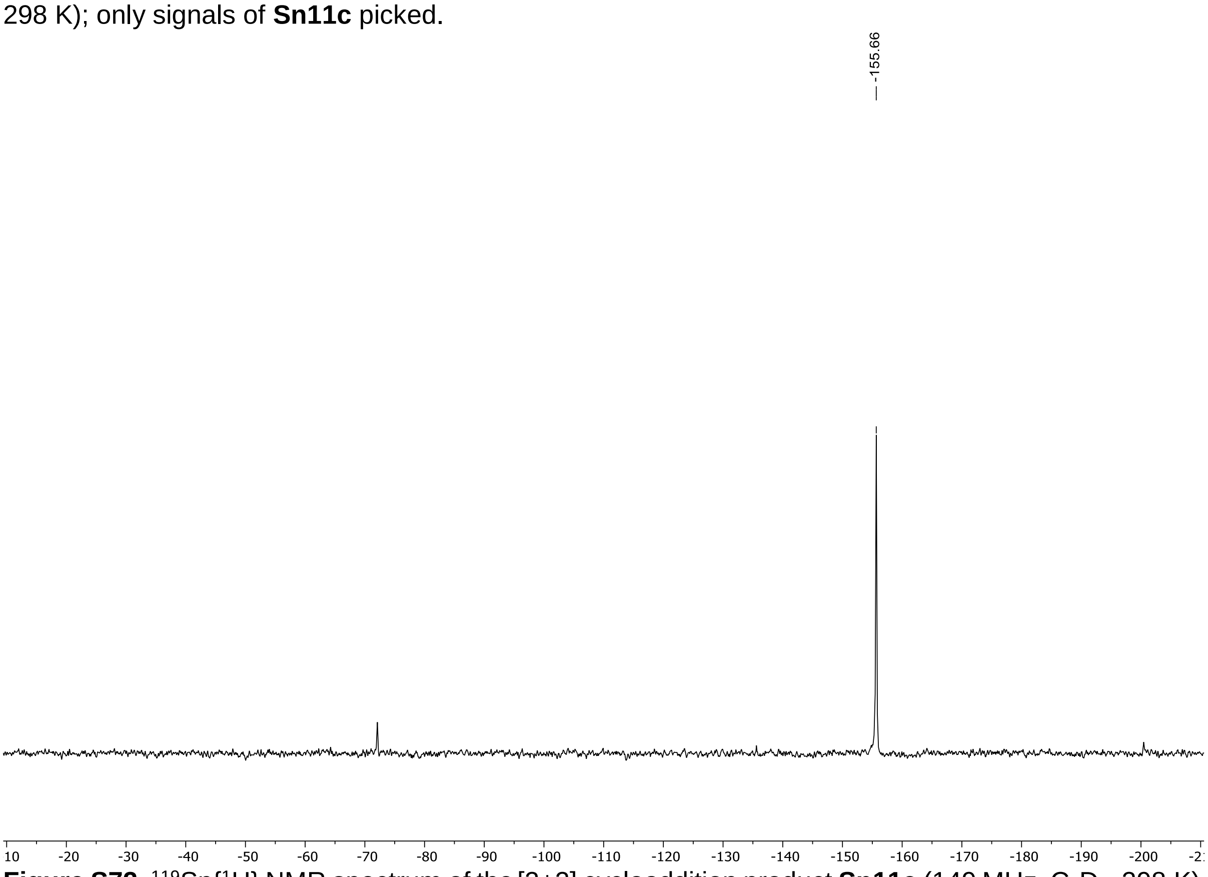

Figure S72. $^{119}Sn\{^1H\}$ NMR spectrum of the [2+2] cycloaddition product **Sn11c** (149 MHz, C_6D_6, 298 K).

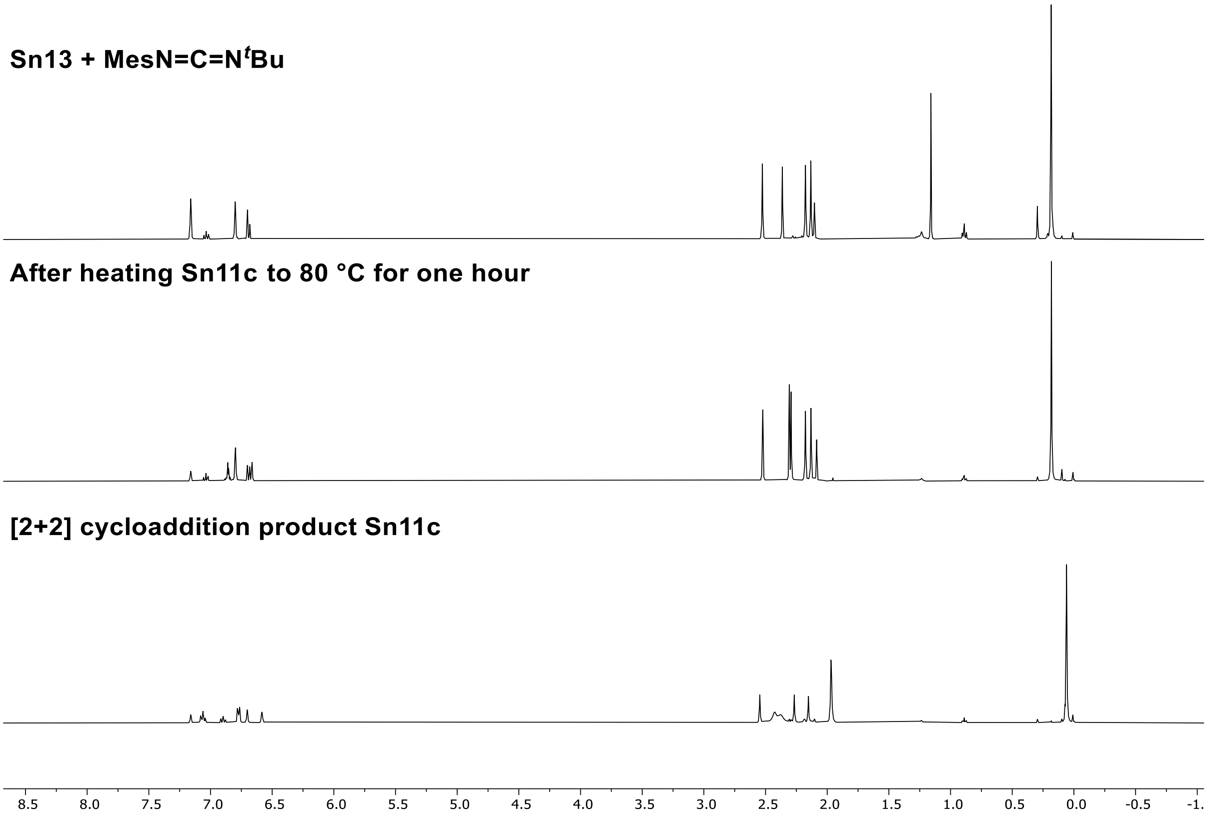

Figure S73. Formation of (^{Mes}TerSn(hmds)S)$_2$ (**Sn13**) and MesN=C=NXyl by heating the [2+2] cycloaddition product **Sn11c** to 80 °C as proven by ^{1}H NMR spectroscopy and ^{1}H NMR spectrum of the reaction of ^{Mes}TerSnN(SiMe$_3$)$_2$ with SCN^{t}Bu for reference (400 MHz, C_6D_6, 298 K).

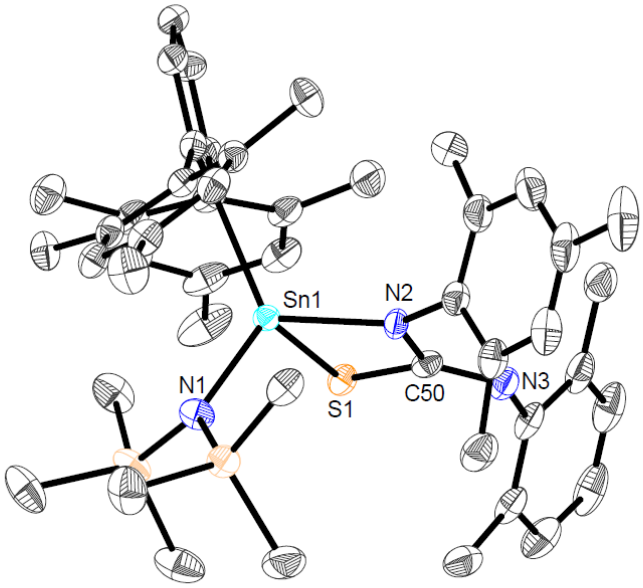

Figure S74. Molecular structure of the [2+2] cycloaddition product **Sn11c** in the crystal. Thermal ellipsoids are drawn at the 50% probability level (hydrogen atoms have been omitted for clarity). Selected bond lengths (Å) and angles (deg): Sn1–N1 2.046(4), Sn1–N2 2.110(4), Sn1–S1 2.4123(10), S1–C50 1.803(5), N2–C50 1.394(6), N3–C50 1.261(6), S1–Sn1–N2 70.60(10).

Reaction of $^{Mes}TerSn(N(SiMe_3)_2)=NMes$ (Sn7a) with SCN^tBu

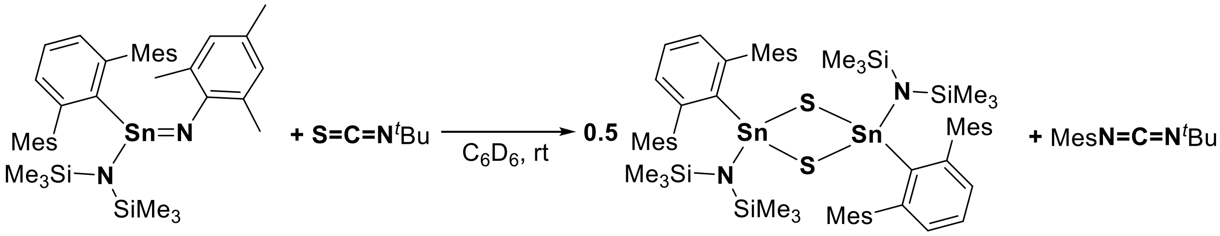

To a solution of $^{Mes}TerSnN(SiMe_3)_2$ (**Sn1**) (0.037 g, 0.062 mmol) in 0.3 mL of C_6D_6 was added a solution of N_3Mes (**A2**) (0.010 g, 0.062 mmol) in 0.3 mL of C_6D_6 for the *in situ* generation of $^{Mes}TerSn(N(SiMe_3)_2)=NMes$ (**Sn7a**). SCN^tBu (0.007 g, 0.062 mmol) in 0.1 mL of C_6D_6 was added and the reaction progress was monitored by ^1H NMR spectroscopy until $^{Mes}TerSn(N(SiMe_3)_2)=NMes$ (**Sn7a**) was consumed. All volatile components were removed under vacuum, the residue was dissolved in 0.7 mL of *n*-hexane, filtered and stored at -30 °C to give colourless crystals suitable for single crystal X-ray diffraction revealing the formation of $^{Mes}TerSn(hmds)S)_2$ (**Sn13**). The remaining material was dried under vacuum, dissolved in 0.6 mL of C_6D_6 and used for NMR characterization verifying the formation of $MesN=C=N^tBu$ as the second product. The obtained NMR data of $MesN=C=N^tBu$ are in good accordance to the literature data in $CDCl_3$.[S6]

NMR data of $^{Mes}TerSn(hmds)S)_2$ (**Sn13**):
^1H NMR (400 MHz, C_6D_6, 298 K): δ = 0.18 (s, 36H, $Si(CH_3)_3$), 2.13 (s, 12H, CH_3), 2.18 (s, 12H, CH_3), 2.52 (s, 12H, CH_3), 6.69-6.70 (m, 4H, CH_{Aryl})*, 6.80 (s, 8H, CH_{Aryl}), 7.02-7.05 (m, 2H, CH_{Aryl}) ppm.
* = overlap with CH_{Aryl} signal of $MesN=C=N^tBu$.
$^{13}C\{^1H\}$ NMR (101 MHz, C_6D_6, 298 K): δ = 6.9 ($Si(CH_3)_3$), 21.1 (CH_3), 22.9 (CH_3), 24.4 (CH_3), 129.4 (CH_{Aryl}), 130.7 (CH_{Aryl}), 131.4 (CH_{Aryl}), 136.8 ($C_{q,Aryl}$), 137.7 ($C_{q,Aryl}$), 138.4 ($C_{q,Aryl}$), 139.5 ($C_{q,Aryl}$), 145.2 ($C_{q,Aryl}$), 149.3 ($C_{q,Aryl}$) ppm.
$^{29}Si\{^1H\}$ NMR (80 MHz, C_6D_6, 298 K): δ = 5.5 ppm. (assigned by $^1H/^{29}Si$ HMBC)
$^{119}Sn\{^1H\}$ NMR (149 MHz, C_6D_6, 298 K): δ = -72.2 ppm.

NMR data of $MesN=C=N^tBu$:
^1H NMR (400 MHz, C_6D_6, 298 K): δ = 1.16 (s, 9H, $C_q(CH_3)_3$), 2.10 (s, 3H, CH_3), 2.36 (s, 6H, CH_3), 6.70 (s, 2H, CH_{Aryl})* ppm.
* = overlap with CH_{Aryl} signal of $^{Mes}TerSn(hmds)S)_2$ (**Sn13**).
$^{13}C\{^1H\}$ NMR (101 MHz, C_6D_6, 298 K): δ = 19.3 (CH_3), 20.8 (CH_3), 31.3 ($C_q(\underline{C}H_3)_3$), 55.6 ($\underline{C}_q(CH_3)_3$), 129.2 (CH_{Aryl}), 132.5 (C_q), 133.0 (C_q), 133.5 (C_q), 134.9 (C_q) ppm.

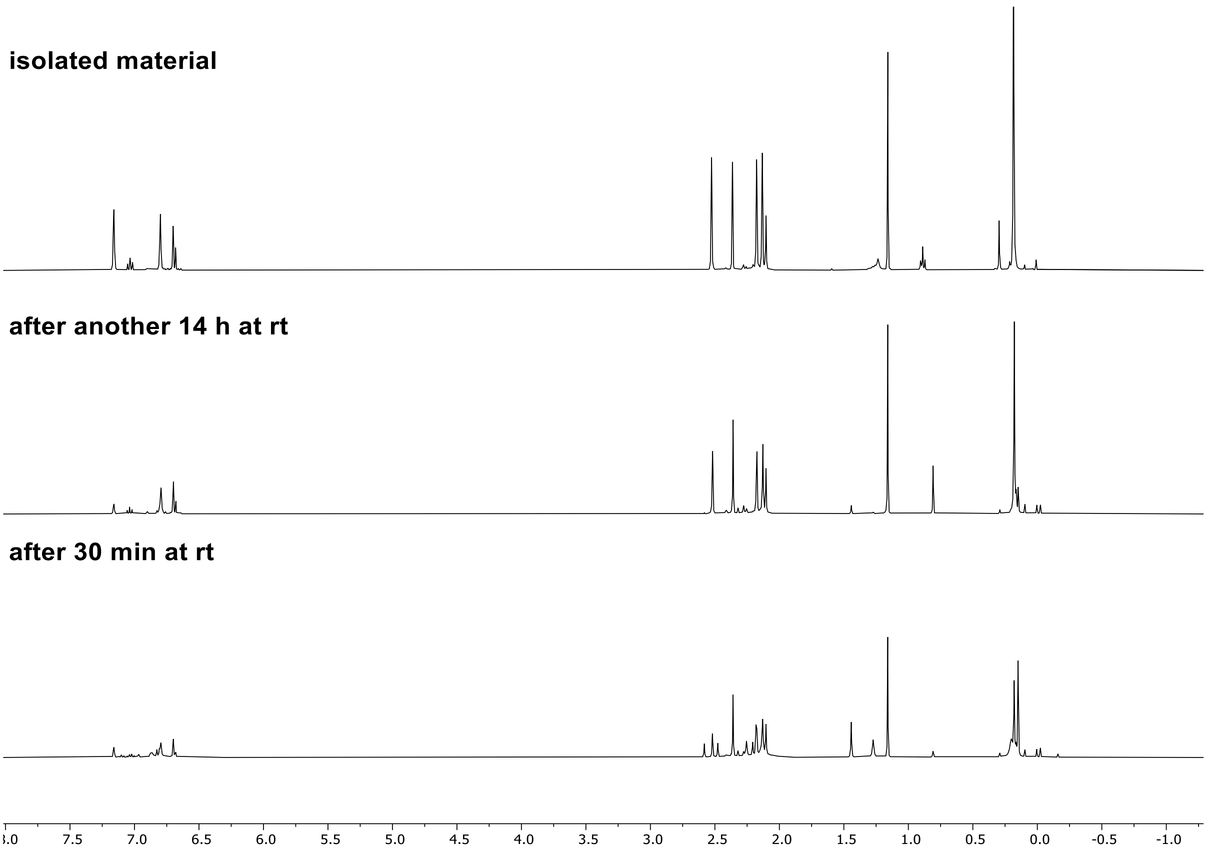

Figure S75. Monitoring of the reaction of ^{Mes}TerSnN(SiMe$_3$)$_2$ (**Sn1**) with SCN^{t}Bu via ^{1}H NMR spectroscopy (400 MHz, C$_6$D$_6$, 298 K).

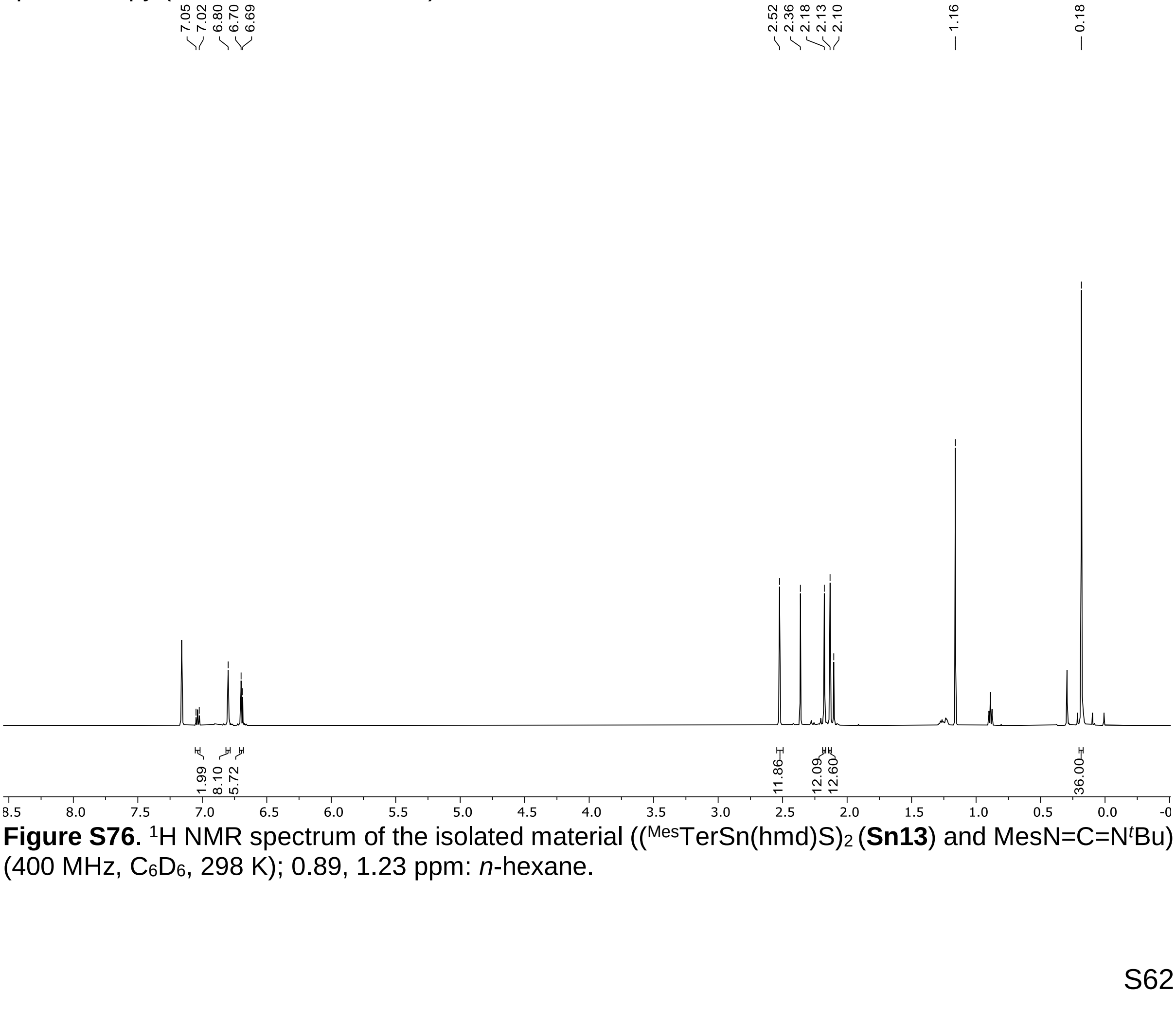

Figure S76. ^{1}H NMR spectrum of the isolated material ((^{Mes}TerSn(hmd)S)$_2$ (**Sn13**) and MesN=C=N^{t}Bu) (400 MHz, C$_6$D$_6$, 298 K); 0.89, 1.23 ppm: *n*-hexane.

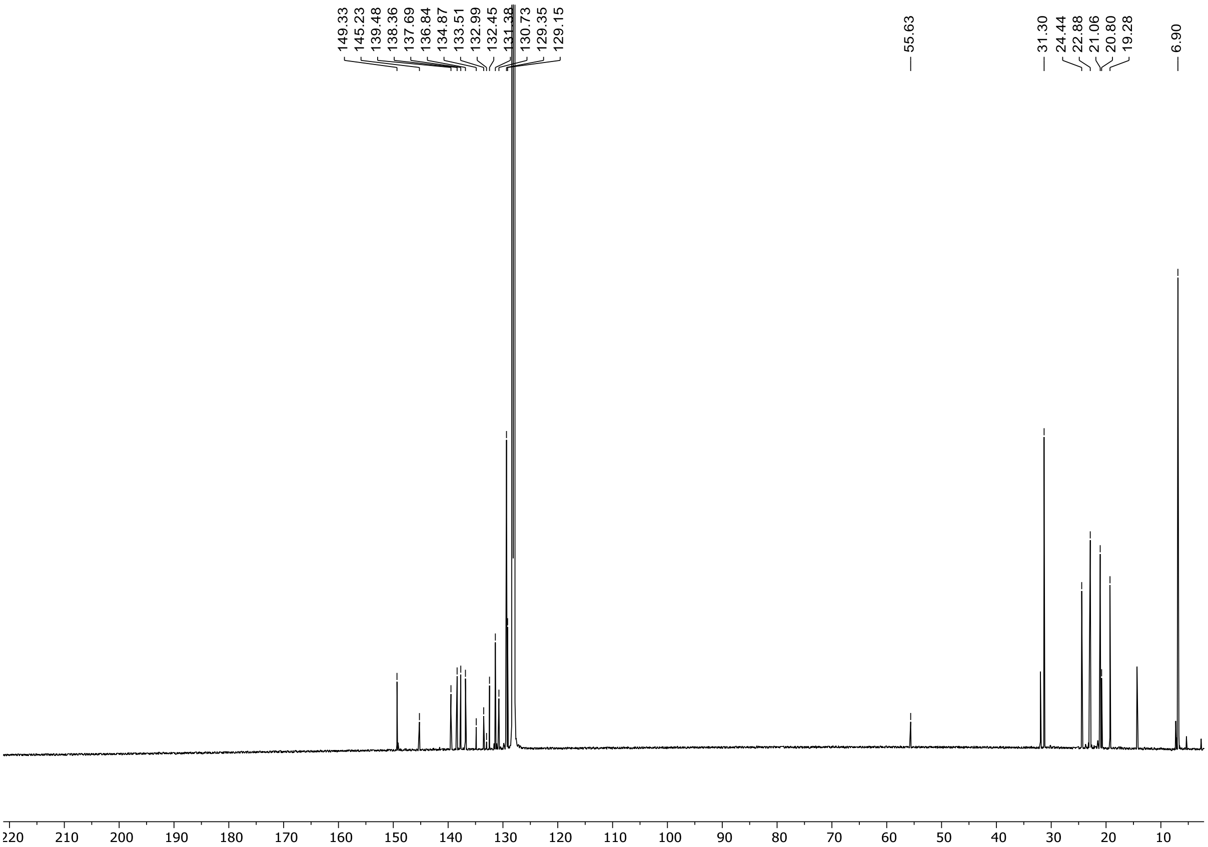

Figure S77. ^{13}C{^{1}H} NMR spectrum of the isolated material ((^{Mes}TerSn(hmd)S)$_2$ (**Sn13**) and MesN=C=N^{t}Bu) (400 MHz, C_6D_6, 298 K); 14.3, 23.0, 32.0 ppm: *n*-hexane.

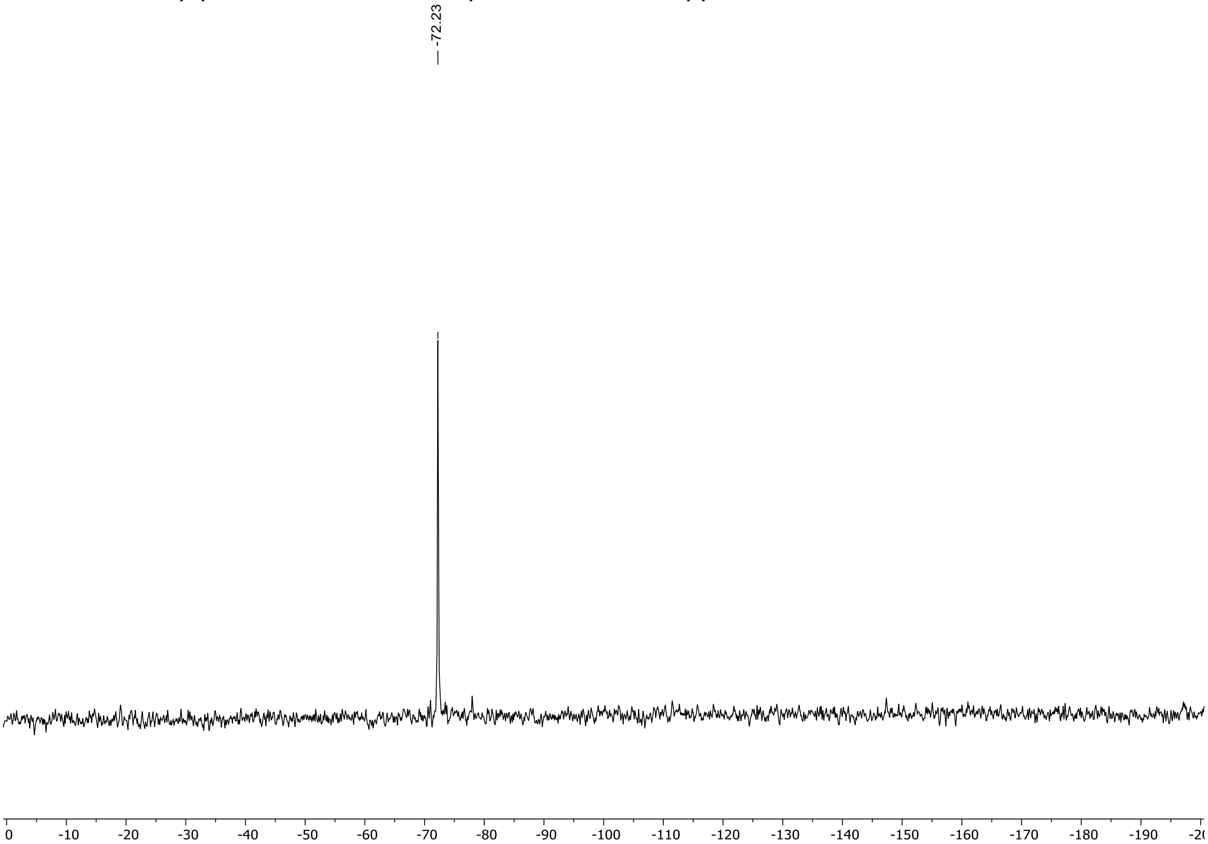

Figure S78. ^{119}Sn{^{1}H} NMR spectrum of (^{Mes}TerSn(hmd)S)$_2$ (**Sn13**) (149 MHz, C_6D_6, 298 K).

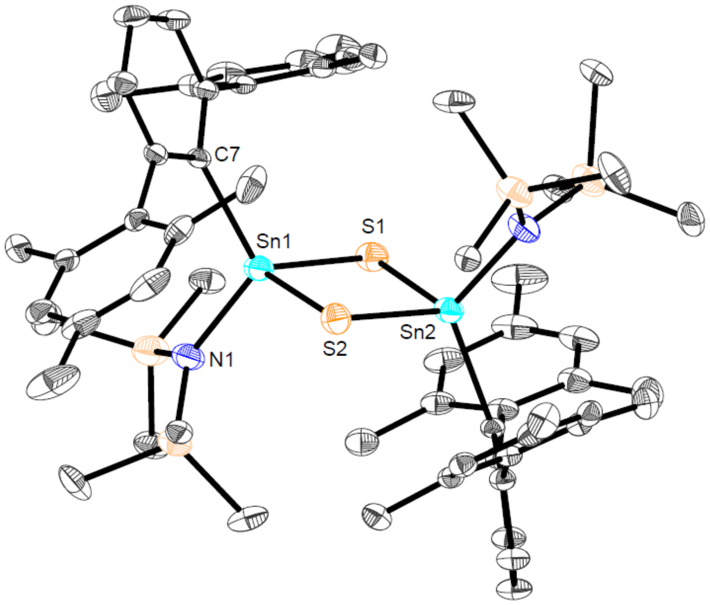

Figure S79. Molecular structure of (^{Mes}TerSn(hmds)S)$_2$ (**Sn13**) in the crystal. Thermal ellipsoids are drawn at the 50% probability level (hydrogen atoms have been omitted for clarity). Selected bond lengths (Å) and angles (deg): Sn1–S1 2.4218(5), Sn1–S2 2.4218(5), Sn2–S1 2.4225(5), Sn2–S2 2.4204(4), Sn1–N1 2.0627(16), Sn1–C7 2.2010(18), N1–Sn1–C7 115.68(7), Sn1–S1–Sn2 87.965(15), S1–Sn1–S2 91.990(16).

Crystallographic Details

Single crystal X-ray diffraction data for al compounds were collected at 150 K on an Oxford Diffraction/Agilent SuperNova diffractometer using Cu-K_{α} radiation (λ = 1.54184 Å) or Mo-K_{α} radiation (λ = 0.71073 Å), and equipped with a nitrogen gas Oxford Cryosystems cooling unit.[S5] Raw frame data were reduced using CrysAlisPro.[S6] The structures were solved using SHELXT[S7] and refined to convergence on F^2 by full-matrix least-squares using SHELXL[S8] in combination with OLEX2.[S9] Distances and angles were calculated using the full covariance matrix. Restraints were used to maintain sensible geometries for the disordered groups and approximate the displacement parameters to typical values. Selected crystallographic data are summarized in tables S2-S4 and full details are given in the supplementary deposited CIF files (CCDC 2191482-2191501). These data can be obtained free of charge from the Cambridge Crystallographic Data Centre via https://www.ccdc.cam.ac.uk/data_request/cif.

Table S1. Crystal structure data for compounds **Sn2**, **Sn4a** and **Sn3b**.

	**Sn2**	**Sn4a**	**Sn3b**
CCDC	2191484	2191492	2191488
empirical formula	$C_{33}H_{52}Si_4Sn$	$C_{36}H_{61}NSi_5Sn$	$C_{42}H_{63}NSi_4Sn$
fw	679.79	766.99	812.98
colour	blue	colourless	orange
habit	prism	block	block
cryst dimens, mm	0.20 x 0.10 x 0.05	0.32 x 0.22 x 0.19	0.15 x 0.10 x 0.10
cryst syst	monoclinic	monoclinic	monoclinic
space group	$P2_1/n$	C2/c	$P2_1/c$
a, Å	11.4765(2)	36.8577(2)	19.4787(3)
b, Å	19.3820(4)	10.64680(10)	16.2561(2)
c, Å	16.2836(3)	21.49590(10)	28.1054(3)
α, deg	90	90	90
β, deg	91.314(2)	98.3370(10)	96.5070(10)
γ, deg	90	90	90
V, $Å^3$	3621.13(12)	8346.20(10)	8842.2(2)
Z	4	8	8
D_{calc}, g•cm^{-3}	1.247	1.221	1.221
μ, mm^{-1}	7.012	6.412	5.833
T, K	150.01(10)	150.01(15)	150.01(16)
θ range, deg	3.546 – 76.437	4.157 – 76.169	3.146 – 76.441
no. of rflns collected	7514	8690	18256
no. of indep rflns	6714	8122	15061
R(int)	0.0478	0.0390	0.0444
max, min transmission	1.00000 and 0.62043	1.000 and 0.628	1.00000 and 0.82394
[I>2σ(I)]	R1 = 0.0357	R1 = 0.0232	R1 = 0.0360
	wR2 = 0.0927	wR2 = 0.0591	wR2 = 0.0839
R indices (all data)	R1 = 0.0407	R1 = 0.0257	R1 = 0.0482
	wR2 = 0.0966	wR2 = 0.0611	wR2 = 0.0909
GOF on F^2	1.047	1.062	1.013

Table S2. Crystal structure data for compounds **Sn3c**, **Sn5** and **Sn6**.

	**Sn3c**	**Sn5**	**Sn6**
CCDC	2191500	2191489	2191499
empirical formula	$C_{45}H_{69}NSi_4Sn$	$C_{57}H_{62}N_6Si_3Sn(0.5\ C_6H_{14})$	2 $C_{36}H_{61}N_5Si_4Sn$
fw	855.06	1077.17	1589.89
colour	orange	clear red	clear yellow
habit	plate	block	block
cryst dimens, mm	0.30 x 0.30 x 0.05	0.41 x 0.37 x 0.27	0.30 x 0.10 x 0.10
cryst syst	triclinic	triclinic	orthorhombic
space group	P1	P1	$P2_12_12_1$
a, Å	13.1701(3)	12.4031(2)	16.30110(10)
b, Å	19.2384(6)	14.1601(2)	16.42630(10)
c, Å	22.0869(8)	18.0463(3)	30.6222(2)
α, deg	115.264(3)	86.8750(10)	90
β, deg	90.275(2)	75.435(2)	90
γ, deg	101.094(2)	71.289(2)	90
V, $Å^3$	4942.5(3)	2904.29(9)	8199.61(9)
Z	4	2	4
D_{calc}, g•cm^{-3}	1.149	1.232	1.288
μ, mm^{-1}	5.240	0.543	6.305
T, K	150.01(10)	150.00(10)	150.01(10)
θ range, deg	3.906 – 76.458	2.465 – 28.281	3.820 – 76.362
no. of rflns collected	20426	14338	17100
no. of indep rflns	17414	13145	16435
R(int)	0.0533	0.0286	0.0585
max, min transmission	1.00000 and 0.63781	1.000 and 0.511	1.00000 and 0.32447
[$I>2\sigma(I)$]	R1 = 0.0376<br>wR2 = 0.0937	R1 = 0.0282<br>wR2 = 0.0706	R1 = 0.0252<br>wR2 = 0.0575
R indices (all data)	R1 = 0.0453<br>wR2 = 0.0989	R1 = 0.0322<br>wR2 = 0.0730	R1 = 0.0274<br>wR2 = 0.0589
GOF on F^2	1.013	1.025	1.058

Table S3. Crystal structure data for compounds **Sn7a**, **Sn4b** and **Sn4c**.

	**Sn7a**	**Sn4b**	**Sn4c**
CCDC	2191486	2191482	2191485
empirical formula	$C_{39}H_{54}N_2Si_2Sn$	$C_{39}H_{54}N_2Si_2Sn$	$C_{42}H_{60}N_2Si_2Sn$
fw	725.71	725.71	767.79
colour	red	clear colourless	clear yellow
habit	block	block	block
cryst dimens, mm	0.40 x 0.30 x 0.30	0.20 x 0.10 x 0.10	0.20 x 0.20 x 0.10
cryst syst	monoclinic	triclinic	monoclinic
space group	$P2_1/n$	P1	$P2_1/c$
a, Å	10.13650(10)	9.4610(3)	9.96740(10)
b, Å	21.5725(2)	10.4356(4)	18.4881(2)
c, Å	17.68170(10)	19.2720(7)	22.0455(2)
α, deg	90	94.536(3)	90
β, deg	93.9610(10)	95.873(3)	97.1410(10)
γ, deg	90	90.395(3)	90
V, $Å^3$	3857.21(6)	1886.62(12)	4030.99(7)
Z	4	2	4
D_{calc}, g•cm^{-3}	1.250	1.279	1.265
μ, mm^{-1}	6.059	0.769	5.825
T, K	150.01(10)	150.00(10)	150.01(10)
θ range, deg	4.806 – 76.415	3.558 – 30.574	4.042 – 76.394
no. of rflns collected	8029	9935	8405
no. of indep rflns	7607	8359	7969
R(int)	0.0413	0.0422	0.0297
max, min transmission	1.00000 and 0.51855	1.00000 and 0.96160	1.00000 and 0.73892
[I>2σ(I)]	R1 = 0.0261	R1 = 0.0397	R1 = 0.0215
	wR2 = 0.0662	wR2 = 0.0729	wR2 = 0.0539
R indices (all data)	R1 = 0.0279	R1 = 0.0532	R1 = 0.0231
	wR2 = 0.0676	wR2 = 0.0806	wR2 = 0.0551
GOF on F^2	1.056	1.064	1.026

Table S4. Crystal structure data for compounds **Sn10a**, **Sn10b** and **Sn11b**.

	**Sn10a**	**Sn10b**	**Sn11b**
CCDC	2191493	2191495	2191491
empirical formula	$C_{47}H_{60}N_2Si_2Sn$	$C_{45}H_{63}ClN_2Si_2Sn$	$C_{46}H_{68}N_4Si_2Sn$
fw	827.84	842.29	851.91
colour	colourless	colourless	colourless
habit	plate	block	block
cryst dimens, mm	0.15 x 0.10 x 0.02	0.20 x 0.10 x 0.10	0.50 x 0.50 x 0.15
cryst syst	monoclinic	monoclinic	monoclinic
space group	$P2_1/c$	$P2_1/c$	$P2_1/c$
a, Å	18.8402(8)	11.2439(4)	16.8086(7)
b, Å	20.5363(4)	20.9987(7)	11.0222(4)
c, Å	29.8743(12)	18.3986(5)	25.0170(10)
α, deg	90	90	90
β, deg	131.799(7)	95.725(3)	101.001(4)
γ, deg	90	90	90
V, Å ^{3}	8616.8(9)	4322.4(2)	4549.7(3)
Z	8	4	4
D_{calc}, g•cm^{-3}	1.276	1.294	1.244
μ, mm^{-1}	5.492	0.741	0.649
T, K	150.01(10)	150.01(10)	150.00(10)
θ range, deg	3.693 – 76.387	2.660 – 30.645	2.628 – 30.618
no. of rflns collected	17933	11757	12199
no. of indep rflns	14723	8336	7502
R(int)	0.1321	0.0524	0.0553
max, min transmission	1.00000 and 0.50574	1.00000 and 0.79518	1.00000 and 0.97489
[I>2σ(I)]	R1 = 0.1606	R1 = 0.0476	R1 = 0.0458
	wR2 = 0.4300	wR2 = 0.0815	wR2 = 0.0768
R indices (all data)	R1 = 0.1733	R1 = 0.0824	R1 = 0.0956
	wR2 = 0.4359	wR2 = 0.0962	wR2 = 0.0955
GOF on F^2	1.067	1.046	1.022

Table S5. Crystal structure data for compounds **Sn12**, **Sn8** and **Sn4d**.

	**Sn12**	**Sn8**	**Sn4d**
CCDC	2191496	2191494	2191483
empirical formula	2 $C_{40}H_{54}N_2S_2Si_2Sn(1.0\ C_6H_6)$	$C_{39}H_{49}N_3Si_2Sn$	$C_{39}H_{49}N_3Si_2Sn$
fw	1681.79	734.68	734.68
colour	red	red	clear yellow
habit	block	prism	block
cryst dimens, mm	0.26 x 0.22 x 0.17	0.15 x 0.15 x 0.10	0.32 x 0.24 x 0.19
cryst syst	monoclinic	monoclinic	monoclinic
space group	$P2_1$	$P2_1$	$P2_1/n$
a, Å	10.74720(10)	10.1365(4)	12.52680(10)
b, Å	32.0622(3)	14.2491(6)	15.89970(10)
c, Å	12.40870(10)	13.6133(6)	18.3237(2)
α, deg	90	90	90
β, deg	101.4410(10)	106.620(5)	93.3020(10)
γ, deg	90	90	90
V, Å ^{3}	4190.81(7)	1884.11(14)	3643.52(5)
Z	2	2	4
D_{calc}, g•cm^{-3}	1.333	1.295	1.339
μ, mm^{-1}	6.560	0.771	6.433
T, K	150.00(10)	150.01(10)	150.00(10)
θ range, deg	3.634 – 76.598	3.435 – 30.442	3.683 – 76.366
no. of rflns collected	8956	8832	7582
no. of indep rflns	8574	7821	7274
R(int)	merged	0.0410	0.0263
max, min transmission	1.00000 and 0.74808	1.000 and 0.989	1.000and 0.993
[I>2σ(I)]	R1 = 0.2558<br>wR2 = 0.0975	R1 = 0.0359<br>wR2 = 0.0661	R1 = 0.0210<br>wR2 = 0.0531
R indices (all data)	R1 = 0.2623<br>wR2 = 0.0997	R1 = 0.0464<br>wR2 = 0.0705	R1 = 0.0221<br>wR2 = 0.0537
GOF on F^2	1.300	1.045	1.043

Table S6. Crystal structure data for compounds **Sn9**, **Sn3a** and **Sn11a**.

	**Sn9**	**Sn3a**	**Sn11a**
CCDC	2191487	2191498	2191490
empirical formula	$C_{66}H_{60}N_4Sn_2$	$C_{36}H_{61}NSi_5Sn$	$C_{40}H_{54}N_2O_2Si_2Sn(0.5\ C_6H_6)$
fw	1146.56	766.99	808.77
colour	red	clear yellow	colourless
habit	block	needle	plate
cryst dimens, mm	0.35 x 0.29 x 0.17	0.22 x 0.08 x 0.07	0.27 x 0.17 x 0.04
cryst syst	monoclinic	triclinic	triclinic
space group	$P2_1/n$	P1	P1
a, Å	10.5033(2)	10.6743(3)	8.6118(2)
b, Å	19.8081(3)	12.6354(4)	10.3042(2)
c, Å	12.4466(2)	16.0801(5)	24.6552(5)
α, deg	90	90.543(2)	86.901(2)
β, deg	91.824(2)	93.054(2)	80.351(2)
γ, deg	90	105.893(3)	68.512(2)
V, Å ^{3}	2588.21(8)	2082.27(11)	2006.91(8)
Z	2	2	2
D_{calc}, g•cm^{-3}	1.471	6.426	1.338
μ, mm^{-1}	8.026	1.223	5.919
T, K	150.00(10)	150.00(12)	149.98(13)
θ range, deg	4.197 – 76.254	3.638 – 76.296	3.637 – 76.378
no. of rflns collected	5362	8616	12771
no. of indep rflns	4986	7778	11224
R(int)	0.0271	0.0449	merged
max, min transmission	1.000 and 0.989	1.000 and 0.988	0.999 and 0.989
[I>2σ(I)]	R1 = 0.0234<br>wR2 = 0.0610	R1 = 0.0294<br>wR2 = 0.0715	R1 = 0.0296<br>wR2 = 0.0732
R indices (all data)	R1 = 0.0257<br>wR2 = 0.0624	R1 = 0.0344<br>wR2 = 0.0743	R1 = 0.0359<br>wR2 = 0.0758
GOF on F^2	1.020	1.043	0.983

Table S7. Crystal structure data for compounds **Sn13**, and **Sn11c**.

	**Sn13**	**Sn11c**
CCDC	2191501	2191497
empirical formula	$C_{60}H_{86}N_2S_2Si_4Sn_2(2.0\ C_6H_{14})$	$C_{48}H_{63}N_3SSi_2Sn$
fw	1421.16	888.94
colour	colourless	colourless
habit	plate	plate
cryst dimens, mm	0.39 x 0.10 x 0.06	0.35 x 0.13 x 0.04
cryst syst	monoclinic	orthorhombic
space group	I2/a	$Pna2_1$
a, Å	22.29380(10)	22.1978(4)
b, Å	15.04860(10)	11.9903(2)
c, Å	42.5084(2)	17.3660(2)
α, deg	90	90
β, deg	90.1440(10)	90
γ, deg	90	90
V, $Å^3$	14261.12(13)	4622.10(13)
Z	8	4
D_{calc}, g•cm^{-3}	1.164	1.277
μ, mm^{-1}	7.002	5.572
T, K	150.01(18)	150.00(10)
θ range, deg	3.115 – 76.294	3.983 – 76.249
no. of rflns collected	14884	7562
no. of indep rflns	12934	6753
R(int)	0.0593	0.0462
max, min transmission	1.000 and 0.966	1.000 and 0.994
[I>2σ(I)]	R1 = 0.0245<br>wR2 = 0.0617	R1 = 0.0304<br>wR2 = 0.0668
R indices (all data)	R1 = 0.0309<br>wR2 = 0.0650	R1 = 0.0383<br>wR2 = 0.0708
GOF on F^2	1.023	1.018

Computational Details

All computations were carried out using the Gaussian 16 software package or the ORCA 5.0 software package.[S12,S13] Gas phase optimizations and frequency analyses were carried out using the M06-2X functional[S14] and the def2-SVP basis set.[S15] Each system was treated with a Grimme dispersion correction with Becke-Johnson damping (GD3BJ).[S16] The optimized structures were confirmed to be minima on the potential energy surface by the absence of imaginary frequencies. Transition states were confirmed to be local energy maxima by the presence of a single imaginary frequency along the bond forming/breaking path. Furthermore, an intrinsic reaction coordinate calculation in the forward and back directions was performed. Natural bonding orbital (NBO) analyses were carried out using the NBO 7.0 program.[S17] Atoms in molecules (AIM) analyses were conducted using the AIMAll software package.[S18]

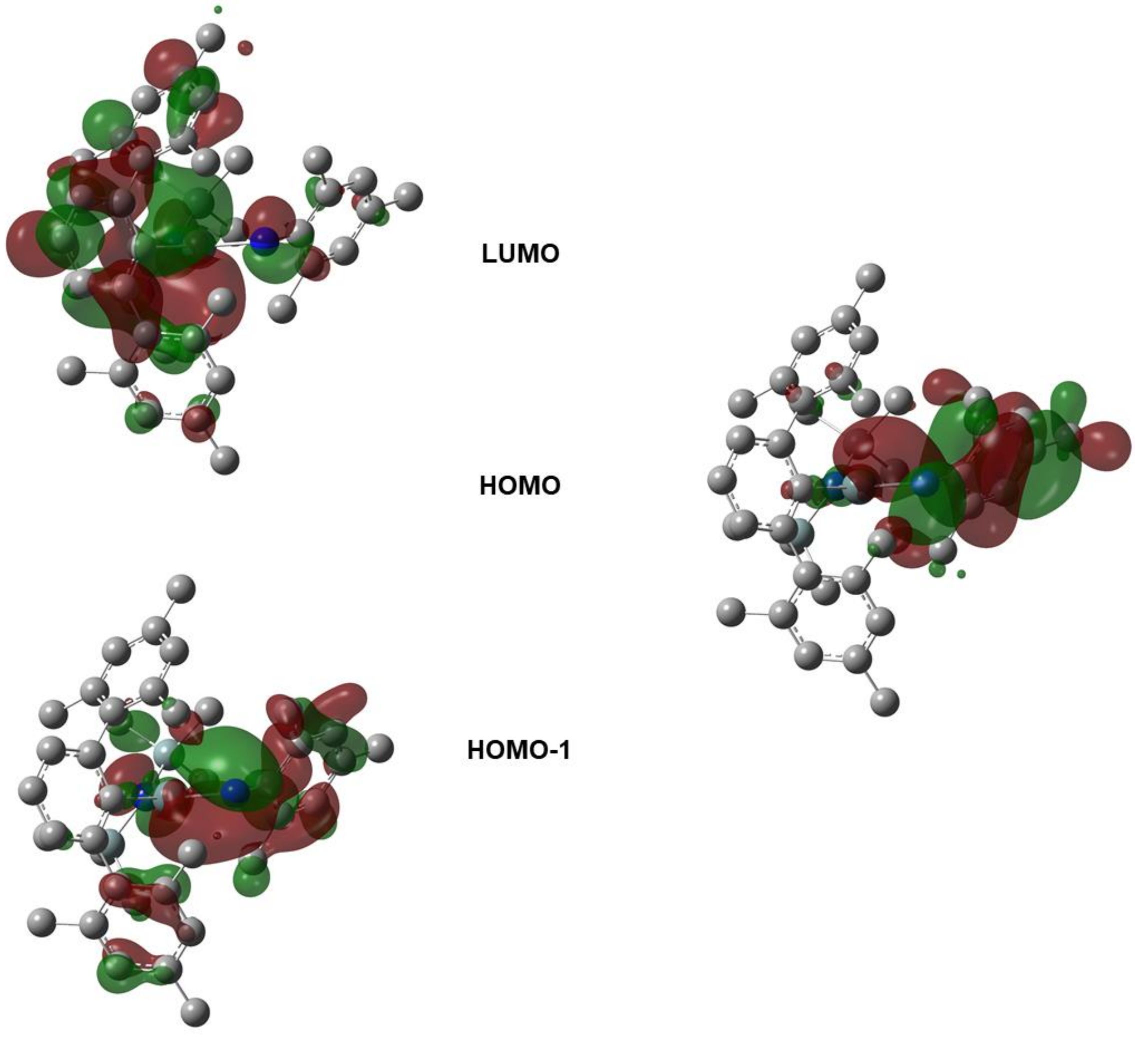

Figure S80. Selected molecular orbitals of the optimized structure of **Sn7a** revealing the major bonding within the SnN moiety.

Table S8. NBOs containing bonding interactions between tin and phosphorus in **Sn7a**. Hydrogen atoms were omitted for clarity.

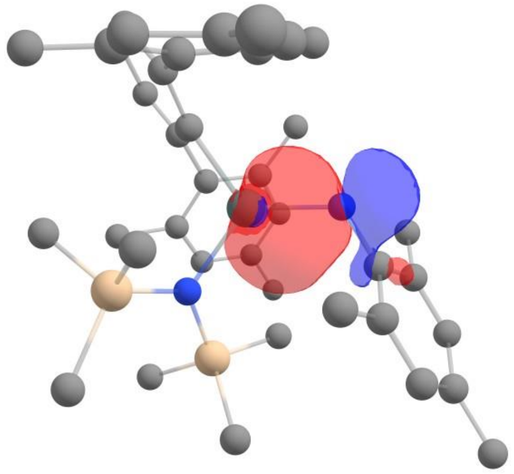	**NBO 9**<br>Occupancy: 1.86559 e<br>22% Sn [**s** (11%), **p** (89%)]<br>78% N [**s** (54%), **p** (46%)]
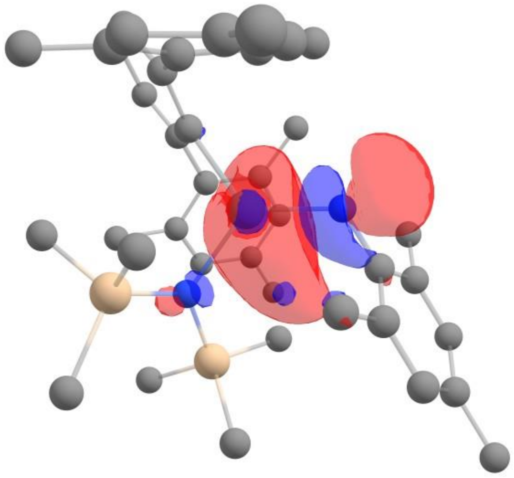	**NBO 804**<br>Occupancy: 0.19734 e<br>22% Sn [**s** (11%), **p** (89%)]<br>78% N [**s** (54%), **p** (46%)]

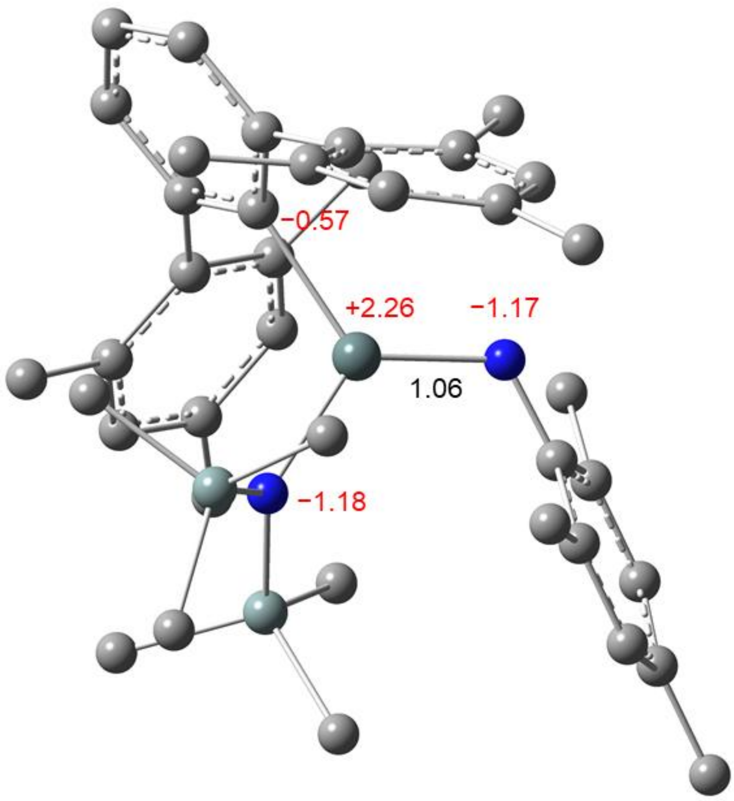

Figure S81. Optimized structure of **Sn7a** with the tin-phosphorus Wiberg bond index (shown in black) and selected natural atomic charges (shown in red).

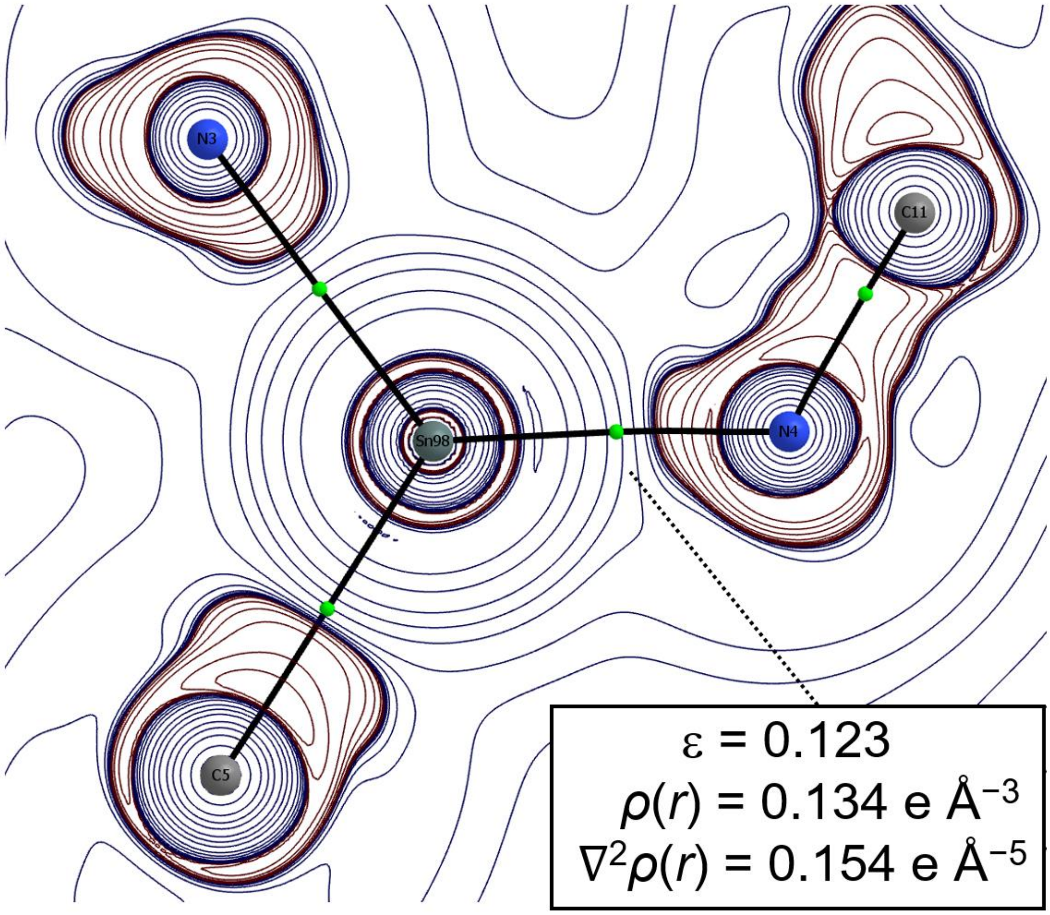

Figure S82. Contour plot of the Laplacian of the electron density in the plane of the stannaimine moiety (Sn98-N4) in **Sn7a** from AIM analysis**.** Bond critical points are shown in green, along with the ellipticity (ε), electron density [ρ(*r*)] and Laplacian of the electron density [$\nabla^2\rho(r)$] at the Sn98-N4 critical point.

References

[S1] M. Fischer, M. M. D. Roy, L. L. Wales, M. A. Ellwanger, A. Heilmann, S. Aldridge, *J. Am. Chem. Soc.* **2022**, *144*, 8908-8913.

[S2] C. Marschner, *Eur. J. Inorg. Chem.* **1998**, 221-226.

[S3] S.-K. Chen, W.-Q. Ma, Z.-B. Yan, F.-M. Zhang, S.-H. Wang, Y.-Q. Tu, X.-M. Zhang, J.-M. Tian, *J. Am. Chem. Soc.* **2018**, *140*, 10099-10103.

[S4] J. Gavenonis, T. D. Tilley, *J. Am. Chem. Soc.* **2002**, *124*, 8536-8537.

[S5] Q. Liu, Y. Tor, *Org. Lett.* **2003**, *5*, 2571-2572.

[S6] S. Wiese, M. J. B. Aguila, E. Kogut, T. Warren, *Organometallics* **2013**, *32*, 2300-2308.

[S7] J. Cosier, A. M. Glazer, *J. Appl. Cryst.* **1986**, *19*, 105-107.

[S8] CrystAlisPro, Oxford Diffraction/Agilent Technologies UK Ltd, Yarnton, UK.

[S9] G. Sheldrick, *Acta Cryst. C* **2015**, *71*, 3-8.

[S10] G. Sheldrick, *Acta Cryst. A* **2008**, *64*, 112-122.

[S11] O. V. Dolomanov, L. J. Bourhis, R. J. Gildea, J. A. K. Howard, H. Puschmann, *J. Appl. Cryst.* **2009**, *42*, 339-341.

[S12] Gaussian 16, Revision C.01, M. J. Frisch, G. W. Trucks, H. B. Schlegel, G. E. Scuseria, M. A. Robb, J. R. Cheeseman, G. Scalmani, V. Barone, G. A. Petersson, H. Nakatsuji, X. Li, M. Caricato, A. V. Marenich, J. Bloino, B. G. Janesko, R. Gomperts, B. Mennucci, H. P. Hratchian, J. V. Ortiz, A. F. IzmaylovJ. , J. L. Sonnenberg, D. Williams-Young, F. Ding, F. Lipparini, F. Egidi, J. Goings, B. Peng, A. Petrone, T. Henderson, D. Ranasinghe, V. G. Zakrzewski, J. Gao, N. Rega, G. Zheng, W. Liang, M. Hada, M. Ehara, K. Toyota, R. Fukuda, J. Hasegawa, M. Ishida, T. Nakajima, Y. Honda, O. Kitao, H. Nakai, T. Vreven, K. Throssell, J. A. Montgomery, Jr., J. E. Peralta, F. Ogliaro, M. J. Bearpark, J. J. Heyd, E. N. Brothers, K. N. Kudin, V. N. Staroverov, T. A. Keith, R. Kobayashi, J. Normand, K. Raghavachari, A. P. Rendell, J. C. Burant, S. S. Iyengar, J. Tomasi, M. Cossi, J. M. Millam, M. Klene, C. Adamo, R. Cammi, J. W. Ochterski, R. L. Martin, K. Morokuma, O. Farkas, J. B. Foresman, and D. J. Fox, Gaussian, Inc., Wallingford CT, 2016.

[S13] F. Neese, F. Wennmohs, U. Becker, C. Riplinger, *J. Chem. Phys.* **2020**, *152*, 224108.

[S14] Y. Zhao and D. G. Truhlar, *Theor. Chem. Acc.* **2008**, *120*, 215.

[S15] F. Weigend, R. Ahlrichs, *Phys. Chem. Chem. Phys.* **2005**, *7*, 3297.

[S16] a) S. Grimme, J. Antony, S. Ehrlich, H. Krieg, *J. Chem. Phys.*, **2010**, *132*, 154104; b) S. Grimme, S. Ehrlich, L. Goerigk, *J. Comp. Chem.* **2011**, *32*, 1456.

[S17] NBO 7.0, E. D. Glendening, J, K. Badenhoop, A. E. Reed, J. E. Carpenter, J. A. Bohmann, C. M. Morales, P. Karafilogou, C. R. Landis, F. Weinhold, Theoretical Chemistry Institute, University of Wisconsin, Madison (2018).

[S18] AIMAll, Version 19.10.12), T. A. Keith, TK Gristmill Software, Overland Park KS, USA, 2019 (aim.tkgristmill.com).
